# C−H Activation Enables a Concise Total Synthesis of Quinine and Analogues with Enhanced Antimalarial Activity

**DOI:** 10.1002/anie.201804551

**Published:** 2018-06-22

**Authors:** Daniel H. O' Donovan, Paul Aillard, Martin Berger, Aurélien de la Torre, Desislava Petkova, Christian Knittl‐Frank, Danny Geerdink, Marcel Kaiser, Nuno Maulide

**Affiliations:** ^1^ Institute of Organic Chemistry University of Vienna Währinger Straße 38 1090 Vienna Austria; ^2^ Swiss Tropical and Public Health Institute Socinstrasse 57 4002 Basel Switzerland; ^3^ University of Basel 4003 Basel Switzerland; ^4^ AstraZeneca Oncology, IMED Biotech Unit 1 Francis Crick Avenue Cambridge CB2 0RE UK

**Keywords:** C−H activation, malaria, quinine, total synthesis

## Abstract

We report a novel approach to the classical natural product quinine that is based on two stereoselective key steps, namely a C−H activation and an aldol reaction, to unite the two heterocyclic moieties of the target molecule. This straightforward and flexible strategy enables a concise synthesis of natural (−)‐quinine, the first synthesis of unnatural (+)‐quinine, and also provides access to unprecedented C3‐aryl analogues, which were prepared in only six steps. We additionally demonstrate that these structural analogues exhibit improved antimalarial activity compared with (−)‐quinine both in vitro and in mice infected with Plasmodium berghei.

Among the many natural products described throughout the chemical literature, quinine has undoubtedly one of the most vivid and colorful histories. For over a century, this cinchona alkaloid has attracted the attention of many researchers.^1^ Its potent antimalarial activity, especially against resistant strains,^2^ along with its privileged role in catalysis,^3^ have rendered it a highly coveted target. The first (formal) synthesis of the alkaloid, widely regarded as a milestone in total synthesis, was achieved by Woodward and Doering in 1944 and was heralded in the popular media.^4^, ^5^ Taken together, virtually all of the successful approaches to this natural product share as a common trait (Figure 1 a) the late‐stage construction of the quinuclidine moiety, namely through a C8−N1 disconnection.^6^ Despite the apparent structural simplicity of quinine, the first enantioselective synthesis was reported by Stork and co‐workers as late as 2001,^7^ with a longest linear sequence of 14 steps and featuring an innovative C6−N1 disconnection (Figure 1 b). Notably, no reported synthesis demonstrated the introduction of structural diversity at the C3 position although there are studies that suggest that this site can modulate antimalarial activity.^8^ Furthermore, to the best of our knowledge, there are no reports on either the synthesis or the biological activity of the unnatural enantiomer (+)‐quinine. Indeed, this enantiomer is conspicuously absent throughout the vast literature of organocatalysis, where the diastereoisomeric analogue quinidine is typically employed as a “pseudo”‐enantiomer.

**Figure 1:**
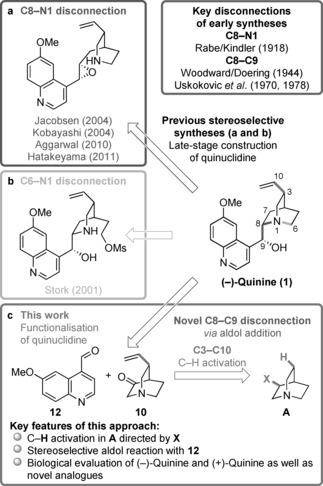
a,b) An overview of previous retrosynthetic approaches to quinine (**1**) and c) our synthetic plan. For a comprehensive review on prior syntheses, see Ref. 1a–1d.

Herein we report a straightforward and flexible strategy enabling the concise stereoselective synthesis of both enantiomers of quinine that is based on a selective C(sp^3^)−H activation step and a novel C8−C9 disconnection through an aldol addition. Furthermore, we disclose a short route towards novel C3‐aryl analogues and their in vivo biological evaluation, revealing significantly enhanced antimalarial activity.

Our synthetic plan predicated on the use of a preformed quinuclidine **A** bearing a C7 (quinine numbering) substituent **X** (Figure 1 c). This substituent should simultaneously 1) embody the directing group to guide our planned C(sp^3^)−H activation step while also 2) serving as a masked ketone to facilitate the aldol event and ultimately 3) being amenable to removal as a redundant functionality at the end of the synthesis.

We chose the picolinamide directing group pioneered by Daugulis and co‐workers for the C(sp^3^)−H activation reaction.^9^, ^10^ This moiety was readily introduced in a coupling reaction using commercially available 3‐aminoquinuclidine (**2**; Scheme 1).^11^ From intermediate **3**, we found that C(sp^3^)−H arylation using palladium catalysis^12^ can be achieved with complete regio‐ and diastereocontrol. The observed selectivity is likely the result of the preferential formation of a five‐membered palladacyclic intermediate, combined with preclusion of C(sp^3^)−H activation at C8 owing the inductive effect of the adjacent nitrogen atom. Although direct C(sp^3^)−H vinylation and alkenylation eluded our efforts despite extensive investigation,^13^ we established the C(sp^3^)−H arylation to be a general transformation, as shown in Scheme 1.

**Scheme 1:**
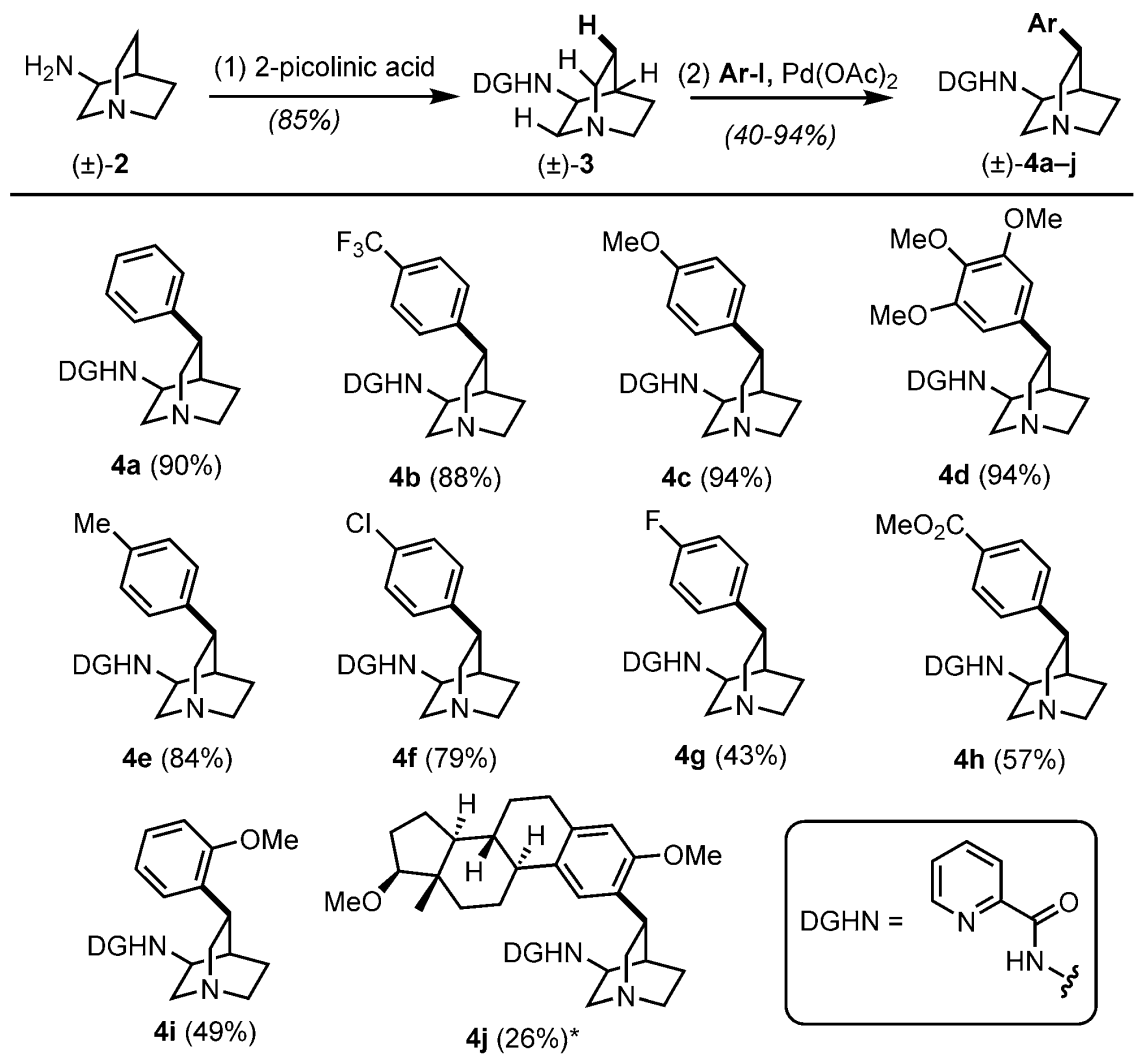
Scope of the divergent C(sp^3^)−H arylation on quinuclidine **3**. Reagents and conditions: (1) picolinic acid, CDI, DMF, RT, 16 h; (2) ArI, Pd(OAc)_2_, Ag_2_CO_3_, DMF, 100 °C, 16 h. * A 1:1 mixture of diastereomers starting from (±)‐**3**.

Thus arenes bearing electron‐donating (Me, OMe) as well as electron‐withdrawing groups (CF_3_, Cl, CO_2_Me, NO_2_) could be efficiently appended to **3**. Notably, 4‐chloroiodobenzene proved to be a suitable partner for the reaction and was converted without affecting the chloride moiety, and esters were also well‐tolerated. *ortho*‐Substituted arenes were introduced, albeit in lower yield. The successful reaction with a complex estradiol derivative is particularly noteworthy (**4 j**).

The availability of intermediate **4 c** in enantiomerically pure (−)‐form (starting from (−)‐**2**) enabled a route towards the natural product (Scheme 2). This was accomplished by ruthenium‐catalyzed oxidative degradation of the anisole moiety to a carboxylic acid,^14^ which was isolated as zwitterion **5**. Formation of Weinreb amide **6** was accomplished by a HATU‐mediated coupling, and subsequent reduction with DIBAL‐H afforded the masked aldehyde **7**.^15^ It is noteworthy that no epimerization at the α‐position to the aldehydic carbon atom was observed during this transformation, likely a fortuitous consequence of hemiaminal formation.

**Scheme 2:**
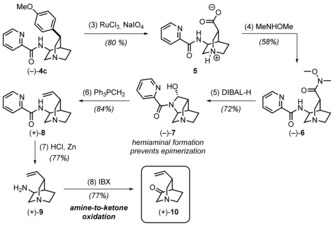
Conversion of arylated (−)‐**4 c** into quinuclidone building block **10**. Reagents and conditions: (3) RuCl_3_, NaIO_4_, H_2_O, EtOAc, CH_3_CN, RT, 18 h; (4) HATU, MeNHOMe**⋅**HCl, Et_3_N, DMF, RT, 18 h; (5) DIBAL‐H, DCM, −78 °C, 1.5 h; (6) Ph_3_PMeBr, LiHMDS, DMSO, THF, −78 °C; (7) Zn, HCl, Zn(OTf)_2_, H_2_O, RT, 1.5 h; (8) IBX, *p*TsOH, CH_3_CN, 70 °C, 2 h.

The desired vinyl moiety was then completed by Wittig olefination to afford alkenyl‐quinuclidine **8** (Scheme 2).^16^ We now sought to remove the directing group and set the stage for an aldol‐type reaction to introduce the quinoline portion of the natural product. Having served its purpose, the picolinamide moiety was removed under reducing conditions, affording free amine **9**.^17^ After extensive experimentation, we found that oxidation of this free amine to the corresponding ketone using IBX led to the desired building block **10** (Scheme 2).^18^ The amine‐to‐ketone functional group interconversion is seldom utilized in total synthesis although several methods have been reported.^19^ In this case, protonation of the nucleophilic quinuclidine nitrogen atom with *p*TsOH as an additive proved to be crucial to achieve oxidation in good yield. In the absence of the acid additive, competing fragmentation of the bicyclic framework took place.

With ketone **10** in hand, the stage was set for the aldol reaction with 6‐methoxyquinoline‐4‐carbaldehyde **12**. We noted that an early attempt toward the synthesis of quinine by an aldol addition between an unsubstituted quinuclidinone and benzaldehyde was previously reported by Stotter and co‐workers more than 30 years ago.^20^ In that study, preliminary experiments with 3‐quinuclidone and benzaldehyde as model substrates revealed rapid epimerization (i.e., configurational instability) of the aldol product. Although the authors overcame this undesired isomerization through in situ reduction of the newly formed addition product, this approach to the synthesis of quinine ultimately proved unsuccessful. More recently, Williams and co‐workers made similar observations in the course of their synthesis of 7‐hydroxyquinine.^21^

In our initial studies, we prepared racemic aryl‐substituted quinuclidone **11 c** (see the Supporting Information for details) as a readily available model substrate. As shown in Scheme 3 a, the aldol reaction of **11** with aldehyde **12** proceeded in very good yield. In analogy to the studies of Stotter,^20^ the product **B** underwent rapid epimerization to the undesired C8 diastereomer upon attempted purification. Recognizing that the acidity at C8 (marked * in Scheme 3 a) must be rapidly curtailed following the aldol reaction, we eventually found that epimerization could be suppressed by in situ treatment of the nascent aldol addition product with Ti(O*i*Pr)_3_Cl followed by conversion of the ketone functional group into a sulfonylhydrazone in a one‐pot operation. This sequence of operations enabled the isolation of **13** in 86 % yield with a high level of diastereoselectivity (>16:1 d.r., relative configuration assigned by X‐ray structure analysis after TBS protection).^22^

**Scheme 3:**
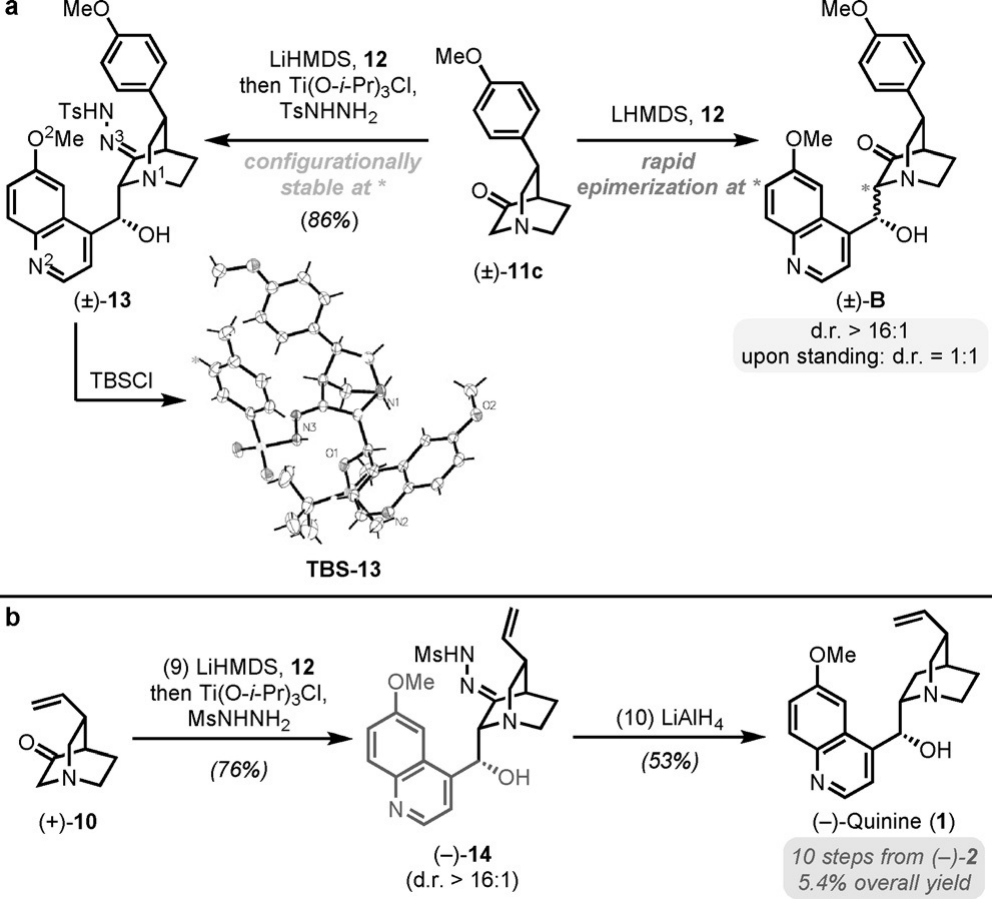
a) Model studies towards a stereoselective aldol reaction. b) Endgame for the total synthesis of (−)‐quinine. Reagents and conditions: (9) LiHMDS, THF, −78 °C, 1 h, then aldehyde **12**, then Ti(O*i*Pr)_3_Cl, MsNHNH_2_, RT, 3 h; (10) LiAlH_4_, MeOH, THF, RT, 10 min.

Applying these same aldol reaction conditions to vinyl‐substituted quinine precursor **10** proved similarly successful; the desired tosylhydrazone was obtained in high yield and excellent diastereoselectivity (see the Supporting Information), leaving the removal of this functional group as the final remaining step. Despite its apparent simplicity, this proved to be a very challenging endeavor. A plethora of common reductants (NaBH_4_, DIBAL‐H, NaBH_3_CN, among others) led either to no reaction or to undesired side products. Various modifications of the Wolff–Kishner reduction (such as Myers or Cram)^23^ were also investigated but failed to afford the desired product, while alternative approaches to trap the configurationally labile aldol product (e.g., as a dithiane or diol) likewise proved unsuccessful. Considerations of molecular models of hydrazone **13** revealed severe steric congestion around the hydrazone C=N carbon atom and suggested that this might be responsible for its experimentally observed slow reduction and sluggish sulfonyl elimination. We eventually found that the critical choice of the less sterically demanding methanesulfonyl‐hydrazone **14** (Scheme 3 b), along with the more reactive reducing system LiAlH_4_/MeOH (presumably generating trimethoxyaluminum hydride) was capable of finally delivering (−)‐quinine (**1**) in 53 % yield (Scheme 3 b). Our synthesis of this natural product thus stands at ten steps from (−)‐**2** and 5.4 % overall yield.

The flexibility of this synthetic approach allowed us to apply an analogous synthetic sequence to prepare the unnatural enantiomer (+)‐quinine. To the best of our knowledge, no study exists on the biological activity of the unnatural antipode of this natural product.^24^ Furthermore, when applied to the C−H activation products (±)‐**4 b** and (±)‐**4 c**, the synthetic sequence outlined herein resulted in two novel analogues of quinine, (±)‐**15 b** and (±)‐**15 c**, in only four additional steps (Table 1; for details, see the Supporting Information). All of these compounds were tested for antimalarial activity in vitro (see the Supporting Information, Table S1). The activity of (+)‐quinine against *Plasmodium falciparum* (*P. falciparum*) is slightly lower than that of (−)‐quinine whereas the novel C3‐aryl analogues (±)‐**15 b** and (±)‐**15 c** showed improved activity. Based on these results, in vivo experiments using the aryl analogues on mice infected with *Plasmodium berghei* (*P*. *berghei*) were performed (Table 1).

**Table 1:** In vivo activity of the racemic aryl analogues (±)‐**15 b** and (±)‐**15 c** and (−)‐quinine hydrochloride as a reference against *P*. *berghei* in mice. 
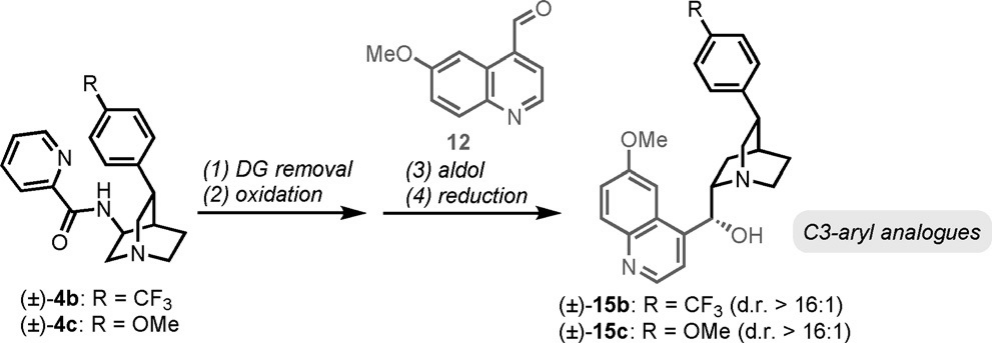

Substance	Dose [mg/kg]	Parasitemia reduction [%]^[a]^	Survival^[b]^ [days]
(−)‐quinine hydrochloride	30	42	euthanized
	100	80	7±0
(±)‐**15 b** ^[c]^	30	98	8±1
	100	99	21±7
(±)‐**15 c** ^[c]^	30	0	euthanized
	100	98	7±1

[a] Blood for parasitemia determination was collected on day 3 (72 h after infection). [b] Mean survival time in days ± standard deviation. Mice with a parasitemia reduction <50 % were euthanized on day 3 post‐infection in order to prevent death, otherwise occurring on day 6. [c] Purity of >99 % determined by HPLC analysis.

The compounds were orally administered in a single dose 24 hours after infection and subsequently both the parasitemia after 3 days and the survival time were recorded. The CF_3_‐aryl analogue (±)‐**15 b** exhibited high antimalarial activity in both doses tested and also significantly increased the survival time of the infected mice whereas (−)‐quinine exhibited only moderate activity at an oral dose of 100 mg kg^−1^. Remarkably, single‐dose administration of 100 mg kg^−1^ of (±)‐**15 b** reduced the amount of parasitized red blood cells to only 1 %, and therefore prolonged the average lifespan of the mice to 21 days, compared to 7 days for (−)‐quinine. Based on these results, replacement of the vinyl group with a more lipophilic aryl moiety seems to increase the efficacy of the drug.

In summary, we have presented a flexible and concise approach to quinine that begins from commercially available 3‐aminoquinuclidine and makes use of the lone amino stereocenter for the introduction of all necessary functionalities of the natural product with excellent regio‐ and stereoselectivity. This enabled the synthesis of both natural (−)‐ and unnatural (+)‐quinine as well as two analogues bearing aromatic groups at the C3 position.^25^ The latter are available in only six synthetic steps from aminoquinuclidine and, given their significantly enhanced antimalarial activity in vitro and in vivo, imply potential therapeutic applications for the chemistry presented herein.

## Conflict of interest

The authors declare no conflict of interest.

## Supporting information

As a service to our authors and readers, this journal provides supporting information supplied by the authors. Such materials are peer reviewed and may be re‐organized for online delivery, but are not copy‐edited or typeset. Technical support issues arising from supporting information (other than missing files) should be addressed to the authors.

Supplementary
