# Supplementary material for: C−H Activation Enables a Concise Total Synthesis of Quinine and Analogues with Enhanced Antimalarial Activity
Source: Angew Chem Int Ed Engl. 2018 Jun 22;57(33):10737–41. doi: 10.1002/anie.201804551 (PMC6146912; doi:10.1002/anie.201804551)
Supplement: Supplementary file 1 — Supplementary [file ANIE-57-10737-s001.pdf]

Angewandte Chemie
Eine Zeitschrift der Gesellschaft Deutscher Chemiker

Supporting Information

C–H Activation Enables a Concise Total Synthesis of Quinine and Analogues with Enhanced Antimalarial Activity

*Daniel H. O' Donovan+, Paul Aillard+, Martin Berger+, Aurélien de la Torre, Desislava Petkova, Christian Knittl-Frank, Danny Geerdink, Marcel Kaiser, and Nuno Maulide**

anie_201804551_sm_miscellaneous_information.pdf

Contents

Materials and Methods S2
General Information S2
Experimental Procedures S3
Synthesis of 4a–j S3
Synthesis of (−)-Quinine from (−)-4c S7
Synthesis of C3-Aryl Analogues (±)-15b and (±)-15c S11
Compounds for Model Studies S16
Summary of Failed Attempts for C−H Functionalization S18
NMR Spectra in Numerical Order S22
Chromatographic Separation of Racemic Quinine S51
Biological Testing S52
Methods S53
X-Ray Analysis S54
Hydrazone **TBS-13** S54

Materials and Methods

General Information

All glassware was oven dried at 100 °C before use. All solvents were distilled from appropriate drying agents prior to use. All reagents were used as received from commercial suppliers unless otherwise stated. Neat infra-red spectra were recorded using a Perkin-Elmer Spectrum 100 FT-IR spectrometer. Wavenumbers ($\tilde{\upsilon} = 1/\lambda$) are reported in cm^{-1}. Mass spectra were obtained using a Finnigan MAT 8200 or (70 eV) or an Agilent 5973 (70 eV) spectrometer, using electrospray ionization (ESI). All ^{1}H-NMR and ^{13}C-NMR spectra were recorded using Bruker AV-400, spectrometers at 300 K. Chemical shifts (δ) are quoted in ppm and coupling constants (J) are quoted in Hz. The resonance of residual $CHCl_3$ for $CDCl_3$ (7.26 ppm for proton spectra and 77.16 ppm for carbon spectra), MeOH for MeOD (3.31 ppm for proton spectra and 49.00 ppm for carbon spectra) and DMSO-d_5 for DMSO-d_6 (2.50 ppm for proton spectra and 39.52 ppm for carbon spectra) were used as internal references. ^{1}H NMR splitting patterns were designated as broad (b), singlet (s), doublet (d), triplet (t), quartet (q) or combinations thereof, splitting patterns that could not be interpreted were designated as multiplet (m). ^{13}C-NMR spectra were recorded using the CPD pulse sequence (compounds **3 · HCl**, **4c**,**d**, **5**, **6**, **7**, **8**, **9**, **10**, **11c**, **13** and **16c**) and the DEPTQ pulse sequence (compounds **1**, **4a**,**b**,**e–j**, **11b**, **14**, **15b**,**c**, **16b** and **17b**,**c**). Reaction progress was monitored by thin layer chromatography (TLC) performed on aluminum plates coated with kieselgel F_{254} with 0.2 mm thickness. Visualization was achieved by a combination of ultraviolet light (254 nm) and acidic potassium permanganate. Flash column chromatography was performed using silica gel 60 (230–400 mesh, Merck and co.). Analytical and preparative HPLC analyses were performed using an Waters-Auto Purification LC/MS System including Waters 2767 Sampler Manager, Waters 2545 Binary Gradient Module, 515 PUMP Waters System Fluidics Organizer SFO, ACQUITY QDa Mass Detector (compact single quad mass detector equipped with an electrospray ionization interface) PC with Waters Masslynx and Fraction Lynx v4.1 Software installed. A Waters 2489 UV/Visible Detector dual wavelength detector was used to acquire UV spectra at 220 nm and 254 nm.

Experimental Procedures

Synthesis of 3

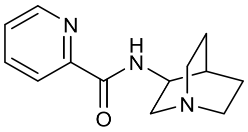

Picolinic acid (9.27 g, 75.3 mmol) was dissolved in dry DMF (300 ml). CDI (12.2 g, 75.3 mmol) was added and the reaction was stirred for 90 min. Then, aminoquinuclidine dihydrochloride (15 g, 75.3 mmol) was added at r.t. and the mixture was stirred at r.t. for 16 h. H_2O (50 ml) was added at 0 ºC followed by 5 M NaOH (100 ml). The reaction mixture was poured into a separating funnel and extracted thrice with dichloromethane. The combined organic phases were washed twice with water, separated and dried over Na_2SO_4 and filtered. Solvents were evaporated *in vacuo* (rotary evaporator). The product was further purified by stirring in MTBE (400 ml) and removal of insoluble impurities by filtration. Solvents were removed *in vacuo* to afford the pure product (14.8 g, 85%) as a thick colorless oil. **^{1}H NMR (500 MHz, D_2O, HCl salt):** δ 8.81 (d, J = 5.3, 1H), 8.48 (td, J = 8.0, 1.5 Hz, 1H), 8.39 (d, J = 8.0 Hz, 1H), 8.03–8.00 (m, 1H), 4.56–4.50 (m, 1H), 3.87 (td, J = 11.8, 3.2 Hz, 1H), 3.49–3.30 (m, 5H), 2.46–2.41 (m, 1H), 2.28–2.20 (m, 1H), 2.14–2.08 (m, 2H), 2.03–1.94 (m, 1H); **^{13}C NMR (125 MHz, D_2O, HCl salt):** δ 163.2 (C), 145.0 (CH), 144.4 (C), 144.3 (CH), 128.9 (CH), 124.4 (CH), 51.5 (CH_2), 46.5 (CH_2) 46.1 (CH_2), 45.6 (CH), 23.8 (CH), 21.1 (CH_2), 16.9 (CH_2); **IR (ATR, neat, cm^{-1}):** 3139, 2920, 2769, 1670, 1603, 1550, 1516, 1454, 1437, 1326, 1299, 1282, 1219, 1092, 1033, 992, 975, 944; **HRMS (ESI)** calcd. for $C_{13}H_{17}N_3ONa$ $[M+Na]^+$: 254.1264, found: 254.1262.
(−)-**3** can be prepared from (−)-3-aminoquinuclidine dihydrochloride in analogy to the procedure for racemic **3** as described above. **$[\alpha]_D^{20}$ = −**48.8° (c = 1.0, $CHCl_3$).

Synthesis of 4a–j

General Procedure for the C−H Arylation Step

To a solution of **3** (1 equiv) and iodoaryl (3 equiv) in DMF (0.3 M) were added successively pivalic acid (1 equiv), $Pd(OAc)_2$ (15 mol%) and Ag_2CO_3 (1 equiv). The resulting mixture was slowly heated to 100 °C and stirred at this temperature for 16 h. After 16 h, the reaction was cooled to room temperature, diluted with 5 M NaOH solution and extracted three times with dichloromethane. The combined organic phase was dried over Na_2SO_4, concentrated and purified by flash chromatography using a gradient of 10 to 30% DMA (CH_2Cl_2/MeOH/NH_4OH 80:20:3) in dichloromethane to afford the pure product.

Synthesis of 4a

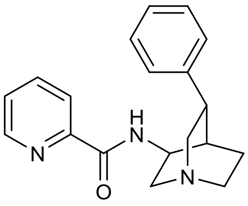

Prepared following the general procedure outlined above using (±)-**3** (100 mg, 0.43 mmol). Purification by column chromatography yielded the pure product as a white solid (119 mg, 0.39 mmol, 90% yield). **^{1}H NMR (600 MHz, $CDCl_3$):** δ 8.16 (d, J = 4.4 Hz, 1H), 7.95 (d, J = 7.7 Hz, 1H), 7.73 (br d, J = 6.9 Hz, 1H), 7.70 (td, J = 7.8, 1.7 Hz, 1H), 7.37 (d, J = 7.8 Hz, 2H), 7.28–7.25 (m, 3H), 7.12 (t, J = 7.4 Hz, 1H), 4.24–4.16 (m, 1H), 3.59–3.39 (m, 3H), 3.19–3.12 (m, 1H), 3.00–2.88 (m, 2H), 2.85–2.77 (m, 1H), 2.62–2.6 (m, 1H), 1.97–1.81 (m, 2H); **^{13}C NMR (150 MHz, $CDCl_3$):** δ 163.7 (C), 149.5 (C), 147.5 (CH), 143.0 (C), 137.0 (CH), 129.1 (CH), 127.1 (CH), 126.0 (CH), 125.8 (CH), 121.7 (CH), 56.9 (CH_2), 51.9 (CH_2), 46.8 (CH), 46.2 (CH_2), 38.2 (CH), 32.7 (CH), 28.8 (CH_2); **IR (ATR, neat, cm^{-1}):** 3349, 2936, 2870, 1666, 1591, 1516, 1462, 1434, 997, 815, 770, 752, 727, 621; **HRMS (ESI)** calcd. for $C_{19}H_{22}N_3O$ $[M+H]^+$: 308.1763, found: 308.1754.

Synthesis of 4b

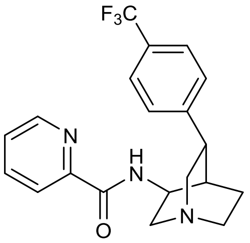

Prepared following the general procedure outlined above using (±)-**3** (50 mg, 0.22 mmol). Purification by column chromatography yielded the pure product as a white solid (71 mg, 0.19 mmol, 88% yield). On a 2.4 mmol scale (555 mg), the product was obtained in 79% yield (713 mg, 1.90 mmol). **^{1}H NMR (600 MHz, $CDCl_3$):** δ 8.13 (d, J = 4.5 Hz, 1H), 7.94 (d, J = 7.7 Hz, 1H), 7.70 (td, J = 7.7, 1.7 Hz, 1H), 7.49 (br d, J = 6.7 Hz, 1H), 7.47–7.4 (m, 4H), 7.28 (ddd, J = 7.5, 4.8, 1.2 Hz, 1H), 4.20–4.15 (m, 1H), 3.53–3.43 (m, 3H), 3.15 (t, J = 9.0 Hz, 1H), 2.97–2.92 (m, 2H), 2.79 (dd, J = 14.4, 4.5 Hz, 1H), 2.72–2.70 (m, 1H), 1.95–1.83 (m, 2H); **^{13}C NMR (150 MHz, $CDCl_3$):** δ 163.7 (C), 149.2 (C), 147.61 (CH), 147.58 (C), 137.1 (CH), 128.2 (q, J_{C-F} = 32.5 Hz, C), 127.3 (CH), 126.1 (CH), 125.7 (q, J_{C-F} = 3.7 Hz, CH), 124.3 (q, J_{C-F} = 271.8 Hz, C), 56.7 (CH_2), 52.0 (CH_2), 46.7 (CH), 46.3 (CH_2), 38.1 (CH), 32.4 (CH), 28.4 (CH_2). **IR (ATR, neat, cm^{-1}):** 2937, 1669, 1517, 1464, 1325, 1163, 1119, 1070, 842, 750, 622, 595; **HRMS (ESI)** calcd. for $C_{20}H_{21}F_3N_3O$ $[M+H]^+$: 376.1637, found: 376.1621.

Synthesis of 4c

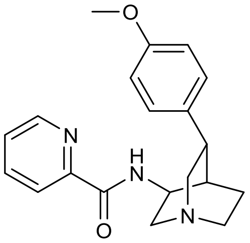

Prepared following the general procedure outlined above using (±)-**3** (50 mg, 0.22 mmol). Purification by column chromatography yielded the pure product as a pale yellow solid (69 mg, 0.21 mmol, 94% yield). On a 2.4 mmol scale (555 mg), the product was obtained in 78% yield (629 mg, 1.86 mmol). **^{1}H NMR (500 MHz, $CDCl_3$):** δ 8.17 (d, J = 4.7 Hz, 1H), 7.96 (d, J = 7.8 Hz, 1H), 7.73 (br d, J = 8.3 Hz, 1H), 7.29–7.26 (m, 3H), 6.82 (d, J = 8.8 Hz, 2H), 4.19–4.13 (m, 1H), 3.52–3.36 (m, 3H), 3.09 (t, J = 8.7 Hz, 1H), 2.95–2.88 (m, 2H), 2.77 (dd, J = 14.2, 5.0 Hz, 1H), 2.52–2.50 (m, 1H), 1.92–1.78 (m, 2H). **^{13}C NMR (125 MHz, $CDCl_3$):** δ 163.6 (C), 157.9 (C), 149.5 (C), 147.4 (CH), 136.9 (CH), 134.9 (C), 127.9 (CH), 125.7 (CH), 121.6 (CH), 114.3 (CH), 56.8 (CH_2), 55.2 (CH_3), 51.9 (CH_2), 46.7 (CH), 46.1 (CH_2), 37.2 (CH), 32.7 (CH), 28.6 (CH_2); **IR (ATR, neat, cm^{-1}):** 3352, 2936, 2870, 1668, 1611, 1590, 1569, 1513, 1463, 1434, 1319, 1284, 1246, 1181, 1036, 997; **HRMS (ESI)** calcd. for $C_{20}H_{25}N_3O_2Na$ $[M+Na]^+$: 360.1682, found: 360.1681.
(−)-**4** can be prepared from (−)-**3** in analogy to the procedure for racemic **4**, as described above. **$[\alpha]_D^{20}$ = −41.0°** (c = 1.0, $CHCl_3$).

Synthesis of 4d

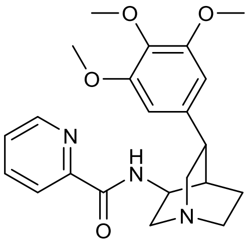

Prepared following the general procedure outlined above using (±)-**3** (100 mg, 0.43 mmol). Purification by column chromatography yielded the pure product as a pale yellow solid (161 mg, 0.40 mmol, 94% yield). **^{1}H NMR (400 MHz, $CDCl_3$):** δ 8.31–8.30 (m, 1H), 7.98–7.95 (m, 2H), 7.70 (td, J = 7.7 Hz, 1.7 Hz, 1H), 7.29–7.26 (m, 1H), 6.56 (s, 2H), 4.21–4.15 (m, 1H), 3.79 (s, 6H), 3.72 (s, 3H), 3.48–3.39 (m, 3H), 3.09–3.04 (m, 1H), 2.92–2.88 (m, 2H), 2.79–2.75 (m, 1H), 2.53–2.51 (m, 1H), 1.90–1.79 (m, 2H); **^{13}C NMR (100 MHz, $CDCl_3$):** δ 163.8, 153.6, 149.5, 147.9, 139.1, 137.0, 136.5, 126.0, 121.6, 104.2, 60.9, 57.2, 56.0, 52.4, 46.7, 46.2, 38.6, 32.9, 28.9; **HRMS (ESI)** calcd. for $C_{22}H_{28}N_3O_4$ $[M+H]^+$: 398.2074, found: 398.2079.

Synthesis of 4e

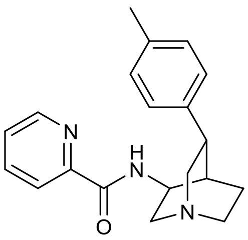

Prepared following the general procedure outlined above using (±)-**3** (50 mg, 0.22 mmol). Purification by column chromatography yielded the pure product as a pale yellow solid (58 mg, 0.18 mmol, 84% yield). **^{1}H NMR (600 MHz, $CDCl_3$):** δ 8.17–8.14 (m, 1H), 7.96 (d, J = 7.8 Hz, 1H), 7.72 (br d, J = 7.4 Hz, 1H), 7.70 (td, J = 7.7, 1.7 Hz, 1H), 7.27 (ddd, J = 7.5, 4.7, 1.2 Hz, 1H), 7.25 (d, J = 8.0 Hz, 2H), 7.08 (d, J = 8.0 Hz, 2H), 4.20–4.16 (m, 1H), 3.54–3.50 (m, 1H), 3.47–3.38 (m, 2H), 3.11 (t, J = 8.7 Hz, 1H), 2.97–2.89 (m, 2H), 2.80 (ddd, J = 14.2, 4.7, 1.7 Hz, 1H), 2.57–2.5 (m, 1H), 2.26 (s, 3H), 1.93–1.87 (m, 1H), 1.86–1.80 (m, 1H); **^{13}C NMR (150 MHz, $CDCl_3$):** δ 163.7 (C), 149.6 (C), 147.5 (CH), 140.0 (C), 137.0 (CH), 135.5 (C), 129.7 (CH), 126.1 (CH), 126.9 (CH), 125.7 (CH), 121.8 (CH), 56.9 (CH_2), 51.9 (CH_2), 46.8 (CH), 46.3 (CH_2), 37.8 (CH), 32.7 (CH), 28.8 (CH_2), 21.0 (CH_3); **IR (ATR, neat, cm^{-1}):** 3352, 2939, 2870, 1667, 1515, 1461, 1434, 997, 824, 801, 751, 621; **HRMS (ESI)** calcd. for $C_{20}H_{24}N_3O$ $[M+H]^+$: 322.1919, found: 322.1911.

Synthesis of 4f

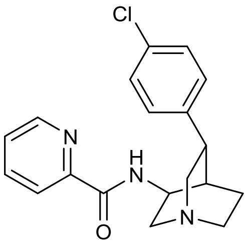

Prepared following the general procedure outlined above using (±)-**3** (50 mg, 0.22 mmol). Purification by column chromatography yielded the pure product as yellow oil (58 mg, 0.17 mmol, 79% yield). **^{1}H NMR (600 MHz, $CDCl_3$):** δ 8.28–8.27 (m, 1H), 7.96 (d, J = 7.8 Hz, 1H), 7.73 (td, J = 7.7, 1.7 Hz, 1H), 7.61 (br d, J = 7.5 Hz, 1H), 7.31 (ddd, J = 7.6, 4.7, 1.2 Hz, 1H), 7.29 (d, J = 8.9 Hz, 2H), 7.22 (d, J = 8.5 Hz, 1H), 4.19–4.15 (m, 1H), 3.50–3.40 (m, 3H), 3.10 (t, J = 8.7 Hz, 1H), 2.96–2.90 (m, 2H), 2.77 (dd, J = 14.1, 4.7 Hz, 1H), 2.59–2.57 (m, 1H), 1.93–1.81 (m, 2H); **^{13}C NMR (150 MHz, $CDCl_3$):** δ 163.4 (C), 149.3 (C), 147.9 (CH), 141.8 (C), 137.1 (CH), 131.9 (C), 129.1 (CH), 128.4 (CH), 126.0 (CH), 121.7 (CH), 56.8 (CH_2), 51.9 (CH_2), 46.7 (CH), 46.3 (CH_2), 37.6 (CH), 32.7 (CH), 28.6 (CH_2); **IR (ATR, neat, cm^{-1}):** 3356, 2936, 2870, 1665, 1513, 1462, 1434, 1091, 1011, 997, 831, 750, 621; **HRMS (ESI)** calcd. for $C_{19}H_{21}ClN_3O$ $[M+H]^+$: 342.1373, found: 342.1366.

Synthesis of 4g

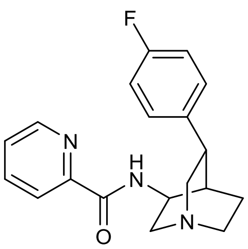

Prepared following the general procedure outlined above using (±)-**3** (50 mg, 0.22 mmol). Purification by column chromatography yielded the pure product as white solid (30 mg, 0.09 mmol, 43% yield). **^{1}H NMR (600 MHz, $CDCl_3$):** δ 8.23–8.22 (m, 1H), 7.97 (d, J = 7.8 Hz, 1H), 7.72 (td, J = 7.7, 1.7 Hz, 1H), 7.64 (br d, J = 7.3 Hz, 1H), 7.34–7.29 (m, 3H), 6.97–6.94 (m, 2H), 4.20–4.16 (m, 1H), 3.51–3.41 (m, 3H), 3.11 (t, J = 8.8 Hz, 1H), 2.96–2.90 (m, 2H), 2.77 (dd, J = 14.2, 4.5 Hz, 1H), 2.57–2.55 (m, 1H), 1.93–1.82 (m, 2H); **^{13}C NMR (150 MHz, $CDCl_3$):** δ 163.7 (C), 161.5 (d, J_{C-F} = 244.8 Hz, CH), 149.4 (C), 147.7 (CH), 137.1 (CH), 138.8 (d, J_{C-F} = 3.4 Hz, C), 128.6 (d, J_{C-F} = 8.0 Hz, CH), 126.0 (CH), 121.7 (CH), 115.7 (d, J_{C-F} = 21.2 Hz, CH), 56.9 (CH_2), 52.1 (CH_2), 46.7 (CH), 46.3 (CH_2), 37.5 (CH), 32.8 (CH), 28.7 (CH_2); **IR (ATR, neat, cm^{-1}):** 3354, 2936, 2873, 1664, 1592, 1569, 1512, 1463, 1435, 1223, 1163, 997, 839, 813, 751, 687, 621; **HRMS (ESI)** calcd. for $C_{19}H_{21}FN_3O$ $[M+H]^+$: 326.1669, found: 326.1670.

Synthesis of 4h

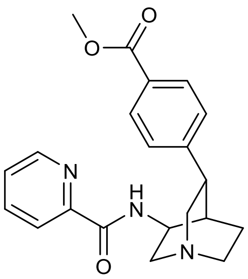

Prepared following the general procedure outlined above using (±)-**3** (50 mg, 0.22 mmol). Purification by column chromatography yielded the pure product as yellow oil (45 mg, 0.12 mmol, 57% yield). **^{1}H NMR (600 MHz, CDCl$_3$):** δ 8.10–8.09 (m, 1H), 7.93–7.91 (m, 1H), 7.88 (d, J = 8.4 Hz, 2H), 7.68 (td, J = 7.6, 1.7 Hz, 1H), 7.52 (br d, J = 7.8 Hz, 1H), 7.42 (d, J = 8.1 Hz, 2H), 7.23 (ddd, J = 7.6, 4.8, 1.1 Hz, 1H), 4.19-4.15 (m, 1H), 3.89 (s, 3H), 3.57–3.53 (m, 1H), 3.47–3.43 (m, 2H), 3.17 (t, J = 8.7 Hz, 1H), 2.97–2.91 (m, 2H), 2.79 (dd, J = 14.7, 4.5 Hz, 1H), 2.68-2.66 (m, 1H), 1.94–1.90 (m, 1H), 1.88-1.82 (m, 1H); **^{13}C NMR (150 MHz, CDCl$_3$):** δ 167.0 (C), 163.7 (C), 149.3 (C), 148.9 (C), 147.6 (CH), 137.0 (CH), 130.3 (CH), 127.8 (C), 127.0 (CH), 125.9 (CH), 121.7 (CH), 56.7 (CH$_2$), 52.1 (CH$_3$), 51.8 (CH$_2$), 46.7 (CH), 46.3 (CH$_2$), 38.4 (CH), 32.8 (CH), 28.5 (CH$_2$); **IR (ATR, neat, cm^{-1}):** 3359, 2948, 1717, 1669, 1609, 1517, 1435, 1282, 1187, 1110, 776, 743, 622; **HRMS (ESI)** calcd. for $C_{21}H_{24}N_3O_3$ $[M+H]^+$: 366.1818, found: 366.1809.

Synthesis of 4i

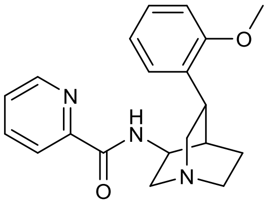

Prepared following the general procedure outlined above using (±)-**3** (50 mg, 0.22 mmol). Purification by column chromatography yielded the pure product as pale yellow solid (36 mg, 0.11 mmol, 49% yield). **^{1}H NMR (600 MHz, CDCl$_3$):** δ 8.24 (d, J = 4.7 Hz, 1H), 7.97 (d, J = 7.8 Hz, 1H), 7.96 (br d, J = 8.0 Hz, 1H), 7.71 (td, J = 7.8, 1.8 Hz, 1H), 7.48 (d, J = 7.6 Hz, 1H), 7.30 (ddd, J = 7.5, 4.7, 1.2 Hz, 1H), 7.22 (td, J = 8.0, 1.5 Hz, 1H), 6.98 (td, J = 7.6, 1.0 Hz, 1H), 6.86 (d, J = 8.1 Hz, 1H), 4.22–4.17 (m, 1H), 3.82 (s, 3H), 3.61 (ddd, J = 13.8, 7.6, 2.0 Hz, 1H), 3.54–3.49 (m, 1H), 3.47 (t, J = 9.3 Hz, 1H), 3.32–3.28 (m, 1H), 3.06–2.91 (m, 3H), 2.53–2.50 (m, 1H), 2.00–1.95 (m, 1H), 1.84–1.79 (m, 1H). **^{13}C NMR (150 MHz, CDCl$_3$):** δ 163.8 (C), 157.8 (C), 149.6 (C), 147.6 (CH), 137.1 (CH), 130.4 (C), 127.4 (CH), 126.6 (CH), 125.19 (CH), 121.8 (CH), 121.0 (CH), 111.0 (CH), 56.6 (CH$_2$), 55.4 (CH$_3$), 50.8 (CH$_2$), 46.7 (CH), 46.3 (CH$_2$), 33.3 (CH), 30.8 (CH), 28.7 (CH$_2$). **IR (ATR, neat, cm^{-1}):** 3355, 2939, 1667, 1591, 1516, 1462, 1436, 1290, 1242, 1028, 997, 752, 620; **HRMS (ESI)** calcd. for $C_{20}H_{24}N_3O_2$ $[M+H]^+$: 338.1869, found: 338.1862.

Synthesis of 4j

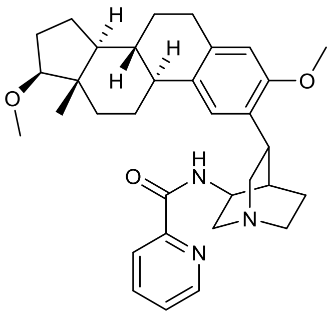

Prepared using a modification of the general procedure outlined above using (±)-**3** (50 mg, 0.22 mmol) and Pd(OAc)$_2$ (0.15 mg, 0.06 mmol). Purification by column chromatography yielded the product as a white solid (30 mg, 0.09 mmol, 26% yield, 1:1 mixture of diastereoisomers). **^{1}H NMR (700 MHz, CDCl$_3$):** δ 8.27 (d, J = 4.6 Hz, 1H), δ 8.25 (d, J = 4.6 Hz, 1H), 8.04 (d, J = 7.8 Hz, 1H), 8.01 (d, J = 7.8 Hz, 1H), 7.88 (br d, J = 7.7 Hz, 1H), 7.84 (br d, J = 7.7 Hz, 1H), 7.75 (td, J = 7.6, 1.6 Hz, 2H), 7.34–7.27 (m, 4H), 6.62 (s, 1H), 5.57 (s, 1H), 4.27–4.22 (m, 2H), 3.81 (s, 3H), 3.79 (s, 3H), 3.65–3.54 (m, 4H), 3.48–3.43 (m, 2H), 3.41–3.35 (m, 2H), 3.36 (s, 3H), 3.32 (s, 3H), 3.29 (t, J = 8.5 Hz, 1H), 3.24 (t, J = 8.5 Hz, 1H), 3.13–2.97 (m, 6H), 2.89–2.77 (m, 4H), 2.61–2.56 (m, 2H), 3.27–2.22 (m, 1H), 2.13–1.98 (m, 8H), 1.94–1.81 (m, 5H), 1.72–1.63 (m, 2H), 1.54–1.43 (m, 2H), 1.42–1.21 (m, 11H), 1.16–1.09 (m, 2H), 1.04–0.98 (m, 1H), 0.73 (s, 3H), 0.46 (s, 3H); **^{13}C NMR (176 MHz, CDCl$_3$):** δ 164.0 (C), 163.8 (C), 155.53 (C), 155.47 (C), 149.8 (C), 149.7 (C), 147.8 (CH), 147.7 (CH), 137.23 (CH), 137.19 (CH),

136.2 (C), 136.0 (C), 132.5 (C), 132.2 (C), 126.8 (bs, C), 126.1 (CH), 126.0 (CH), 124.2 (CH), 123.4 (CH), 122.2 (CH), 122.1 (CH), 111.51 (CH), 111.49 (CH), 90.8 (CH), 90.7 (CH), 58.1 (CH_3), 58.0 (CH_3), 56.2 (CH_2), 56.1 (CH_2), 55.49 (CH_3), 55.48 (CH_3), 51.3 (CH_2), 50.8 (CH_2), 50.6 (CH), 50.3 (CH), 46.46 (CH), 46.43 (CH), 46.3 (CH_2), 46.2 (CH_2), 44.3 (CH), 44.1 (CH), 43.34 (CH_2), 43.31 (CH_2), 39.1 (CH), 38.7 (CH), 38.2 (CH_2), 38.0 (CH_2), 33.5 (CH), 33.0 (CH), 30.6 (CH), 30.1 (CH_2), 29.6 (CH_2), 27.93 (CH_2), 27.92 (CH_2), 27.7 (CH_2), 27.3 (CH_2), 26.6 (CH_2), 26.5 (CH_2), 23.20 (CH_2), 23.15 (CH_2), 11.7 (CH_3); **IR (ATR, neat, cm^{-1}):** 3349, 2925, 1666, 1506, 1462, 1434, 1382, 1358, 1287, 1236, 1205, 1117, 10102, 1029, 996, 730, 698, 619; **HRMS (ESI)** calcd. for $C_{33}H_{44}N_3O_3$ $[M+H]^+$: 530.3383, found: 530.3374.

Synthesis of (−)-Quinine from (−)-4c

Synthesis of 5

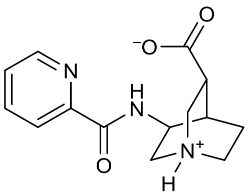

A 250 ml round bottomed flask was charged with (−)-**4** (1.35 g, 4.00 mmol), ruthenium(III) chloride (331 mg, 1.60 mmol) and sodium periodate (20.55 g, 96.03 mmol) to which H_2O was added (53 ml) followed by EtOAc (13.5 ml) and acetonitrile (13.5 ml). The flask was sealed with a septum pierced with a cannula to allow gases to escape and the reaction was stirred at room temperature overnight. After 18 h, the reaction was filtered through celite, and the pad was washed with 200 ml H_2O. The filtrate was transferred to a separating funnel and washed three times with DCM (3 × 50 ml). The halogenated phases were discarded, while the aqueous phase was collected and evaporated to a volume of *ca.* 20 ml *in vacuo*. To this residue was added 10 ml of 1 M HCl and the mixture was loaded onto a wet column of DOWEX–50WX8 (20 g) which had been pre-washed with 150 ml water. The column was washed with 4 × 500 ml portions of H_2O, during which time the $NaIO_4$ eluted. The column was then washed with 5 × 250 ml portions of 1 M NH_4OH; the product eluted in the first four fractions. Water was evaporated from the collected fractions *in vacuo* followed by drying under high vacuum to afford the zwitterionic intermediate **5** as a dark red solid (0.88 g, 80%). **^{1}H NMR (500 MHz, D_2O):** δ 8.65 (br s, 1H), 8.00 (m, 2H), 7.62 (dd, J = 4.4, 4.3 Hz, 1H), 4.54–4.46 (m, 1H), 3.87–3.74 (m, 2H), 3.57 (ddd, J = 10.9, 2.6, 2.6 Hz, 1H), 3.39–3.32 (m, 3H), 2.96 (t, J = 8.6 Hz), 2.87–2.84 (m, 1H), 2.17–2.08 (m, 2H); **^{13}C NMR (125 MHz, D_2O):** δ 178.5 (C), 166.5 (C), 148.9 (CH), 148.0 (C), 138.2 (CH), 127.3 (CH), 122.3 (CH), 52.6 (CH_2), 49.5 (CH_2), 42.5 (CH_2), 44.5 (CH), 39.0 (CH), 27.5 (CH), 22.8 (CH_2); **IR (ATR, neat, cm^{-1}):** 3240, 3059, 3015, 1655, 1586, 1568, 1509, 1464, 1434, 1376, 1292, 1088, 1043, 996, 911; **HRMS (ESI)** calcd. for $C_{14}H_{18}N_3O_3$ $[M+H]^+$: 276.1343, found: 276.1341.

Synthesis of 6

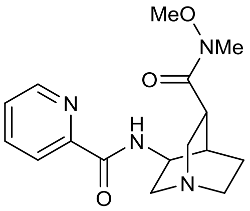

To a suspension of **5** (1.08 g, 3.92 mmol) in 30 ml DMF was added triethylamine (1.72 ml, 12.35 mmol) followed by *N*,*O*-dimethylhydroxylamine hydrochloride (401 mg, 4.11 mmol) and HATU (1.56 g, 4.11 mmol). The reaction was stirred overnight at room temperature. After 18 h, the reaction mixture was poured into 250 ml DCM to which 300 ml 1:1 5 M NaOH/brine was added, and the mixture was extracted three times with 250 ml DCM. The combined organic phase was dried over Na_2SO_4, filtered, and evaporated *in vacuo*. The crude material was purified by chromatography over silica gel using a gradient of 10 to 30% DMA (CH_2Cl_2/MeOH/NH_4OH 80:20:3) in DCM, followed by evaporation of solvents to afford the Weinreb amide **6** as a pale pink gum (727 mg, 58%). **^{1}H NMR (500 MHz, CD_2Cl_2):** δ 8.85 (br d, J = 6.2 Hz, 1H), 8.61 (d, J = 4.8 Hz), 8.08 (dt, J = 7.8, 1.1 Hz, 1H), 7.81 (td, J = 7.8, 1.6 Hz, 1H), 7.39 (ddd, J = 7.8, 4.8, 1.6 Hz, 1H),

4.21–4.10 (m, 1H), 3.63 (s, 3H), 3.44 (dd, J = 12.6, 4.5 Hz, 1H), 3.30 (t, J = 12.5 Hz, 1H), 3.04 (s, 3H), 3.01–2.71 (m, 5H), 2.36–2.30 (m, 1H), 1.84–1.62 (m, 2H); **^{13}C NMR (125 MHz, CD_2Cl_2):** δ 177.0 (C), 164.2 (C), 150.8 (C), 148.6 (CH), 137.4 (CH), 126.3 (CH), 122.3 (CH), 61.8 (CH_3), 55.7 (CH_2), 50.5 (CH_2), 46.4 (CH_2), 46.0 (CH), 37.4 (CH), 32.6 (CH_3), 30.4 (CH), 28.7 (CH_2); **IR (ATR, neat, cm^{-1}):** 3355, 2938, 2871, 1668, 1590, 1568, 1517, 1465, 1435, 1385, 1327, 1173, 1086, 1042, 997; **HRMS (ESI)** calcd. for $C_{16}H_{22}N_4O_3Na$ [M+Na]$^+$: 341.1584, found: 341.1586; **$[\alpha]_D^{20}$ = −**28.2° (c = 1.0, $CHCl_3$).

Synthesis of 7

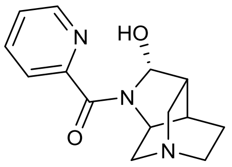

To a solution of **6** (550 mg, 1.73 mmol) in 11.5 ml DCM under argon at −78 °C was slowly added DIBAL-H as a 1 M solution in DCM (10.38 ml, 10.58 mmol) in one portion with stirring. The reaction was stirred at −78 °C for 1.5 h before quenching the excess DIBAL-H with 15 ml EtOAc. The cooling bath was then removed and 20 ml saturated Rochelle's salt solution in H_2O was added neat. 5 ml $CHCl_3$ and 5 ml H_2O were then added, and the reaction was stirred vigorously at room temperature for 1 h. The mixture was then transferred to a separating funnel and extracted from 150 ml 2 M NaOH/brine using three volumes of DCM (3 × 100 ml). The organic phase was dried over Na_2SO_4, filtered, and evaporated *in vacuo* to afford the product **7** as a dark green waxy solid (purity >90%, 357 mg, 72%) which could be used without further purification. **^{1}H NMR (500 MHz, $CDCl_3$):** δ 8.56 (d, J = 4.9 Hz, 1H), 8.16 (d, 1H, J = 7.8 Hz), 7.95 (td, J = 7.8, 1.5 Hz, 1H), 7.50 (ddd, J = 7.8, 4.9, 1.5 Hz, 1H), 7.18 (s, 1H), 5.11 (s, 1H), 4.36 (t, J = 6.0 Hz, 1H), 3.21–3.08 (m, 2H), 3.06–2.96 (m, 2H), 2.91–2.80 (m, 2H), 2.62 (br d, J = 14.0 Hz, 1H), 2.39 (d, J = 9.0 Hz, 1H), 1.91–1.83 (m, 2H); **^{13}C NMR (125 MHz, $CDCl_3$):** δ 165.8 (C), 152.6 (C), 147.1 (CH), 138.2 (CH), 126.1 (CH), 126.0 (CH), 87.8 (CH), 56.3 (CH), 55.9 (CH_2), 52.1 (CH_2), 48.8 (CH_2), 41.1 (CH), 27.7 (CH), 16.4 (CH_2); **IR (ATR, neat, cm^{-1}):** 3375, 3057, 2954, 2934, 2871, 1668, 1623, 1584, 1568, 1515, 1451, 1412, 1384, 1354, 1346, 1306, 1255, 1222, 1189, 1147, 1114, 1101, 1081, 1049, 998; **HRMS (ESI)** calcd. for $C_{14}H_{17}N_3O_2Na$ [M+H]$^+$: 282.1213, found: 282.1214; **$[\alpha]_D^{20}$ = −**11.3° (c = 1, $CHCl_3$).
The stereochemistry of **7** was determined *via* $^3J_{HH}$-coupling. The signal of the hemiaminal (CH) at 5.11 ppm, which appears as singulett, with a very small coupling constant corresponds, according to the Karplus equation, to a dihedral angle of approximately 90 degrees and, therefore, the shown diastereomer.

Synthesis of 8

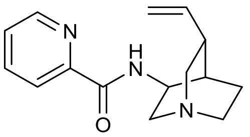

Methyltriphenylphosphonium bromide (165 mg, 0.463 mmol) was placed in a dry argon-filled schlenk and evaporated for 5 min at 3 × 10^{-1} mbar, then filled again with argon. To this flask, 380 µl THF was added and the flask was cooled to −78 °C, followed by addition of LiHMDS as a 1 M solution in THF (425 µl, 0.425 mmol). The solution was allowed to come to room temperature and stirred for 20 min to form the bright yellow ylid solution. Hemiaminal **7** (purity >90%, 30 mg, 0.105 mmol) was placed in a separate argon-filled schlenk, which was evaporated at 3 × 10^{-1} mbar for 5 min and refilled with argon. 60 µl dry DMSO was added and the rxn was stirred for 5 min, followed by the addition of 570 µl THF. The reaction was cooled to −78 °C and 650 µl of the freshly prepared ylid solution was introduced via syringe. The reaction was stirred at room temperature for 45 min and then quenched at 0 °C using 3 ml H_2O. The reaction mixture was then extracted using 1:1 2 M NaOH/brine and three volumes of DCM. The organic phase was dried over Na_2SO_4, then filtered and evaporated *in vacuo*. The crude residue was purified by chromatography over silica gel using a gradient of 15 to 30% DMA (CH_2Cl_2/MeOH/NH_4OH 80:20:3) in dichloromethane to afford the pure product as a yellow oil

(22 mg, 84%). On a 1.39 mmol scale (purity >90%, 400 mg), the product was obtained in 56% yield (200 mg, 0.78 mmol). **^{1}H NMR (500 MHz, $CDCl_3$):** δ 8.53 (d, J = 4.9 Hz, 1H), 8.43 (br d, J = 7.0 Hz, 1H), 8.15 (d, J = 7.7 Hz, 1H), 7.82 (td, J = 7.7, 1.7 Hz, 1H), 7.50 (ddd, J = 7.7, 4.9, 1.7 Hz, 1H), 6.06 (ddd, J = 17.2, 10.2, 5.6 Hz, 1H), 5.20–5.13 (m, 2H), 4.24–4.17 (m, 1H), 3.43 (ddd, J = 14.1, 9.7, 2.2 Hz, 1H), 3.21 (ddd, J = 13.5, 10.2, 2.3 Hz, 1H), 2.99 (dd, J = 13.5, 7.0 Hz, 1H), 2.83 (t, J = 8.0 Hz, 2H), 2.67 (dd, J = 14.1, 4.8 Hz, 1H), 2.48–2.42 (m, 1H), 2.17–2.13 (m, 1H), 1.75–1.70 (m, 2H); **^{13}C NMR (125 MHz, $CDCl_3$):** δ 163.7 (C), 149.9 (C), 148.1 (CH), 142.3 (CH), 137.2 (CH), 126.0 (CH), 122.1 (CH), 114.5 (CH_2), 56.8 (CH_2), 52.7 (CH_2), 46.7 (CH), 46.0 (CH_2), 37.6 (CH), 32.4 (CH), 28.2 (CH_2); **IR (ATR, neat, cm^{-1}):** 3373, 3058, 2934, 2865, 1667, 1590, 1569, 1513, 1464, 1434, 1321, 1290, 1243, 1168, 1087, 1062, 1042, 997; **HRMS (ESI)** calcd. for $C_{15}H_{19}N_3ONa$ [M+Na]$^+$: 280.1420, found: 280.1418; **[α]$_D^{20}$** = +9.4° (c = 1.0, $CHCl_3$).

Synthesis of 9

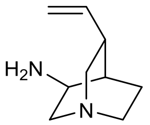

8 (85 mg, 0.33 mmol) was suspended in 6 ml H_2O to which conc. HCl was added (0.23 ml), followed by $Zn(OTf)_2$ (120 mg, 0.33 mmol). After stirring at room temperature for 5 min, Zn dust (324 mg, 4.95 mmol) was added. After 1.5 h, the reaction mixture was filtered through celite and the pad was washed with 50 ml H_2O. The filtrate was cooled in an ice-water bath and 40 ml DCM was added with stirring, followed by slow addition of 10 M aq. NaOH (20 ml). After 15 min, the mixture was poured into a separating funnel and extracted three times DCM (3 × 60 ml). The combined organic phase was dried over Na_2SO_4, filtered, and evaporated at 150 mbar/35 °C. The crude material was purified by chromatography over silica gel using a gradient of 30 to 50% DMA (CH_2Cl_2/MeOH/NH_4OH 80:20:3) in dichloromethane. After chromatography, solvents were removed under the same conditions as before, at 150 mbar/35 °C. The purified amine (clear oil, purity >90%, 43 mg, 77%) was stored under argon at −78 °C (in a sealed vessel immersed in dry ice) and used in the subsequent reaction within 36 h to minimize decomposition. On a 1.28 mmol scale (330 mg), the product was obtained in 35% yield (purity >90%, 75 mg, 0.44 mmol). Concerning the chemical instability of **9**, upscaling is not recommended as bigger scale setups require more time for work-up and purification. **^{1}H NMR (500 MHz, CD_2Cl_2):** δ 6.24 (ddd, J = 17.3, 10.4, 6.4 Hz, 1H), 4.99 (d, J = 17.3 Hz, 1H), 4.93 (d, J = 10.4 Hz, 1H), 3.11 (ddd, J = 13.2, 9.4, 2.2 Hz, 1H), 3.05 (ddd, J = 13.2, 10.2, 2.2 Hz, 1H), 2.95–2.91 (m, 1H), 2.79 (ddd, J = 13.2, 7.0, 1.9 Hz, 1H), 2.73–2.60 (m, 2H), 2.39–2.29 (m, 2H), 1.79–1.75 (m, 1H), 1.66–1.42 (m, 4H); **^{13}C NMR (125 MHz, CD_2Cl_2):** δ 150.0 (CH), 112.7 (CH_2), 59.9 (CH_2), 53.9 (CH_2), 50.4 (CH_2), 46.1 (CH), 39.7 (CH), 36.5 (CH), 29.3 (CH_2); **IR (ATR, neat, cm^{-1}):** 3274, 3072, 2931, 2867, 1633, 1592, 1454, 1320, 1265, 1069, 1048, 998, 974, 907, 814, 732; **HRMS (ESI)** calcd. for $C_9H_{16}N_2$ [M]$^+$: 152.1313, found: 152.1312; **[α]$_D^{20}$** = +68.8° (c = 1.0, $CHCl_3$).

Synthesis of 10

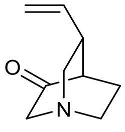

p-Toluenesulfonic acid monohydrate (137 mg, 0.72 mmol) was added to a solution of **9** (purity >90%, 110 mg, 0.65 mmol) in MeCN (7 ml) at r.t. The reaction mixture was stirred at this temperature for 10 min then IBX (223 mg, 0.80 mmol) was added and the reaction was stirred at 70 °C for 2 h. The reaction was cooled to room and then quenched by a 2 M NaOH solution. The mixture was poured into a separating funnel and extracted three times with dichloromethane (3 × 60 ml). The combined organic phase was dried over Na_2SO_4, filtered, and evaporated at 150 mbar/35 °C. The crude material was purified by chromatography over silica gel using a gradient of 1 to 4% MeOH in dichloromethane to afford the desired ketone as clear oil (75 mg, 77%). **^{1}H NMR (400 MHz, CD_2Cl_2):** δ 5.65 (ddd, J =

17.2, 10.3, 7.6, 1H), 4.98 (ddd, J = 17.2, 1.4, 1.4 Hz, 1H), 4.96 (ddd, J = 10.3, 1.4, 1.4 Hz, 1H), 3.30–3.08 (m, 3H), 2.99–2.78 (m, 3H), 2.62 (ddd, J = 13.8, 6.1, 2.2 Hz, 1H), 2.39 (q, J = 3.0 Hz, 1H), 2.09–1.93 (m, 2H); **^{13}C NMR (100 MHz, CD_2Cl_2):** δ 218.7 (C), 141.2 (CH), 115.3 (CH_2), 63.7 (CH_2), 54.5 (CH_2), 46.7 (CH_2), 46.2 (CH), 43.2 (CH_2), 26.3 (CH_2); **IR (ATR, neat, cm^{-1}):** 2943, 2873, 1729, 1639, 1453, 1404, 1340, 1322, 1304, 1262, 1228, 1134, 1068, 1045, 991, 968, 918, 872, 812; **HRMS (ESI)** calcd. for $C_9H_{16}N_2$ $[M+H]^+$: 152.1070, found: 152.1070. **$[\alpha]_D^{20}$** = +54.7° (c = 1.0, $CHCl_3$).

Synthesis of 14

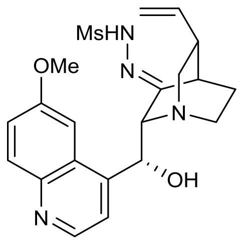

10 (50 mg, 0.33 mmol) was dissolved in 3 ml of anhydrous THF under argon and cooled to 0 °C, followed by addition of LiHMDS (1.0 M in THF, 347 µl, 0.35 mmol). The reaction was stirred for 30 min at 0 °C, then cooled to −78 °C and 6-methoxyquinoline–4-carbaldehyde **12** (68 mg, 0.36 mmol) was added neat. After 1 h at −78 °C, Ti(O-i-Pr)$_3$Cl (202 mg, 0.78 mmol) was added and the reaction was brought to 0 °C and stirred for 5 min, followed by addition of methanesulfonyl hydrazide (73 mg, 0.66 mmol). After 5 min at 0 °C, the reaction was brought to room temperature and stirred for 3h, then quenched at 0 °C using 1.0 ml sat. aq. $NaHCO_3$. The mixture was extracted from sat. aq. $NaHCO_3$ (30 ml) with DCM (3 × 30 ml). The combined organic phase was dried over Na_2SO_4 and filtered. The crude was purified by chromatography using a gradient of 10 to 30% DMA (CH_2Cl_2/MeOH/NH_4OH 80:20:3) in dichloromethane to afford the product as a white solid (108 mg, 76%, dr > 16:1). **^{1}H NMR (600 MHz, $CDCl_3$):** δ 8.24 (d, J = 4.6 Hz, 1H), 7.77 (d, J = 9.2 Hz, 1H), 7.59 (d, J = 2.5 Hz, 1H), 7.28 (dd, J = 9.2, 2.8 Hz, 1H), 7.20 (d, J = 4.6 Hz, 1H), 5.70–5.65 (m, 1H), 5.44 (d, J = 9.7 Hz, 1H), 4.97–4.95 (m, 1H), 4.94 (br s, 1H), 4.23 (d, J = 9.5 Hz, 1H), 3.91 (s, 3H), 3.08 (s, 3H), 3.03–2.91 (m, 2H), 2.66–2.63 (m, 1H), 2.60–2.56 (m, 1H), 2.52–2.47 (m, 1H), 2.19–2.15 (m, 1H), 2.05–1.99 (m, 1H), 1.89–1.84 (m, 1H); **^{13}C NMR (150 MHz, $CDCl_3$):** 161.8 (C), 158.2 (C), 146.8 (CH), 145.0 (C), 144.3 (C), 139.5 (CH), 131.1 (CH), 126.9 (C), 122.4 (CH), 120.1 (CH), 115.2 (CH_2), 101.9 (CH), 73.1 (CH), 66.1 (CH), 56.2 (CH_2), 56.0 (CH_3), 45.0 (CH), 41.0 (CH_2), 38.7 (CH), 38.4 (CH_3), 23.4 (CH_2); **IR (ATR, neat, cm^{-1}):** 2933, 1739, 1509, 1473, 1432, 1323, 1241, 1158, 1087, 992, 849, 734; **HRMS (ESI)** calcd. for $C_{21}H_{27}N_4O_4S$ $[M+H]^+$: 431.1753, found: 431.1744. **$[\alpha]_D^{20}$ = −**20.4° (c = 0.5, $CHCl_3$).

Synthesis of Quinine (1)

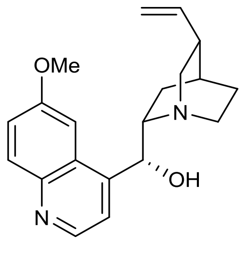

Lithium aluminium hydride solution (1.0 M in THF, 116 µl, 0.116 mmol) was diluted with THF (0.3 ml) in a Schlenk flask under argon. Methanol (14 µl, 0.348 mmol) was added dropwise at 0°C and stirred at this temperature for 10 min. In another Schlenk flask, a solution of hydrazone **14** (10 mg, 0.023 mmol) was diluted in THF (0.3 ml). The aluminium hydride solution was added dropwise to the solution of hydrazone at 0°C. The reaction mixture was then warmed to room temperature and stirred for an additionnal 10 min before being quenched by sat. aq. $NaHCO_3$ solution at 0°C. The mixture was extracted from sat. aq. $NaHCO_3$ with three volumes of DCM. The combined organic phase was dried over Na_2SO_4 and filtered. The crude was purified by chromatography using a gradient of 10 to 30% DMA (CH_2Cl_2/MeOH/NH_4OH 80:20:3) in dichloromethane to afford the product as a white powder (4 mg, 53%). Data in accordance with the literature.[1] **^{1}H NMR (400 MHz, DMSO-d$_6$):** 8.67 (d, J = 4.5 Hz, 1H), 7.92 (d, J = 9.2 Hz, 1H), 7.49–7.52 (m, 2H), 7.39 (dd, J = 9.2, 2.8 Hz, 1H), 5.86 (dd, J = 17.5, 10.3, 7.6 Hz, 1H), 5.65 (br d, J = 4.7 Hz, 1H), 5.28–5.23 (m, 1H), 5.02–4.91 (m, 2H), 3.90 (s, 3H), 3.26–3.17 (m, 1H), 3.07 (q, J = 7.6 Hz, 1H), 2.93–

2.84 (m, 1H), 2.48–2.40 (m, 1H), 2.25–2.17 (m, 1H), 1.77–1.60 (m, 4H), 1.49–1.38 (m, 1H). **$[\alpha]_D^{20}$** = −151.8° (c = 0.4, EtOH).

Synthesis of C3-Aryl Analogues (±)-15b and (±)-15c

Synthesis of (±)-16b

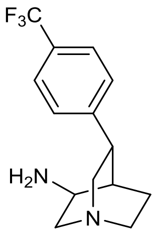

(±)-**4b** (650 mg, 1.73 mmol) was suspended in 50 ml H_2O to which 5 ml conc. HCl was added slowly with stirring. The reaction was stirred at room temperature for 5 min, before Zn dust (1.70 g, 26.00 mmol) was added. After 16 h, 65 ml DCM was added, and the reaction mixture was cooled using an ice-bath followed by slow addition of 5 M NaOH (100 ml). The mixture was filtered through celite and the pad was washed with 30 ml H_2O and 30 ml DCM. The combined filtrate was extracted three times with DCM (3 × 100 ml). The combined organic phase was dried over Na_2SO_4, filtered, and evaporated *in vacuo*. The crude material was purified by chromatography over silica gel. The combined organic phase was dried over Na_2SO_4 and filtered. The crude was purified by chromatography using a gradient of 20 to 40% DMA (CH_2Cl_2/MeOH/NH_4OH 80:20:3) in dichloromethane to afford the free amine as a colorless oil (361 mg, 77%). **^{1}H NMR (600 MHz, $CDCl_3$):** 7.58 (d, J = 8.3 Hz, 2H), 7.50 (d, J = 8.3 Hz, 2H), 3.47–3.38 (m, 2H), 3.23 (ddd, J = 14.0, 9.4 Hz, 1H), 3.07 (t, J = 9.1 Hz, 1H), 3.03–3.00 (m, 1H), 2.90–2.79 (m, 2H), 2.56–2.53 (m, 1H), 2.39–2.37 (m, 1H), 1.86–1.81 (m, 1H), 1.67–1.62 (m, 1H); **^{13}C NMR (150 MHz, $CDCl_3$):** 149.3 (C), 128.2 (q, J_{C-F} = 32.2 Hz, C), 127.4 (CH), 125.5 (q, J_{C-F} = 3.8 Hz, CH), 124.3 (q, J_{C-F} = 269.4 Hz, C), 59.6 (CH_2), 52.3 (CH_2), 50.0 (CH), 46.0 (CH_2), 38.8 (CH), 35.8 (CH), 29.5 (CH_2); **IR (ATR, neat, cm^{-1}):** 2929, 2872, 1616, 1326, 1163, 1117, 1069, 1016, 842, 807; **HRMS (ESI)** calcd. for $C_{14}H_{18}F_3N_2$ $[M+H]^+$: 271.1422, found: 271.1424.

Synthesis of (±)-16c

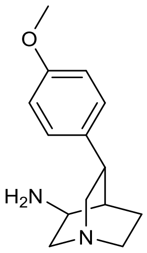

(±)-**4c** (710 mg, 2.11 mmol) was suspended in 50 ml H_2O to which 5 ml conc. HCl were added slowly with stirring. The reaction was stirred at room temperature for 5 min, before Zn dust (2.07 g, 31.60 mmol) was added. After 16 h, 65 ml DCM was added, and the reaction mixture was cooled using an ice-bath followed by slow addition of 5 M NaOH (100 ml). The mixture was filtered through celite and the pad was washed with 30 ml H_2O and 30 ml DCM. The combined filtrate was extracted with DCM (3 × 100 ml). The combined organic phase was dried over Na_2SO_4, filtered, and evaporated *in vacuo*. The crude material was purified by chromatography over silica gel. The combined organic phase was dried over Na_2SO_4 and filtered. The crude was purified by chromatography using a gradient of 20 to 40% DMA (CH_2Cl_2/MeOH/NH_4OH 80:20:3) in dichloromethane to afford the free amine as a colorless oil (441 mg, 90%). **^{1}H NMR (500 MHz, $CDCl_3$):** δ 7.27 (d, J = 8.0 Hz, 2H), 6.86 (d, J = 8.0 Hz, 2H), 3.78 (s, 3H), 3.39 (ddd, J = 13.5, 7.7, 1.8 Hz, 1H), 3.30 (t, J = 13.5, 8.5, 1.8 Hz, 1H), 3.22 (ddd, J = 14.0, 9.5, 2.2 Hz, 1H), 3.00 (t, J = 8.5 Hz, 1H), 2.93–2.89 (m, 1H), 2.87–2.72 (m, 2H), 2.52 (ddd, J = 14.0, 5.4, 2.2 Hz, 1H), 2.28–2.24 (m, 1H), 1.82–1.75 (m, 1H), 1.63–1.56 (m, 1H) 1.43 (br s, 2H); **^{13}C NMR (125 MHz, $CDCl_3$):** δ 157.7 (C), 136.2 (C), 127.8 (CH), 114.0 (CH), 59.3 (CH_2), 55.3 (CH_3), 52.2 (CH_2), 50.0 (CH), 45.9 (CH_2), 37.7 (CH), 36.1 (CH), 29.4 (CH_2); **IR (ATR, neat, cm^{-1}):** 3367, 2934, 2868, 1667, 1610, 1581, 1513, 1456, 1320, 1282, 1247, 1181, 1118, 1034; **HRMS (ESI)** calcd. for $C_{14}H_{20}N_2ONa$ $[M+Na]^+$: 255.1468, found: 255.1466.

Synthesis of (±)-11b

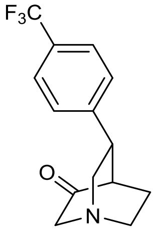

(±)-**16b** (200 mg, 0.74 mmol) was dissolved in 1.5 ml of DMA. L-Ascorbic acid (391 mg, 2.22 mmol) and copper 3-methylsalicylate (159 mg, 0.74 mmol) were successively added and the reaction mixture was stirred in open air at room temperature. After 17 h the brown-purple viscous oil was poured into an ice/water mixture. 5 M NaOH was added and the biphasic system was extracted with DCM. The combined organic layers were dried (Na_2SO_4), filtered and concentrated in vacuo. The resulting crude material was purified by flash chromatography (silica gel, CH_2Cl_2/4% MeOH) to afford the quinuclidone as a yellow oil (140 mg, 70%). **^{1}H NMR (600 MHz, $CDCl_3$):** 7.55 (d, J = 8.3 Hz, 2H), 7.23 (d, J = 8.3 Hz, 2H), 3.65–3.61 (m, 1H), 3.52–3.49 (m, 1H), 3.37 (AB system, J_{AB} = 19.0 Hz, 2H), 3.10–3.00 (m, 2H), 2.85 (ddd, J = 14.0, 7.3, 2.2 Hz, 1H), 2.71–2.69 (m, 1H), 2.23–2.18 (m, 1H), 2.13–2.07 (m, 1H); **^{13}C NMR (150 MHz, $CDCl_3$):** δ 218.1 (C), 147.7 (q, J_{C-F} = 1.4 Hz, C), 129.2 (q, J_{C-F} = 32.7 Hz, C), 127.3 (CH), 125.7 (q, J_{C-F} = 3.7 Hz, CH), 124.2 (q, J_{C-F} = 271.9 Hz, C), 63.5 (CH_2), 56.4 (CH_2), 45.8 (CH_2), 45.4 (CH), 44.8 (CH), 26.7 (CH_2); **IR (ATR, neat, cm^{-1}):** 2923, 1726, 1619, 1326, 1164, 1117, 1070, 1016, 838, 813; **HRMS (ESI)** calcd. for $C_{14}H_{16}F_3NO$ $[M+H]^+$: 270.1106, found: 270.1096.

Synthesis of (±)-11c

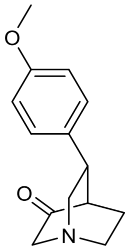

Due to scaleability reasons, the following reaction has been carried out by running multiple reactions parallel and combining the crude mixtures for purification. The yield is calculated by dividing by the number of individual reactions and therefore an average value of 20 reactions is given. (±)-**16c** (31 mg, 0.13 mmol) was dissolved in 266 µl of DMA. L-Ascorbic acid (71 mg, 0.40 mmol) and copper 3-methylsalicylate (29 mg, 0.13 mmol) were successively added and the reaction mixture was stirred in open air at room temperature. After 17 h the brown-purple viscous oil was poured into an ice/water mixture. 5 M NaOH was added and the biphasic system was extracted with CH_2Cl_2. The combined organic layers were dried (Na_2SO_4), filtered and concentrated *in vacuo*. The resulting crude material was purified by flash chromatography (silica gel, CH_2Cl_2/4% MeOH) to afford the quinuclidone as an off-white solid (17 mg, 56%). **^{1}H NMR (500 MHz, CD_2Cl_2):** δ 6.95 (d, J = 8.6 Hz, 2H), 6.74 (d, J = 8.6 Hz, 2H), 3.68 (s, 3H), 3.43 (ddd, J = 13.0, 10.0, 1.9 Hz, 1H), 3.30 (ddd, J = 11.8, 7.1, 1.8 Hz, 1H), 3.22 (m, 2H), 2.96–2.83 (m, 2H), 2.45–2.42 (m, 1H), 2.11–2.03 (m, 1H), 1.97–1.89 (m, 1H); **^{13}C NMR (125 MHz, CD_2Cl_2):** δ 218.8 (C), 158.7 (C), 136.5 (C), 128.3 (CH), 114.4 (CH), 63.7 (CH_2), 63.7 (CH_2), 56.7 (CH_2), 55.6 (CH_3), 47.0 (CH), 46.1 (CH_2), 44.7 (CH), 27.1 (CH_2); **IR (ATR, neat, cm^{-1}):** 2927, 2873, 1723, 1611, 1582, 1513, 1456, 1305, 1282, 1247, 1181, 1116, 1097, 1074, 1032; **HRMS (ESI)** calcd. for $C_{14}H_{17}NO_2Na$ $[M+Na]^+$: 254.1151, found: 254.1147.

Synthesis of (±)-17b

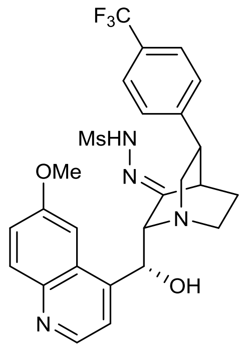

(±)-**11b** (140 mg, 0.52 mmol) was dissolved in 5 ml of anhydrous THF under argon and cooled to 0 °C, followed by addition of LiHMDS (1.0 M in THF, 0.55 mL, 0.55 mmol). The reaction was stirred for 30 min at 0 °C, then cooled to −78 °C and 6-methoxyquinoline-4-carbaldehyde **12** (107 mg, 0.57 mmol) was added neat. After 1 h at −78 °C, Ti(^{i}OPr)$_3$Cl (339 mg, 1.30 mmol) was added and the reaction was brought to 0 °C and stirred for 5 min, followed by addition of methanesulfonyl hydrazide (115 mg, 1.04 mmol). After 5 min at 0 °C, the reaction was brought to room temperature and stirred for 3 h, then quenched at 0 °C using 1.0 ml sat. aq. $NaHCO_3$. The mixture was extracted from sat. aq. $NaHCO_3$ (30 ml) with DCM (3 × 30 mL). The combined organic phase was dried over Na_2SO_4 and filtered. The crude was purified by chromatography using a gradient of 10 to 30% DMA (CH_2Cl_2/MeOH/NH_4OH 80:20:3) in dichloromethane to afford the product as a white powder (269 mg, 94%, dr > 16:1). **^{1}H NMR (700 MHz, $CDCl_3$):** 8.20 (d, J = 4.4 Hz, 1H), 7.75 (d, J = 9.2 Hz, 1H), 7.58 (br s, 1H), 7.52 (d, J = 8.2 Hz, 2H), 7.27–7.24 (m, 4H), 5.51 (d, J = 9.2 Hz, 1H), 4.31 (d, J = 9.3 Hz, 1H), 3.90 (s, 3H), 3.28–3.24 (m, 2H) 3.00–2.97 (m, 3H), 2.94 (s, 3H), 2.82 (br s, 1H), 2.61–2.51 (m, 2H), 2.15–2.10 (m, 1H), 2.03–1.99 (m, 1H); **^{13}C NMR (175 MHz, $CDCl_3$):** 160.3 (C), 158.3 (C), 146.9 (C), 146.6 (CH), 145.1 (C), 144.1 (C), 130.9 (CH), 129.1 (q, J_{C-F} = 32.5 Hz, C), 128.5 (CH), 126.9 (C), 125.4 (q, J_{C-F} = 3.7 Hz, CH), 124.3 (q, J_{C-F} = 271.7 Hz, C), 123.4 (CH), 120.2 (CH), 101.9 (CH), 72.8 (CH), 66.6 (CH), 57.2 (CH_2), 56.0 (CH_3), 46.7 (CH), 41.0 (CH_2), 39.3 (CH), 38.4 (CH_3), 24.4 (CH_2); **IR (ATR, neat, cm^{-1}):** 2959, 1621, 1511, 1474, 1325, 1244, 1159, 1119, 1069, 1029, 1017, 973, 911, 835, 731, 618; **HRMS (ESI)** calcd. for $C_{26}H_{29}F_3N_4O_4S$ $[M+H]^+$: 549.1783, found: 549.1782.

Synthesis of (±)-17c

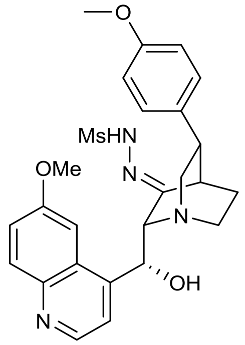

(±)-**11c** (92.5 mg, 0.40 mmol) was dissolved in 4 ml of anhydrous THF under argon and cooled to 0 °C, followed by addition of LiHMDS (1.0 M in THF, 0.42 mL, 0.42 mmol). The reaction was stirred for 30 min at 0 °C, then cooled to −78 °C and 6-methoxyquinoline-4-carbaldehyde **12** (78.6 mg, 0.42 mmol) was added neat. After 1 h at −78 °C, Ti(^{i}OPr)$_3$Cl (261 mg, 1.00 mmol) was added and the reaction was brought to 0 °C and stirred for 5 min, followed by addition of methanesulfonyl hydrazide (132 mg, 1.20 mmol). After 5 min at 0 °C, the reaction was brought to room temperature and stirred for 3 h, then quenched at 0 °C using 1.0 ml sat. aq. $NaHCO_3$. The mixture was extracted from sat. aq. $NaHCO_3$ (30 ml) with DCM (3 × 30 ml). The combined organic phase was dried over Na_2SO_4 and filtered. The crude was purified by chromatography using a gradient of 10 to 30% DMA (CH_2Cl_2/MeOH/NH_4OH 80:20:3) in dichloromethane to afford the product as a pale yellow solid (192 mg, 94%, dr > 16:1). **^{1}H NMR (600 MHz, $CDCl_3$):** δ = 8.30 (d, J = 4.5 Hz, 1H), 7.79 (d, J = 9.2 Hz, 1 H), 7.59 (d, J = 1.7 Hz, 1H), 7.30–7.27 (m, 2H), 7.01 (d, J = 8.6 Hz, 2H), 6.79 (d, J = 8.6 Hz, 2H), 5.51 (d, J = 9.2 Hz, 1H), 4.29 (d, J = 9.2 Hz, 1H), 3.91 (s, 3H), 3.75 (s, 3H), 3.23 (dd, J = 13.6, 9.9 Hz, 1H), 3.19–3.14 (m, 1H), 3.02–2.93 (m, 4H), 2.74 (bs, 1H), 2.62–2.55 (m, 1H), 2.55–2.49 (m, 1H), 2.12–2.04 (m, 1H), 2.01–1.94 (m, 1H); **^{13}C NMR (150 MHz, $CDCl_3$):** 160.9 (C), 158.5 (C), 158.2 (C), 146.9 (CH), 145.1 (C), 144.3 (C), 134.9 (C), 131.2 (CH), 129.1 (CH), 126.9 (C), 122.3 (CH), 120.1 (CH), 113.8 (CH), 101.9 (CH), 72.8 (CH), 66.6 (CH), 57.7 (CH_2), 56.0 (CH_3), 55.4 (CH_3), 46.3 (CH), 41.0 (CH_2), 39.9 (CH), 38.5 (CH_3), 24.5 (CH_2); **IR (ATR, neat, cm^{-1}):** 3056, 2936, 2836, 2172, 2041, 1621, 1592, 1512, 1473, 1433, 1325, 1286, 1246, 1181, 1155, 1109, 1084, 1031, 973, 919, 859, 829, 751, 735,

716, 620, 583, 568, 530; **HRMS (ESI)** calcd. for $C_{26}H_{31}N_4O_5S$ $[M+H]^+$: 511.2010, found: 511.2011.

Synthesis of (±)-15b

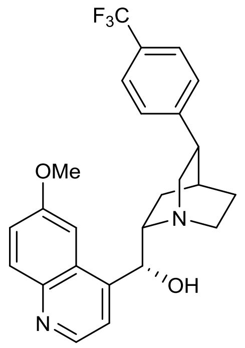

Lithium aluminium hydride solution (1.0 M in THF, 4.4 eq, 0.22 mL, 0.22 mmol) was diluted with THF (0.5 mL) in a Schlenk flask under argon. Methanol (8.8 eq, 18 µL, 0.44 mmol) was added dropwise at 0°C and stirred at this temperature for 10 min. In another Schlenk flask, a solution of hydrazone (±)-**17b** (1 eq, 30 mg, 0.05 mmol) was diluted in THF (0.5 mL). The aluminium hydride solution was added dropwise to the solution of hydrazone at 0 °C. The reaction mixture was then warmed to room temperature and stirred for an additional 10 min before being quenched by saturated aqueous $NaHCO_3$ solution at 0 °C. The mixture was extracted from saturated aqueous $NaHCO_3$ with three volumes of DCM. The combined organic phase was dried over Na_2SO_4 and filtered. The crude was purified by chromatography using a gradient of 10 to 30% DMA (CH_2Cl_2/MeOH/NH_4OH 80:20:3) in dichloromethane to afford the product as a white powder (5 mg, 21%). **^{1}H NMR (600 MHz, $CDCl_3$):** 8.72 (d, J = 4.5 Hz, 1H), 8.02 (d, J = 9.2 Hz, 1H), 7.53 (d, J = 4.6 Hz, 1H), 7.50 (d, J = 8.2 Hz, 2H), 7.37 (dd, J = 9.2, 2.7 Hz, 1H), 7.32 (d, J = 2.4 Hz, 1H), 7.25 (d, J = 8.4 Hz, 2H), 5.70 (bs, 1H), 2.94 (s, 3H), 3.65–3.57 (m, 1H), 3.43–3.38 (m, 1H), 3.32–3.26 (m, 1H), 3.17–3.12 (m, 1H), 3.07–3.02 (m, 1H), 2.80–2.73 (m, 1H), 2.14–2.10 (m, 1H), 1.95–1.90 (m, 1H), 1.85–1.79 (m, 1H), 1.74–1.67 (m, 1H), 1.48–1.41 (m, 1H); **^{13}C NMR (150 MHz, MeOD):** 159.8 (C), 150.5 (C), 149.7 (C), 148.2 (CH), 144.9 (C), 131.4 (CH), 129.5 (C), 129.2 (CH), 128.2 (C), 126.3 (CH), 125.7 (q, J_{C-F} = 269.3 Hz, C), 123.4 (CH), 120.3 (CH), 102.6 (CH), 72.0 (CH), 61.4 (CH), 57.3 (CH_2), 56.5 (CH_3), 44.2 (CH_2), 42.1 (CH), 30.3 (CH), 28.8 (CH_2), 21.6 (CH_2); **IR (ATR, neat, cm^{-1}):** 2925, 2855, 1620, 1510, 1457, 1433, 1327, 1241, 1165, 1117, 1069, 1029, 1017, 837; **HRMS (ESI)** calcd. for $C_{25}H_{26}F_3N_2O_2$ $[M+H]^+$: 443.1942, found: 443.1932.

Synthesis of (±)-15c

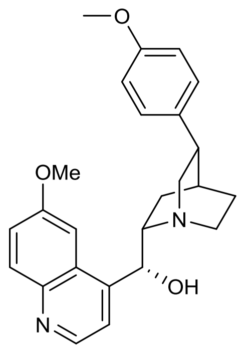

Lithium aluminium hydride (1 M in THF, 4.4 eq, 440 µL, 0.44 mmol) was diluted with dry THF (1 mL) in a flame-dried Schlenk tube and cooled to 0 °C. Dry methanol (13.2 eq, 53.5 µL, 1.32 mmol) was added and the solution was stirred for 10 min. The mixture was transferred with a syringe to a solution of mesylhydrazone (±)-**17c** (1 eq, 51.1 mg, 0.1 mmol) in dry THF (1 mL) at 0 °C. The ice bath was removed, and the solution was stirred for 15 h while warming to room temperature. After recooling to 0 °C, the mixture was quenched dropwise with saturated aqueous $NaHCO_3$ (5 mL) and extracted with DCM (1 × 15 mL, 2 × 5 mL). The solution was dried over $MgSO_4$, filtered and concentrated under reduced pressure. Purification by preparative thin layer chromatography (silica, DCM/MeOH/NH_4OH 94:6:0.05) afforded the product as off-white solid (7.0 mg, 17%). **^{1}H NMR (600 MHz, MeOD):** 8.65 (d, J = 4.6 Hz, 1H), 7.96 (d, J = 9.2 Hz, 1 H), 7.68 (d, J = 4.6 Hz, 1H), 7.48 (d, J = 2.6 Hz, 1H), 7.47 (dd, J = 9.2, 2.6 Hz, 1H), 7.07 (d, J = 8.7 Hz, 2H), 6.76 (d, J = 8.7 Hz, 2H), 5.64 (d, J = 3.3 Hz, 1H), 4.01 (s, 3H), 3.78–3.72 (m, 1H), 3.69 (s, 3H), 3.40–3.35 (m, 1H), 3.34–3.29 (m, 1H), 3.21–3.14 (m, 1H), 3.06–3.00 (m, 1H), 2.89–2.82 (m, 1H), 2.04–1.96 (m, 2H), 1.92–1.86 (m, 1H), 1.82–1.74 (m, 1H), 1.40–1.34 (m, 1H) **^{13}C NMR (150 MHz, MeOD):** 159.8 (C), 159.5 (C), 150.2 (C), 148.2 (CH), 144.8 (C), 136.6 (C), 131.5 (CH), 129.3 (CH), 128.1 (C), 123.4 (CH), 120.3 (CH), 114.9 (CH), 102.5 (CH), 72.0 (CH), 61.4 (CH), 57.7 (CH_2), 56.5 (CH_3), 55.6

(CH_3), 44.4 (CH_2), 41.3 (CH), 30.5 (CH), 28.6 (CH_2), 21.4 (CH_2); **IR (ATR, neat, cm^{-1}):** 3186, 2936, 2835, 1620, 1590, 1511, 1468, 1434, 1363, 1324, 1244, 1180, 1135, 1100, 1081, 1032, 855, 831, 752, 717, 642; **HRMS (ESI)** calcd. for $C_{25}H_{29}N_2O_3$ $[M+H]^+$: 405.2173, found: 405.2187.

Purification of (±)-15b and (±)-15c

Purification of compounds (±)-**15b** and (±)-**15c** for *in vivo* experiments was performed by preparative HPLC (column Waters, Xselect CSH prep C18, 5 µm, 30 × 150 mm, flow = 20 ml/min). Mobile Phase: CH_3CN (**A**) and 10 mM NH_4HCO_3 solution in water (**B**).
(±)-**15b**: 40% **B** for 2 min, gradient from 40% to 80% in 15 min, then 95% **B**: t_R = 12.91 min;
(±)-**15c**: 30% **B** for 2 min, gradient from 30% to 70% in 22 min, then 95% **B**: t_R = 15.10 min;

Purity of compounds (±)-**15b** and (±)-**15c** for *in vivo* experiments was determined by analytical HPLC (column Waters, Xselect CSH C18, 5 µm, 4.6 × 100 mm, flow = 1 ml/min) at 220 and 254 nm:

(±) 15b: 40% **B** for 2 min, gradient from 40% to 60% in 10 min, then 95% **B**. t_R = 8.81 min; purity > 99%.

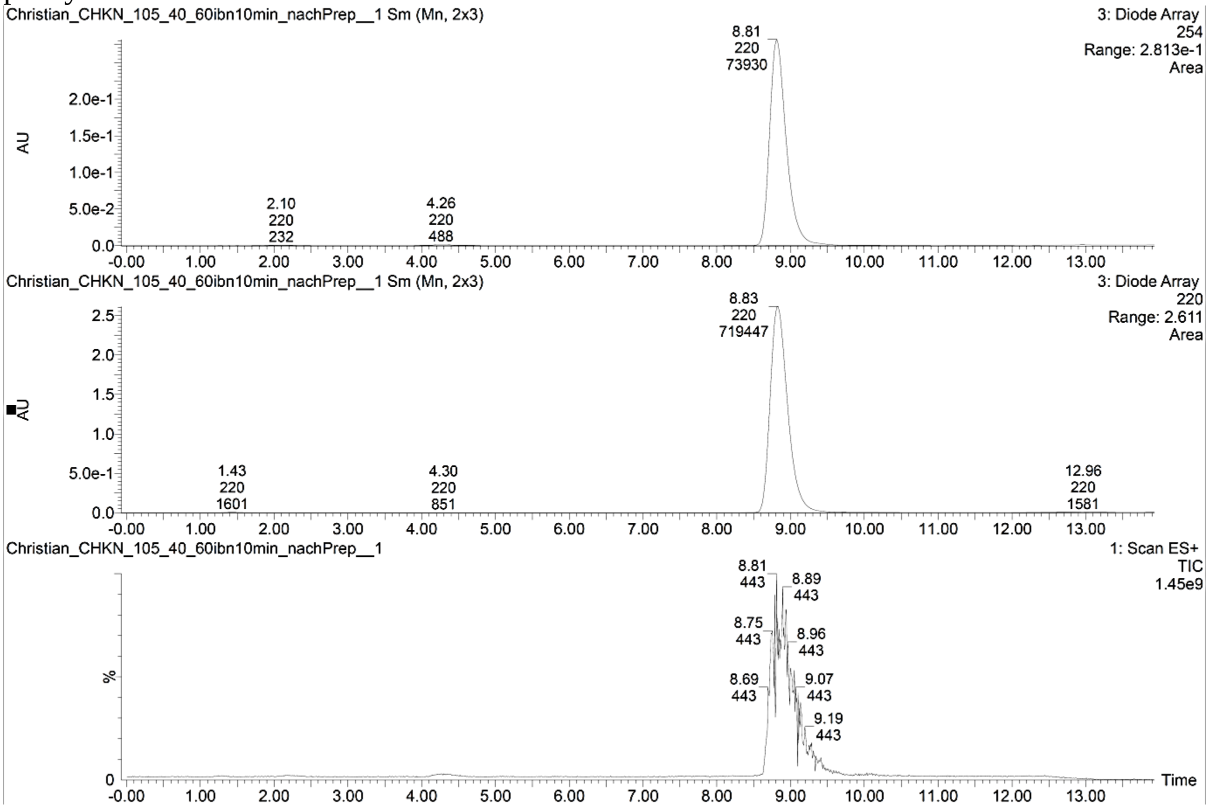

(±) 15c: 30% **B** for 2 min, gradient from 30% to 60% in 10 min, then 95% **B**. t_R = 8.08 min; purity > 99%

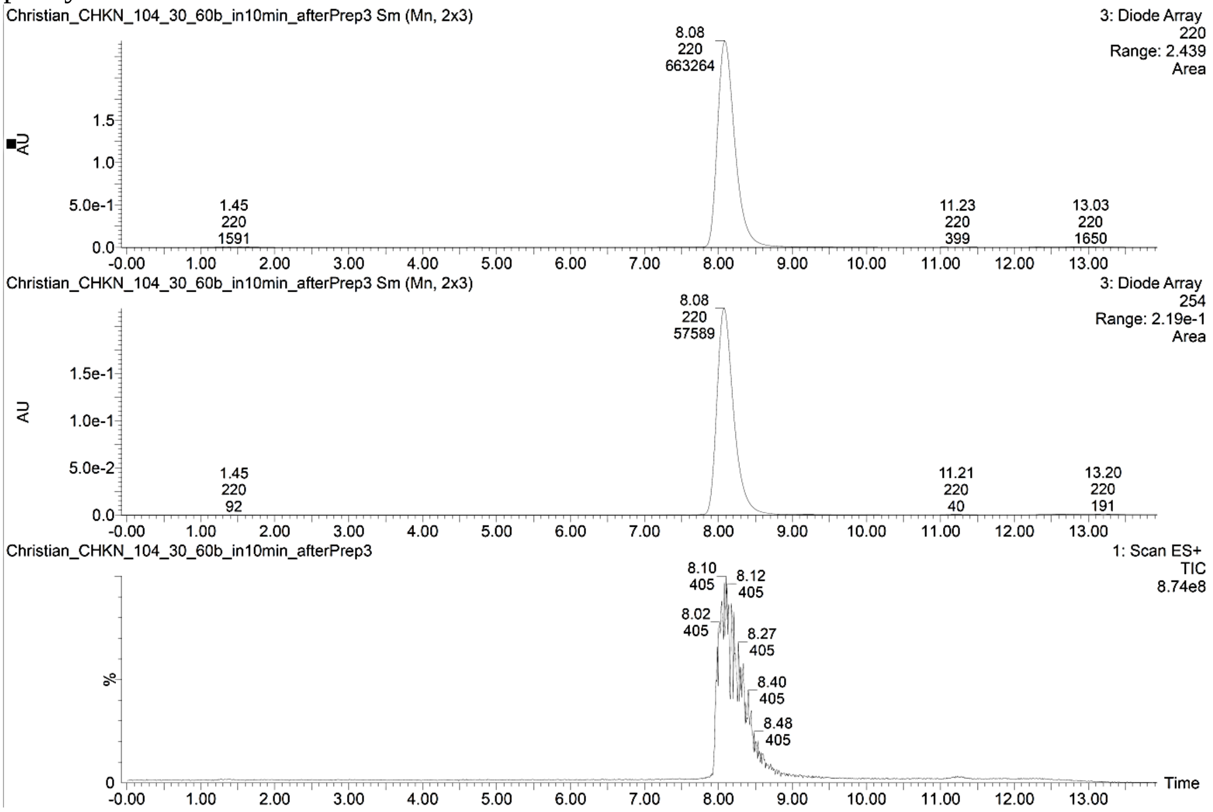

Compounds for Model Studies

Synthesis of (±)-13

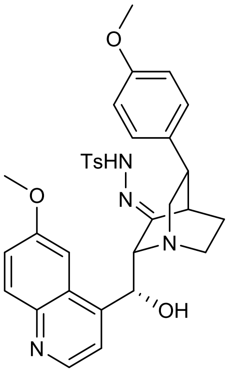

(±)-**11c** (145 mg, 0.63 mmol) was dissolved in 2.5 ml THF under argon and cooled to 0 °C, followed by addition of LiHMDS (1.0 M in THF, 66 µl, 0.66 mmol). The reaction was stirred for 30 min at 0 °C, then cooled to −78 °C and 6-methoxyquinoline-4-carbaldehyde **12** (129 mg, 0.69 mmol) was added neat. After 60 min at −78 °C, Ti(O-*i*-Pr)$_3$Cl (372 µl, 1.570 mmol) was added as a solution in 744 µl THF and the reaction was brought to 0 °C and stirred for 5 min, followed by addition of *p*-toluenesulfonyl hydrazide (neat, 234 mg, 1.26 mmol). After 5 min at 0 °C, the reaction was brought to room temperature and stirred for 2 h, then quenched at 0 °C using 5 ml sat. aq. $NaHCO_3$. The mixture was extracted from sat. aq. $NaHCO_3$ (200 ml) with three volumes of DCM (200 ml each). The combined organic phase was dried over Na_2SO_4 and filtered. A spatula-full of silica was added to the filtrate and solvents were removed *in vacuo*. The crude (adsorbed on silica) was purified by chromatography over silica gel using a gradient of 10 to 35% DMA (CH_2Cl_2/MeOH/NH_4OH 90:10:1.5) in dichloromethane to afford the product as an off-white solid (315 mg, 86%, dr > 16:1).

^{1}H NMR (300 MHz, MeOD): major isomer: δ = 8.70 (d, J = 4.6 Hz, 1H), 8.01 (d, J = 9.4 Hz, 1H), 7.93 (d, J = 2.6 Hz, 1H), 7.76 (d, J = 8.3 Hz, 2H), 7.72 (d, J = 4.6 Hz, 1H), 7.45 (dd, J = 9.4, 2.6 Hz, 1H), 7.35 (d, J = 8.3 Hz, 2H), 7.01 (d, J = 8.8 Hz, 2H), 6.72 (d, J = 8.8 Hz, 2H), 5.56 (d, J = 9.4 Hz, 1H), 4.55 (br s, 1H), 4.51 (d, J = 9.4 Hz), 4.05 (s, 3H), 3.77 (s, 3H), 3.27–3.14 (m, 3H), 2.74–2.56 (m, 2H), 2.51–2.39 (m, 4H), 2.15–1.93 (m, 2H); **^{13}C NMR (75 MHz, MeOD):** major isomer: δ = 162.6, 159.7, 159.5, 148.2, 147.9, 145.6, 145.2, 137.5, 136.3, 131.6,

130.6, 130.1, 129.0, 128.5, 123.5, 122.0, 114.7, 103.5, 74.3, 67.9, 59.0, 56.6, 55.7, 47.2, 41.5, 41.0, 25.3, 21.5. IR (ATR, neat, cm^{-1}): 3059, 2934, 2835, 1620, 1595, 1511, 1454, 1432, 1327, 1305, 1287, 1243, 1181, 1160, 1089, 1030. **HRMS (ESI)** calcd. for $C_{32}H_{35}N_4O_5S$ $[M+H]^+$: 587.2323, found: 587.2322.

Summary of Failed Attempts for C−H Functionalization

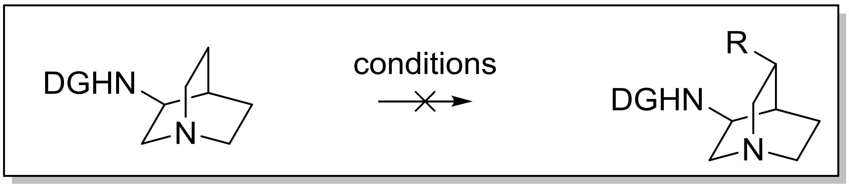

C−H Vinylation

Coupling partners investigated:

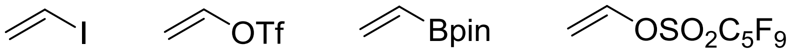

Directing groups investigated:

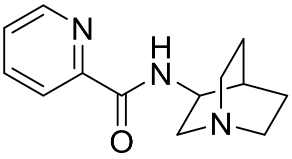

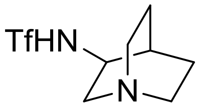

(also applied as Na^+ or ^-OTf salt)

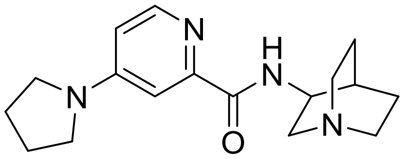

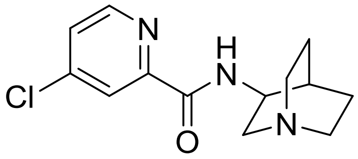

Pd-sources investigated: $Pd(OAc)_2$, $Pd(OTf)_2(MeCN)_4$, $Pd(dba)_2$

Additives investigated: none, $PivCO_2H$, N-Ac-Ile, PCy_3, DMSO, H_2O

Solvents: t-Amyl-OH, toluene, DMF, DMA, CH_3CN

Base additives: Ag_2CO_3, AgOAc, Li_2CO_3, $NaHCO_3$

Oxidant additives: none, benzoquinone

C−H Alkenylation

Coupling partners investigated:

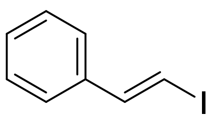

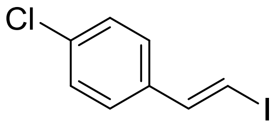

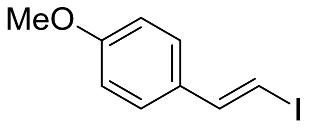

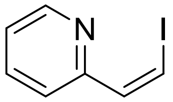

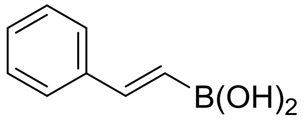

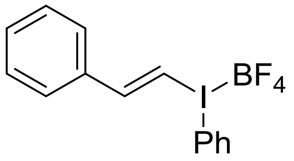

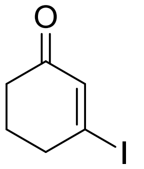

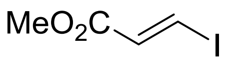

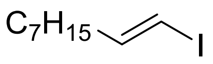

Directing groups investigated:

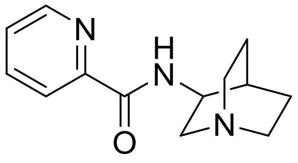

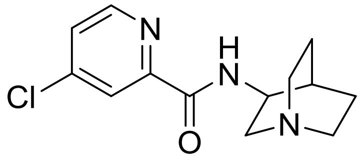

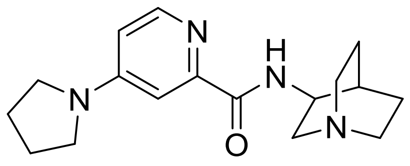

Pd-sources investigated: $Pd(OAc)_2$

Additives investigated: none, $PivCO_2H$, $(BnO)_2PO_2H$, pyridine, N-Boc-Ile

Solvents: none, t-Amyl-OH, toluene, THF, CH_3CN

Base additives: $Ag_2CO_{3,}$ AgOAc, $AgBF_{4,}$ $AgNTf_{2,}$ $KHCO_3$

Oxidant additives: none, $Cu(OAc)_{2,}$ benzoquinone

C−H Alkynylation

Coupling partners investigated:

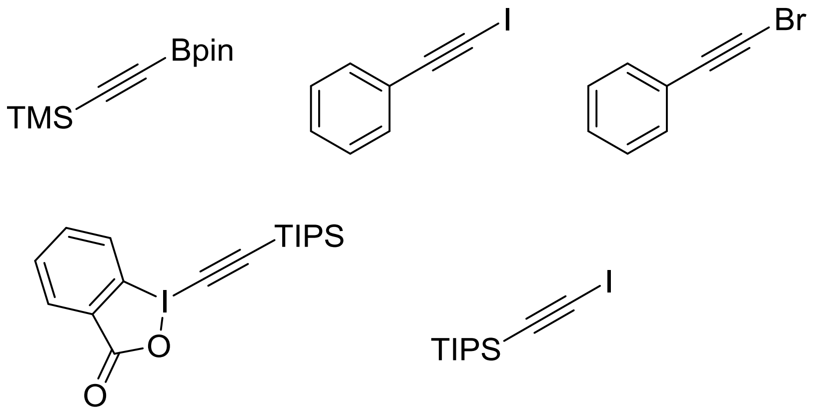

Directing groups investigated:

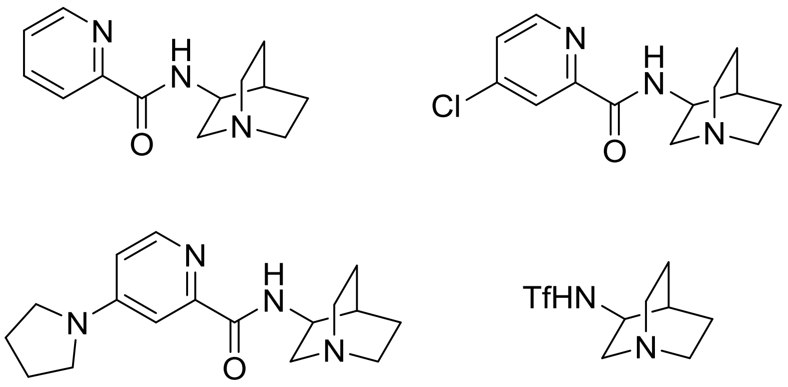

Pd-sources investigated: $Pd(OAc)_2$

Additives investigated: none, DMAP

Solvents: t-Amyl-OH, toluene

Base additives: Ag_2CO_3, AgOAc, Li_2CO_3

Oxidant additives: none, benzoquinone, N-Ac-Ile

C−H Alkylation

Coupling partners investigated:

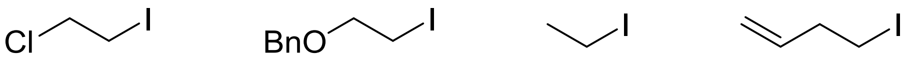

Directing groups investigated:

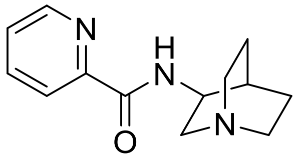

Pd-sources investigated: $Pd(OAc)_2$

Additives investigated: none, $PivCO_2H$, $(BnO)_2PO_2H$, CSA

Solvents: none, t-Amyl-OH, toluene

Base additives: Ag_2CO_3, AgOAc

Other Unsuccessful Coupling Reagents

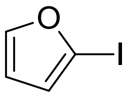

BnOH/PIDA

$B_2(pin)_2$

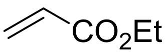

NBS

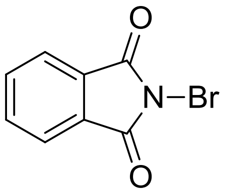

NMR Spectra in Numerical Order

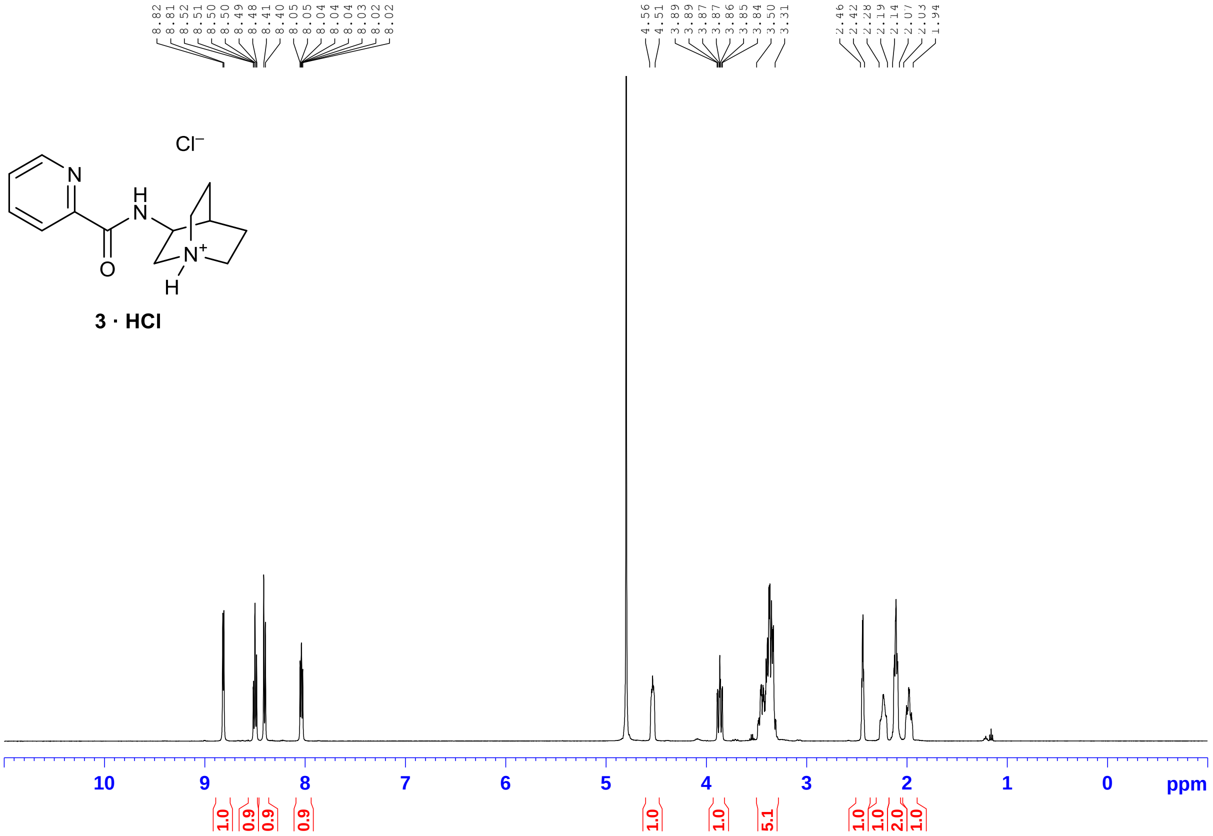

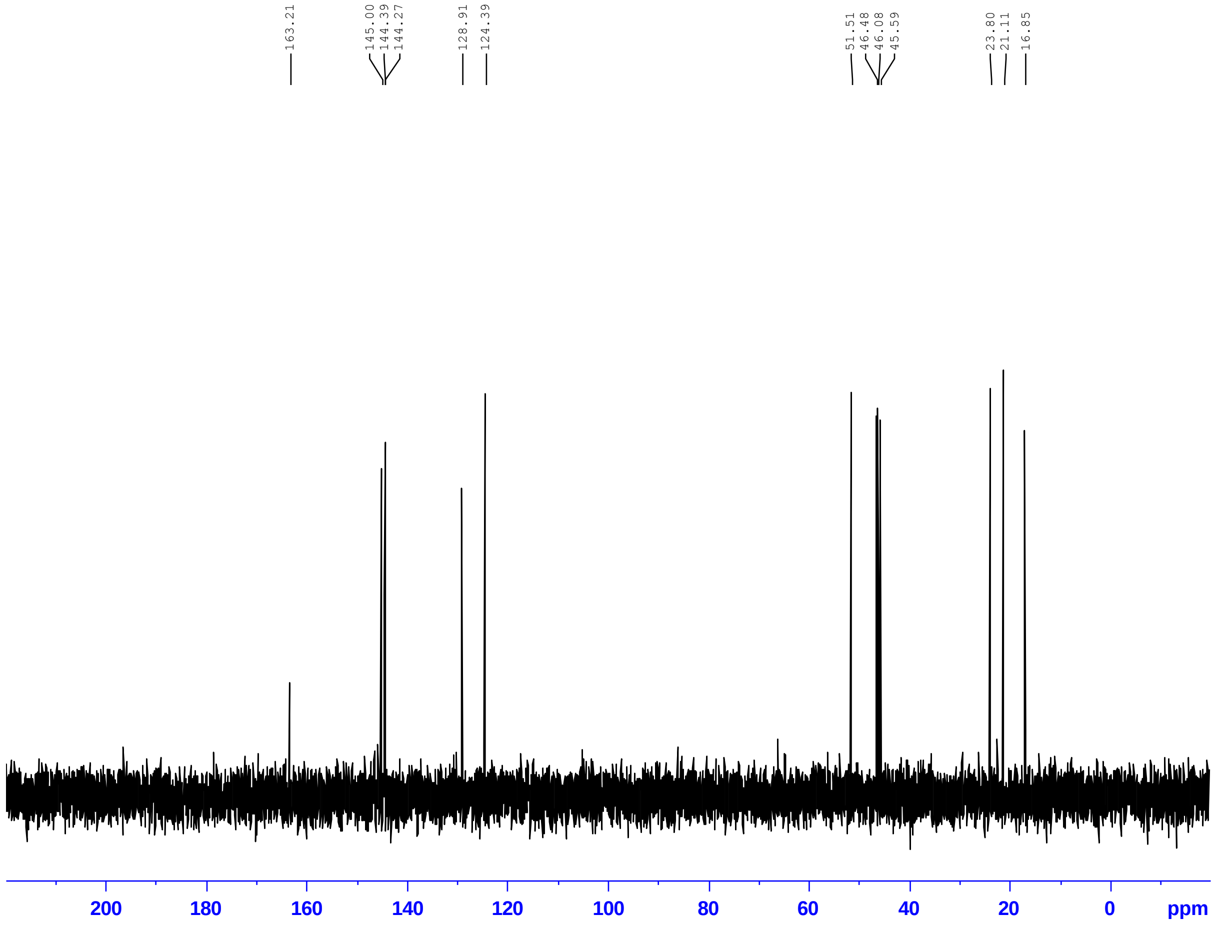

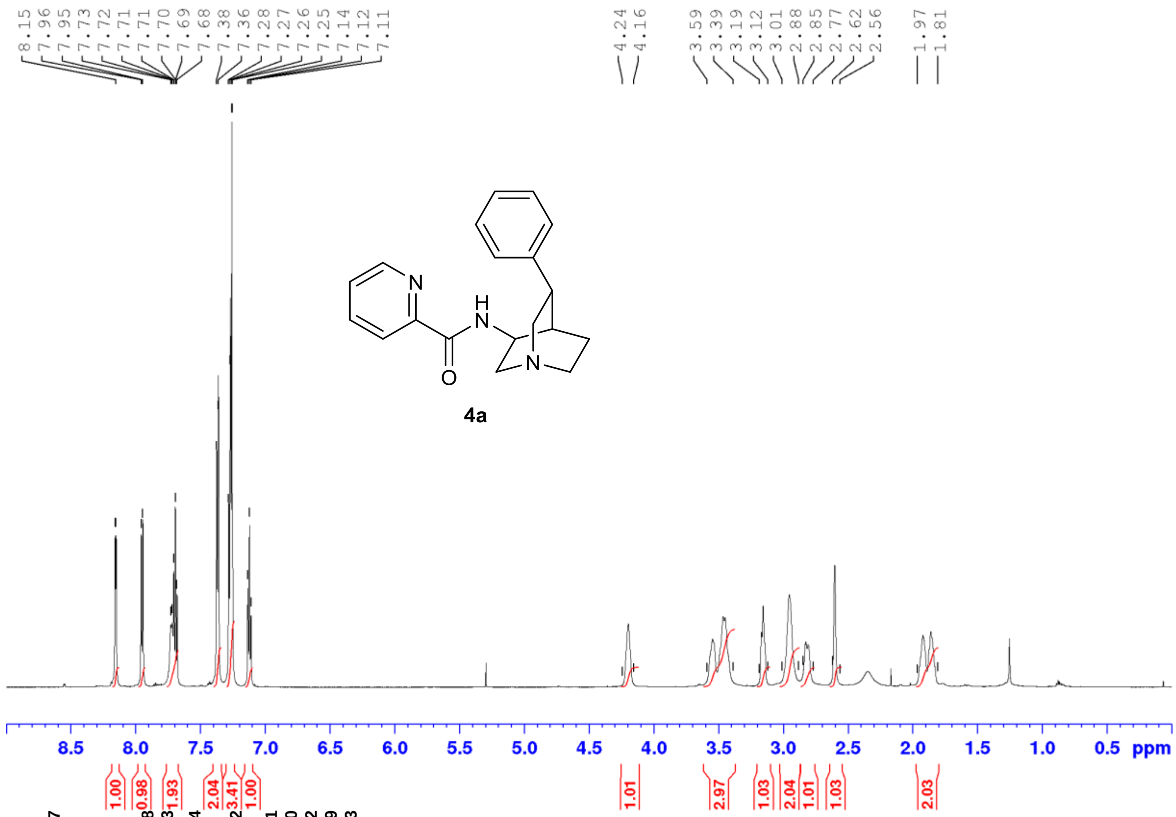
8.15 7.96 7.95 7.73 7.72 7.71 7.71 7.70 7.69 7.68 7.38 7.36 7.28 7.27 7.26 7.25 7.14 7.12 7.11
4.24 4.16
3.59 3.39 3.19 3.12 3.01 2.88 2.85 2.77 2.62 2.56
1.97 1.81
N
H
N
O
N
4a
8.5 8.0 7.5 7.0 6.5 6.0 5.5 5.0 4.5 4.0 3.5 3.0 2.5 2.0 1.5 1.0 0.5 ppm
1.00 0.98 1.93 2.04 3.41 1.00 1.01 2.97 1.03 2.04 1.01 1.03 2.03

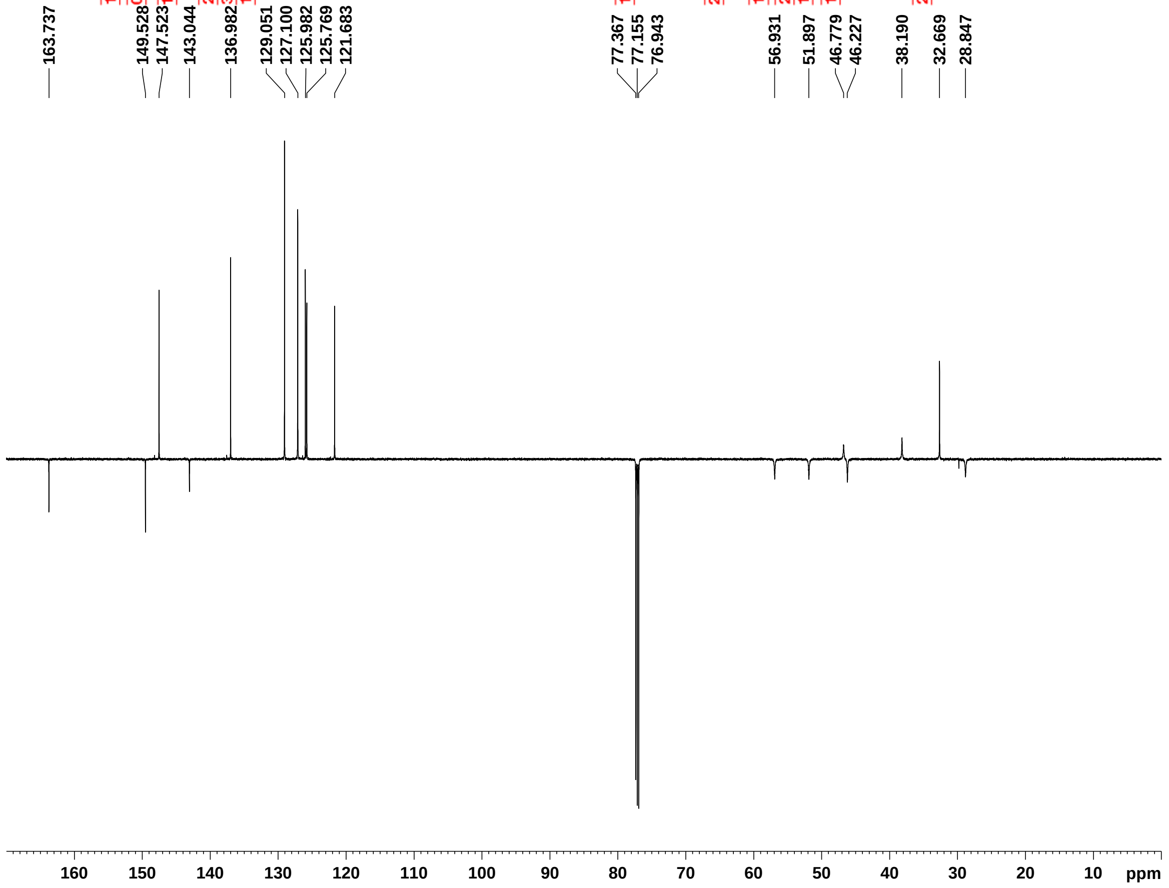
163.737
149.528 147.523 143.044 136.982 129.051 127.100 125.982 125.769 121.683
77.367 77.155 76.943
56.931 51.897 46.779 46.227 38.190 32.669 28.847
160 150 140 130 120 110 100 90 80 70 60 50 40 30 20 10 ppm

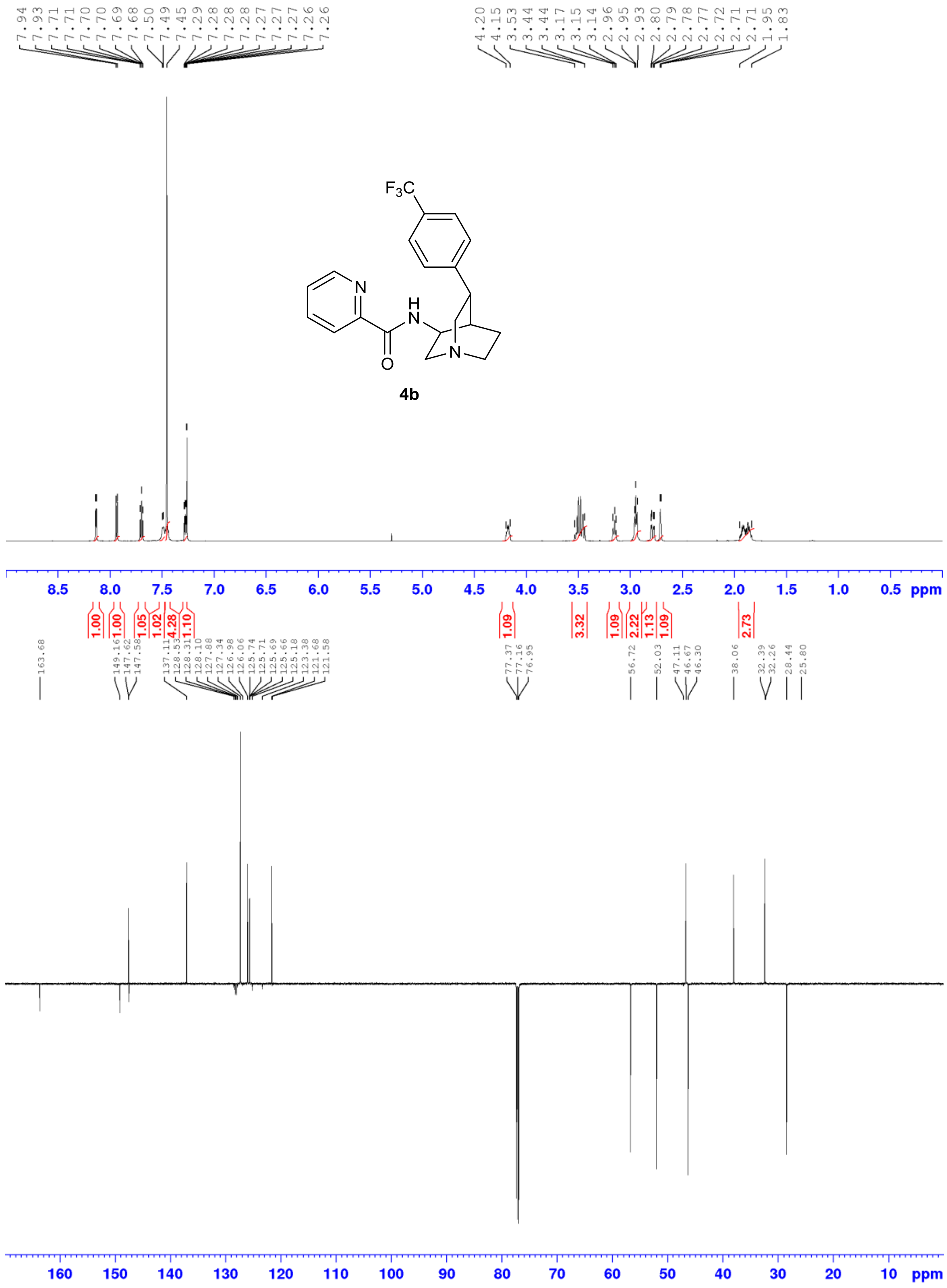

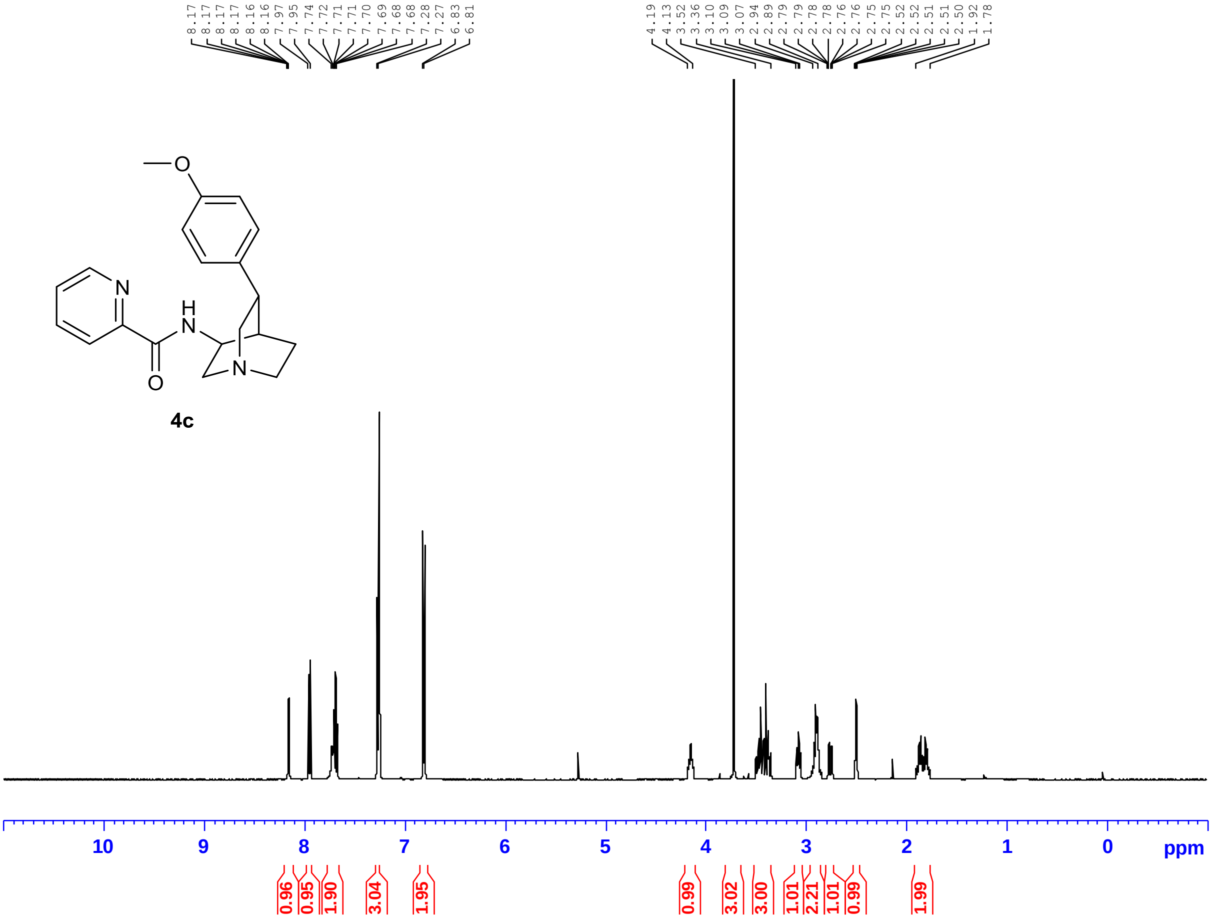
8.17
8.17
8.17
8.17
8.16
8.16
7.97
7.95
7.74
7.72
7.71
7.71
7.70
7.69
7.68
7.68
7.28
7.27
6.83
6.81
4.19
4.13
3.52
3.36
3.10
3.09
3.07
2.94
2.89
2.79
2.79
2.79
2.78
2.78
2.76
2.76
2.75
2.75
2.52
2.52
2.51
2.51
2.50
1.92
1.78
O
N
H
N
O
N
4c
10
9
8
7
6
5
4
3
2
1
0
ppm
0.96
0.95
1.90
3.04
1.95
0.99
3.02
3.00
1.01
2.21
1.01
0.99
1.99

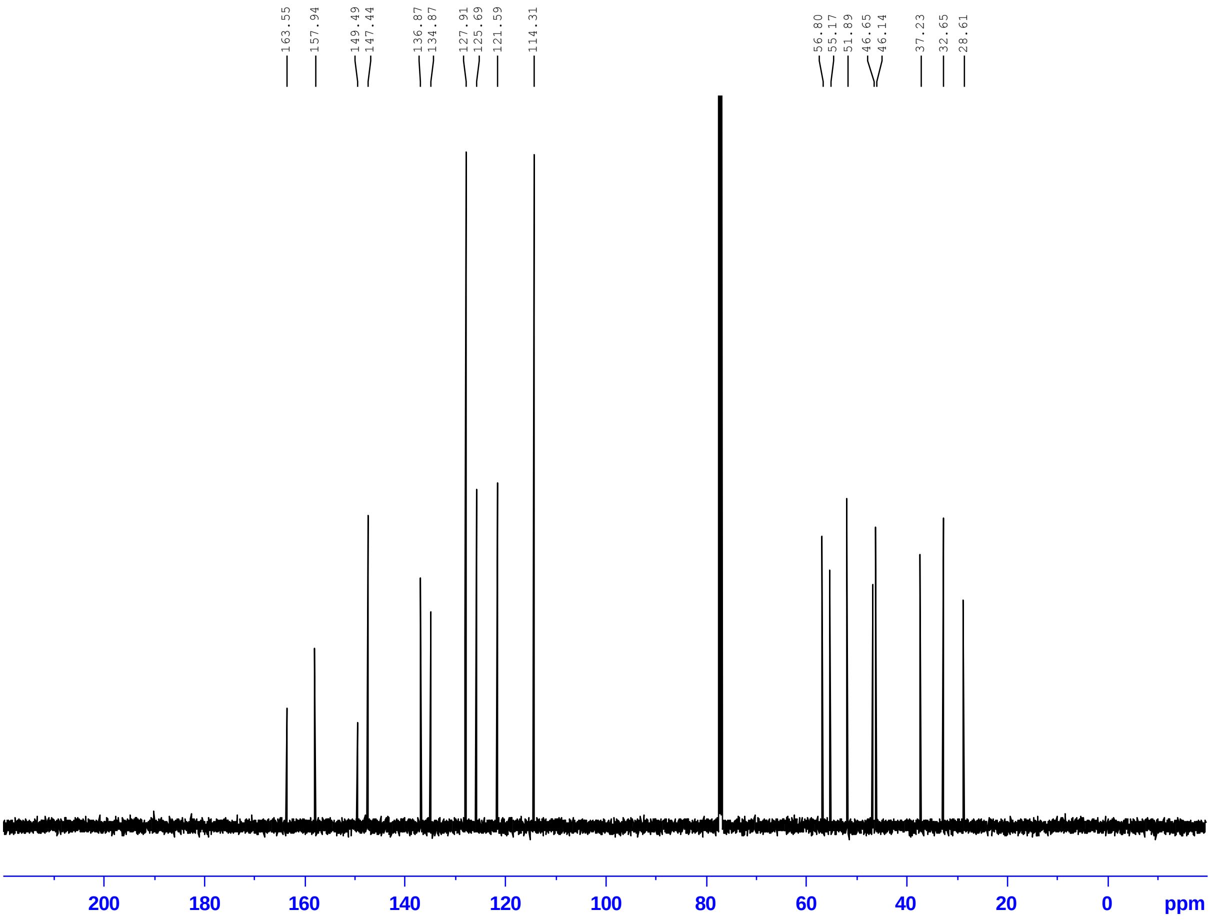
163.55
157.94
149.49
147.44
136.87
134.87
127.91
125.69
121.59
114.31
56.80
55.17
51.89
46.65
46.14
37.23
32.65
28.61
200
180
160
140
120
100
80
60
40
20
0
ppm

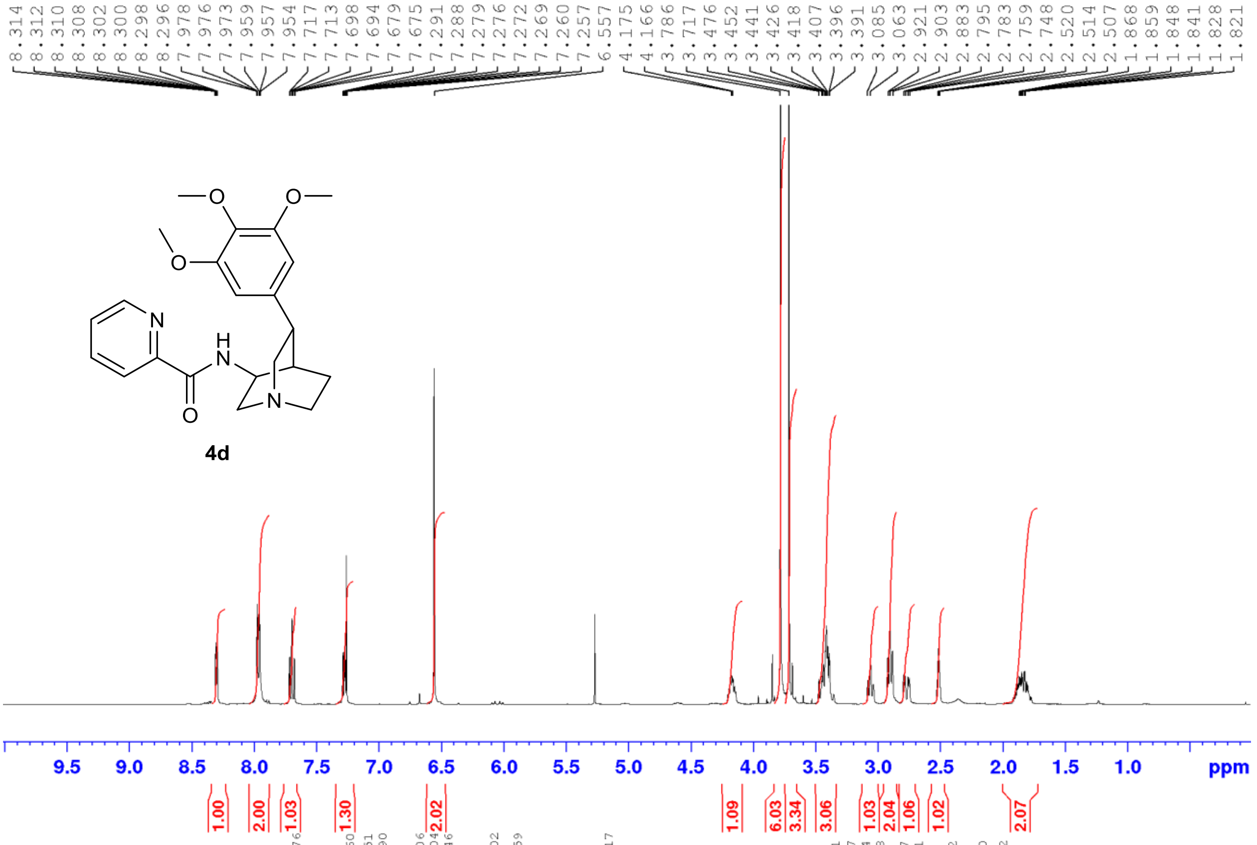
4d

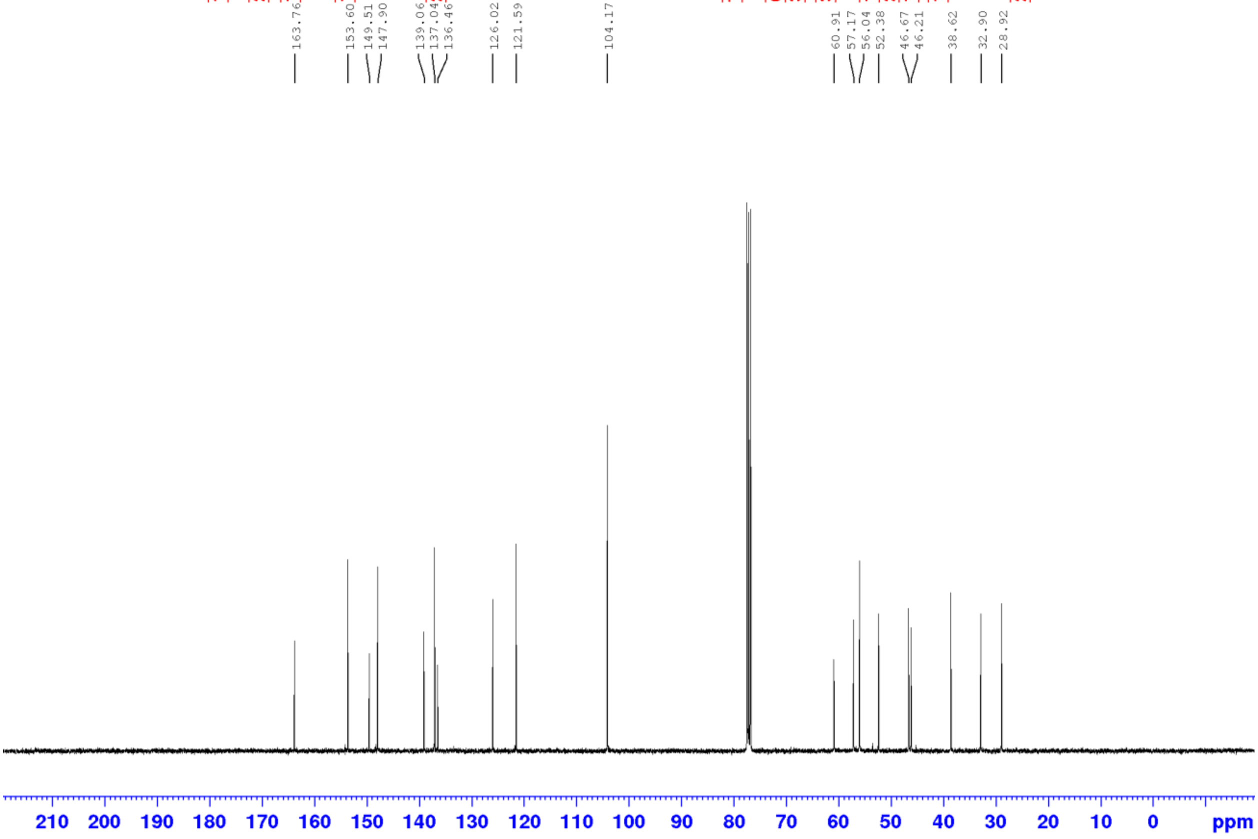

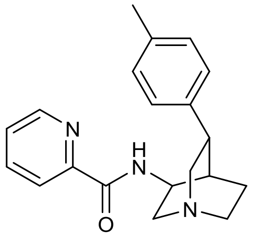
N
H
N
N
O

4e

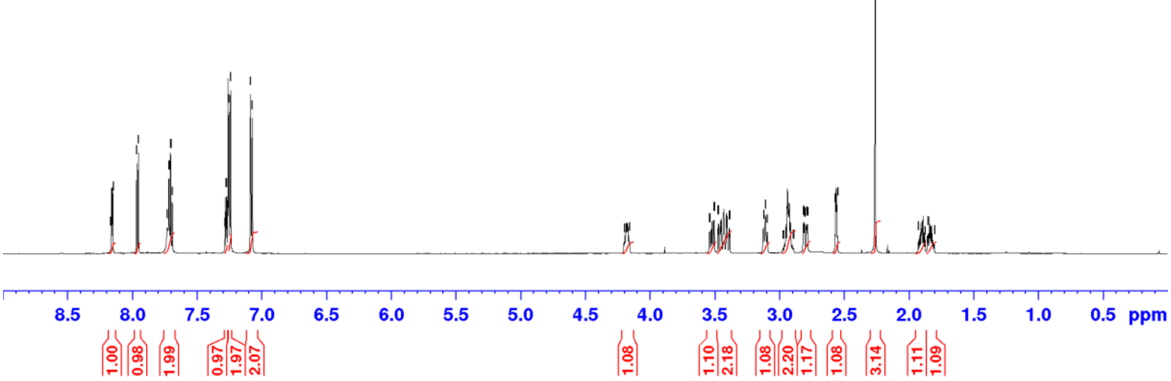
7.97 7.95 7.73 7.72 7.72 7.71 7.70 7.69 7.69 7.28 7.28 7.28 7.27 7.27 7.26 7.25 7.24 7.09 7.07
4.20 4.16 3.54 3.54 3.51 3.50 3.47 3.47 3.39 3.38 3.12 3.11 3.09 2.97 2.97 2.89 2.89 2.82 2.81 2.81 2.80 2.79 2.79 2.78 2.78 2.57 2.55 2.26 1.93 1.88 1.86 1.80
8.5 8.0 7.5 7.0 6.5 6.0 5.5 5.0 4.5 4.0 3.5 3.0 2.5 2.0 1.5 1.0 0.5 ppm
1.00 0.98 1.99 0.97 1.97 2.07 1.08 1.10 2.18 1.08 2.20 1.17 1.08 3.14 1.11 1.09

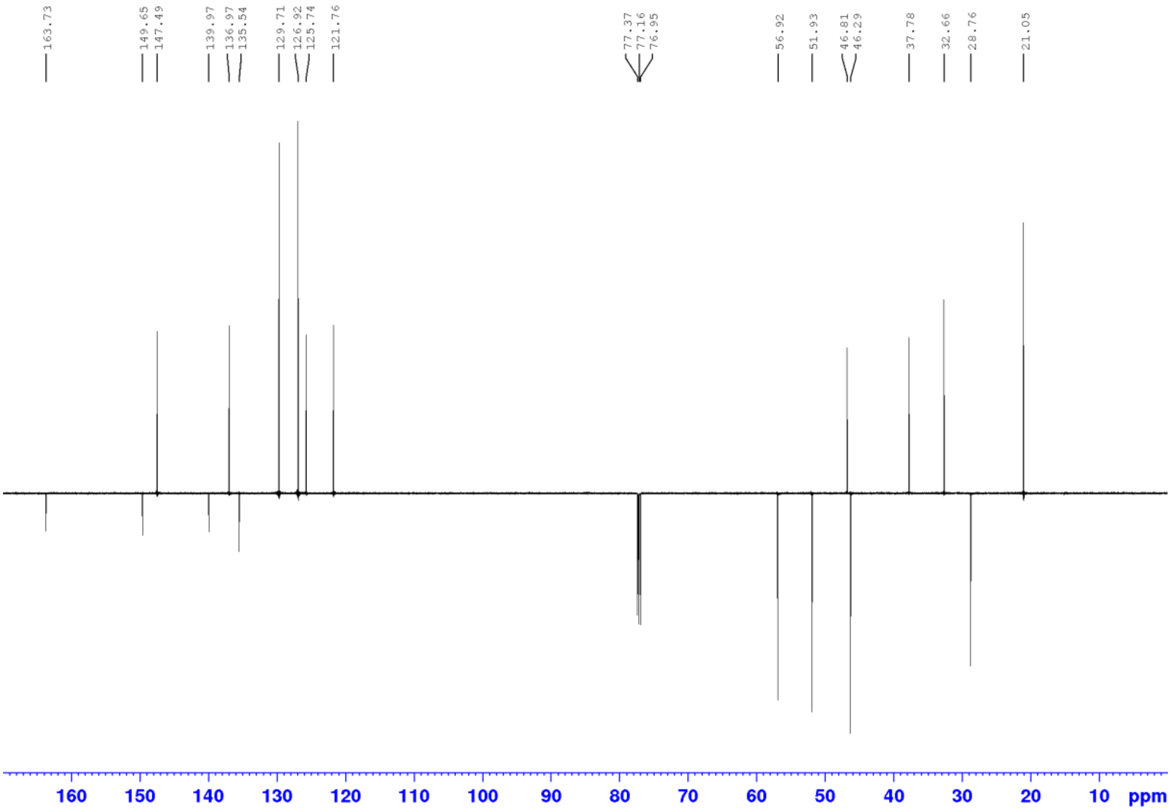
163.73
149.65 147.49
139.97 136.97 135.54
129.71 126.92 125.74
121.76
77.37 77.16 76.95
56.92
51.93
46.81 46.29
37.78
32.66
28.76
21.05
160 150 140 130 120 110 100 90 80 70 60 50 40 30 20 10 ppm

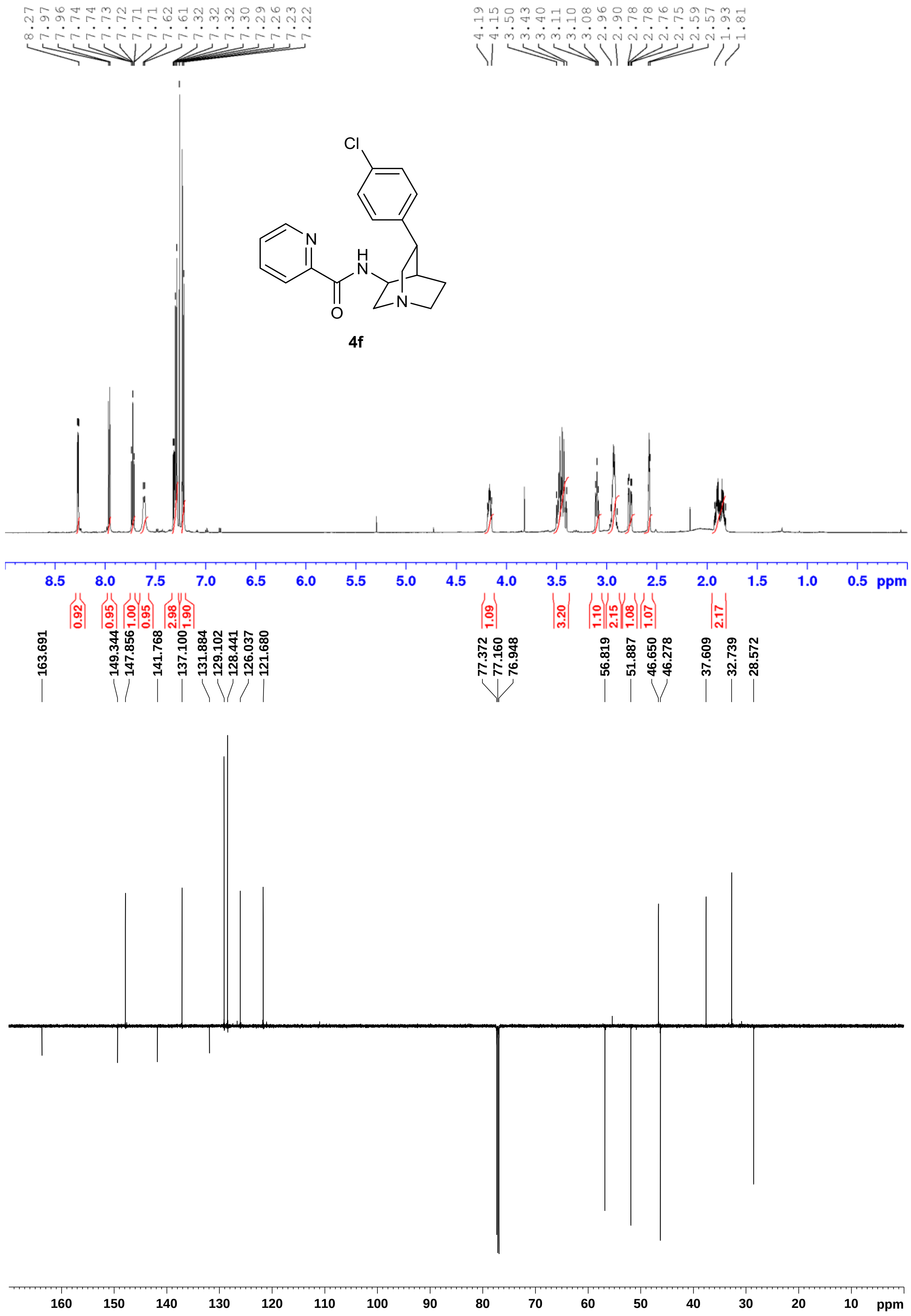

4f

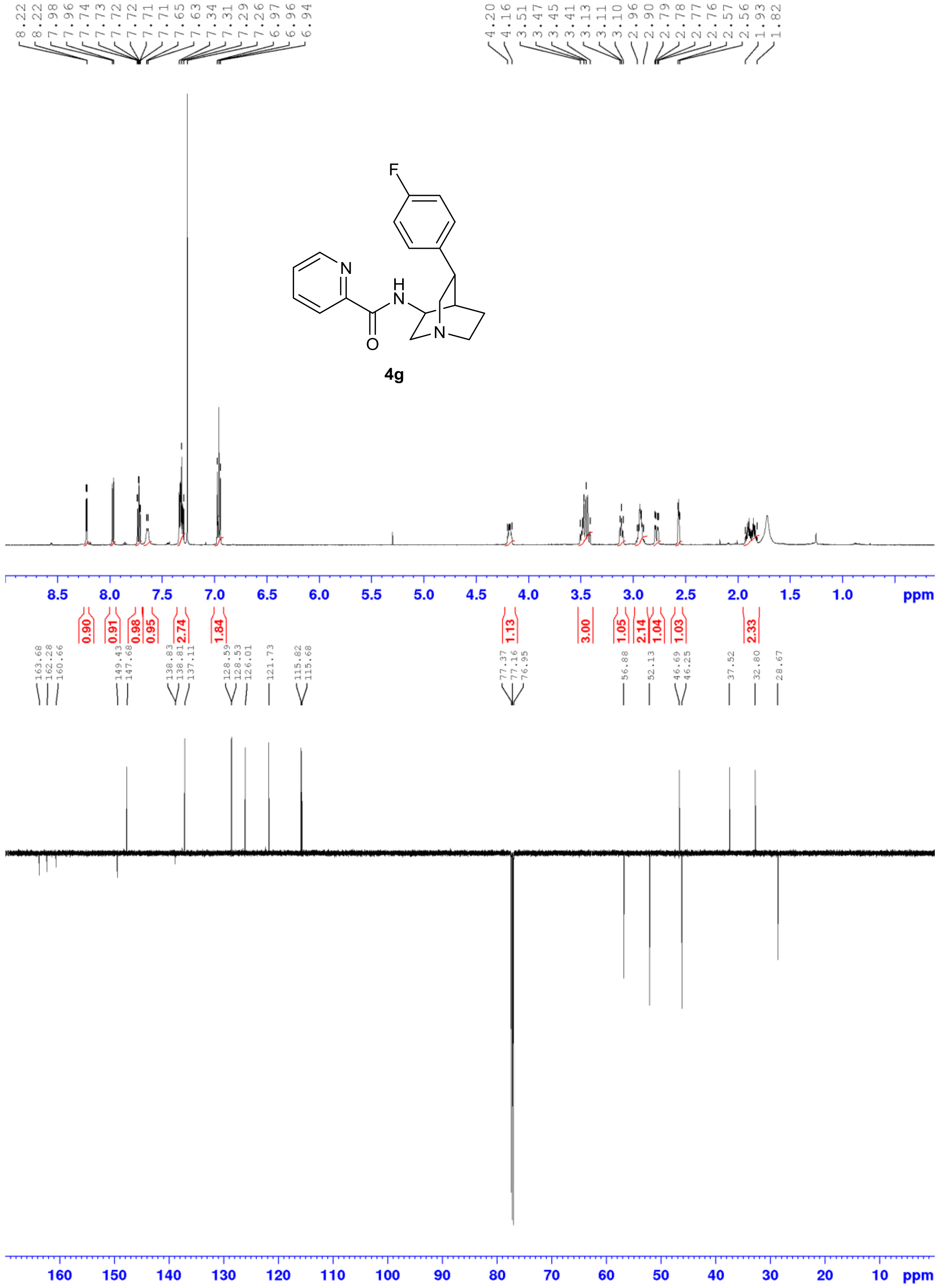
8.22 8.22 7.98 7.96 7.74 7.73 7.72 7.72 7.71 7.71 7.65 7.63 7.34 7.31 7.29 7.26 6.97 6.96 6.94
4.20 4.16 3.51 3.47 3.45 3.41 3.13 3.11 3.10 2.96 2.90 2.79 2.78 2.77 2.76 2.57 2.56 1.93 1.82
F
N
H
N
O
N
4g
8.5 8.0 7.5 7.0 6.5 6.0 5.5 5.0 4.5 4.0 3.5 3.0 2.5 2.0 1.5 1.0 ppm
0.90 0.91 0.98 0.95 2.74 1.84 1.13 3.00 1.05 2.14 1.04 1.03 2.33
163.68 162.28 160.66 149.43 147.68 138.83 138.81 137.11 128.59 128.53 126.01 121.73 115.82 115.68
77.37 77.16 76.95 56.88 52.13 46.69 46.25 37.52 32.80 28.67
160 150 140 130 120 110 100 90 80 70 60 50 40 30 20 10 ppm

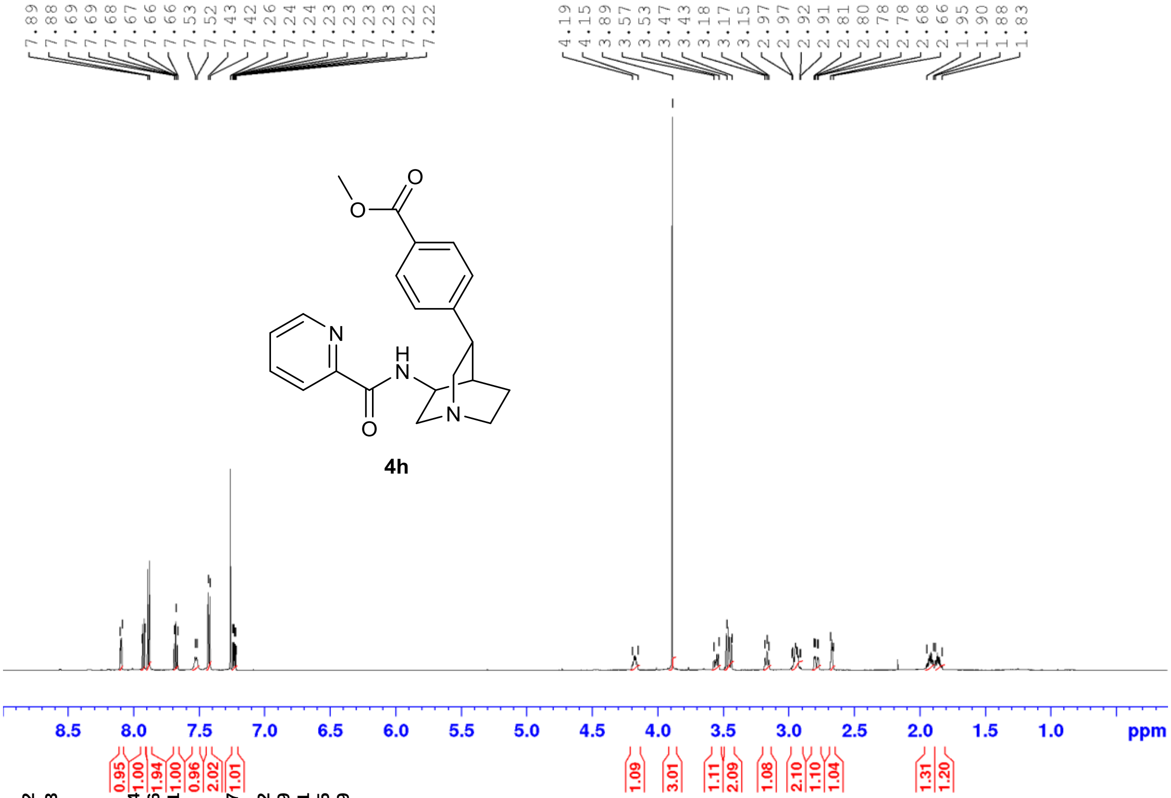

7.89 7.88 7.69 7.69 7.68 7.67 7.66 7.53 7.52 7.43 7.42 7.26 7.24 7.24 7.23 7.23 7.23 7.23 7.22 7.22
4.19 4.15 3.89 3.57 3.53 3.47 3.43 3.18 3.17 3.15 2.97 2.97 2.92 2.91 2.81 2.80 2.78 2.78 2.68 2.66 1.95 1.90 1.88 1.83
O
O
N
H
N
O
N
4h
8.5 8.0 7.5 7.0 6.5 6.0 5.5 5.0 4.5 4.0 3.5 3.0 2.5 2.0 1.5 1.0 ppm
0.95 1.00 1.94 1.00 0.96 2.02 1.01 1.09 3.01 1.11 2.09 1.08 2.10 1.10 1.04 1.31 1.20

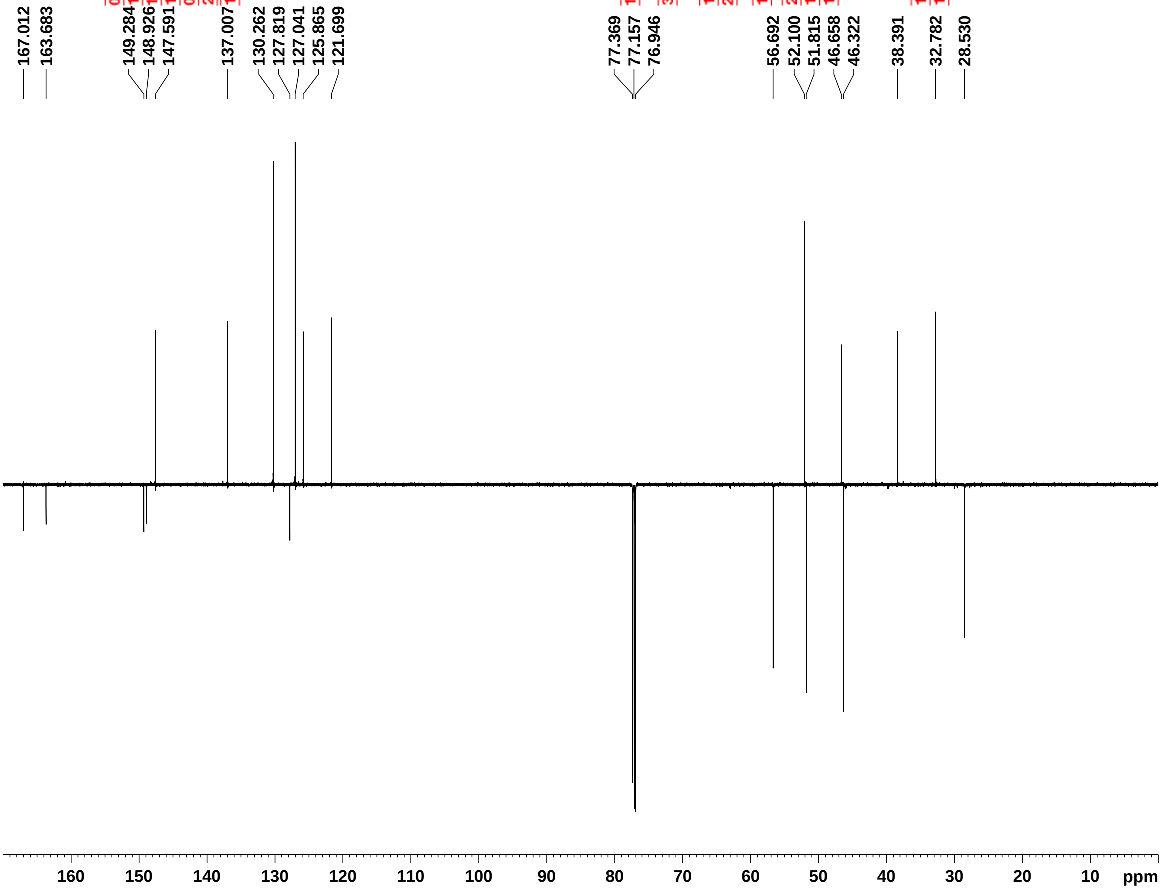

167.012 163.683 149.284 148.926 147.591 137.007 130.262 127.819 127.041 125.865 121.699 77.369 77.157 76.946 56.692 52.100 51.815 46.658 46.322 38.391 32.782 28.530
160 150 140 130 120 110 100 90 80 70 60 50 40 30 20 10 ppm

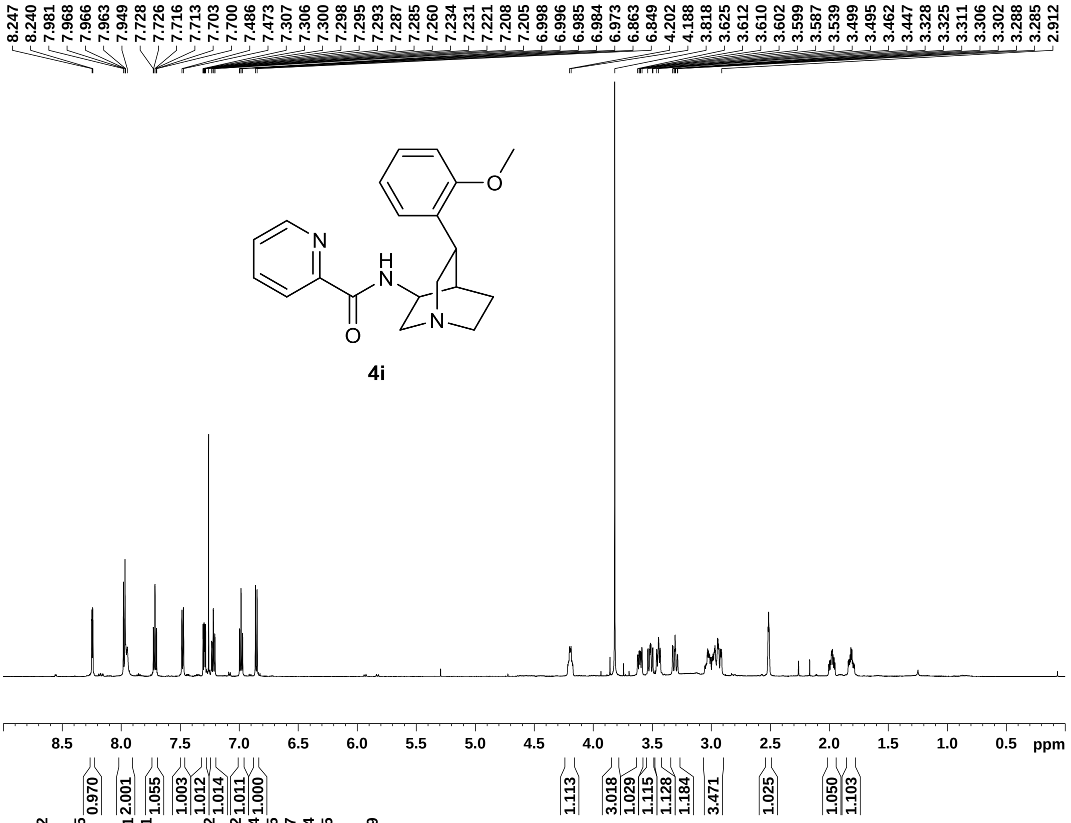
8.247 8.240 7.981 7.968 7.966 7.963 7.949 7.728 7.726 7.716 7.713 7.703 7.700 7.486 7.473 7.307 7.306 7.300 7.298 7.295 7.293 7.287 7.285 7.260 7.234 7.231 7.221 7.208 7.205 6.998 6.996 6.985 6.984 6.973 6.863 6.849 4.202 4.188 3.818 3.625 3.612 3.610 3.602 3.599 3.587 3.539 3.499 3.495 3.462 3.447 3.328 3.325 3.311 3.306 3.302 3.288 3.285 2.912
N
H
N
O
N
O
4i
8.5 8.0 7.5 7.0 6.5 6.0 5.5 5.0 4.5 4.0 3.5 3.0 2.5 2.0 1.5 1.0 0.5 ppm
0.970 2.001 1.055 1.003 1.012 1.014 1.011 1.000 1.113 3.018 1.029 1.115 1.128 1.184 3.471 1.025 1.050 1.103

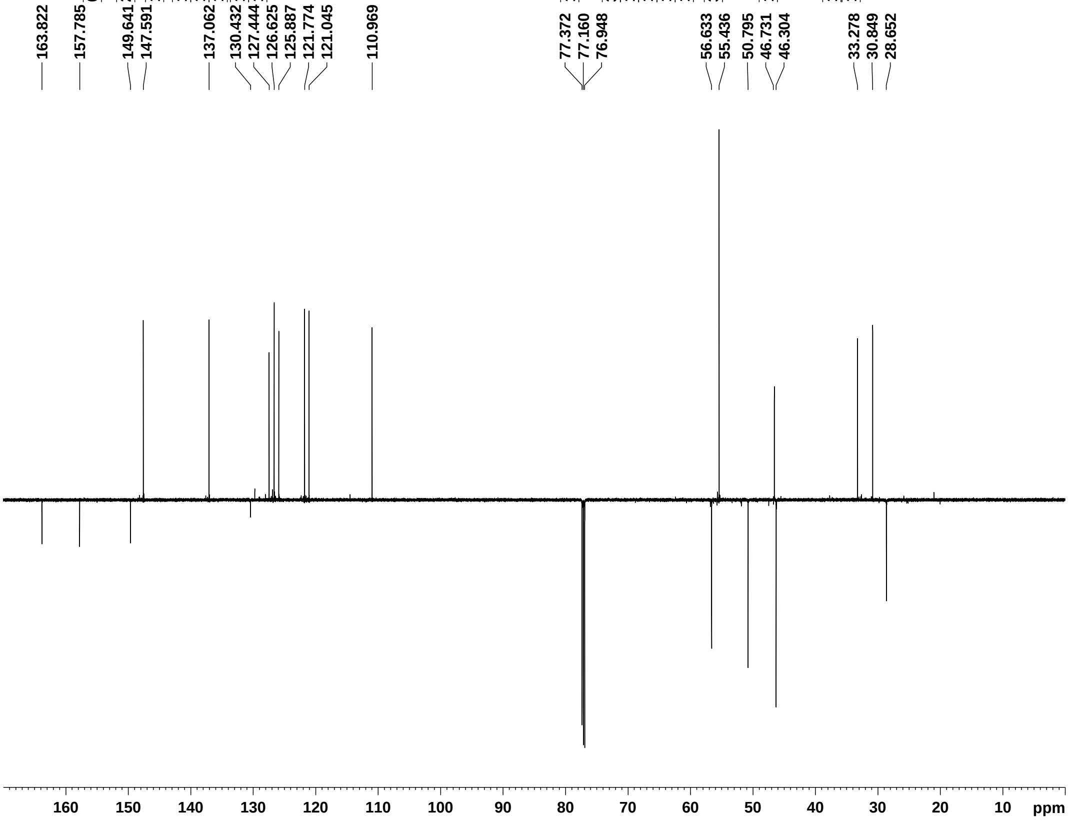
163.822 157.785 149.641 147.591 137.062 130.432 127.444 126.625 125.887 121.774 121.045 110.969 77.372 77.160 76.948 56.633 55.436 50.795 46.731 46.304 33.278 30.849 28.652
160 150 140 130 120 110 100 90 80 70 60 50 40 30 20 10 ppm

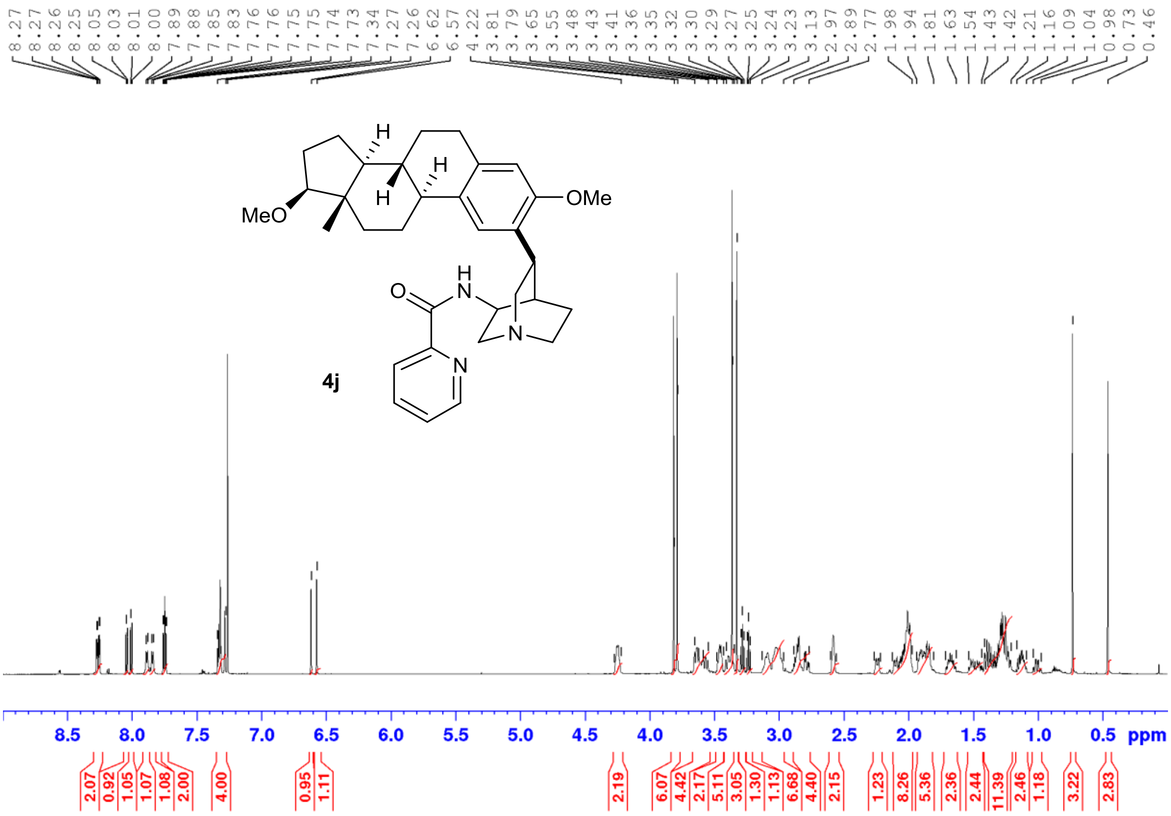
MeO
OMe
H
O
N
4j
8.5 8.0 7.5 7.0 6.5 6.0 5.5 5.0 4.5 4.0 3.5 3.0 2.5 2.0 1.5 1.0 0.5 ppm

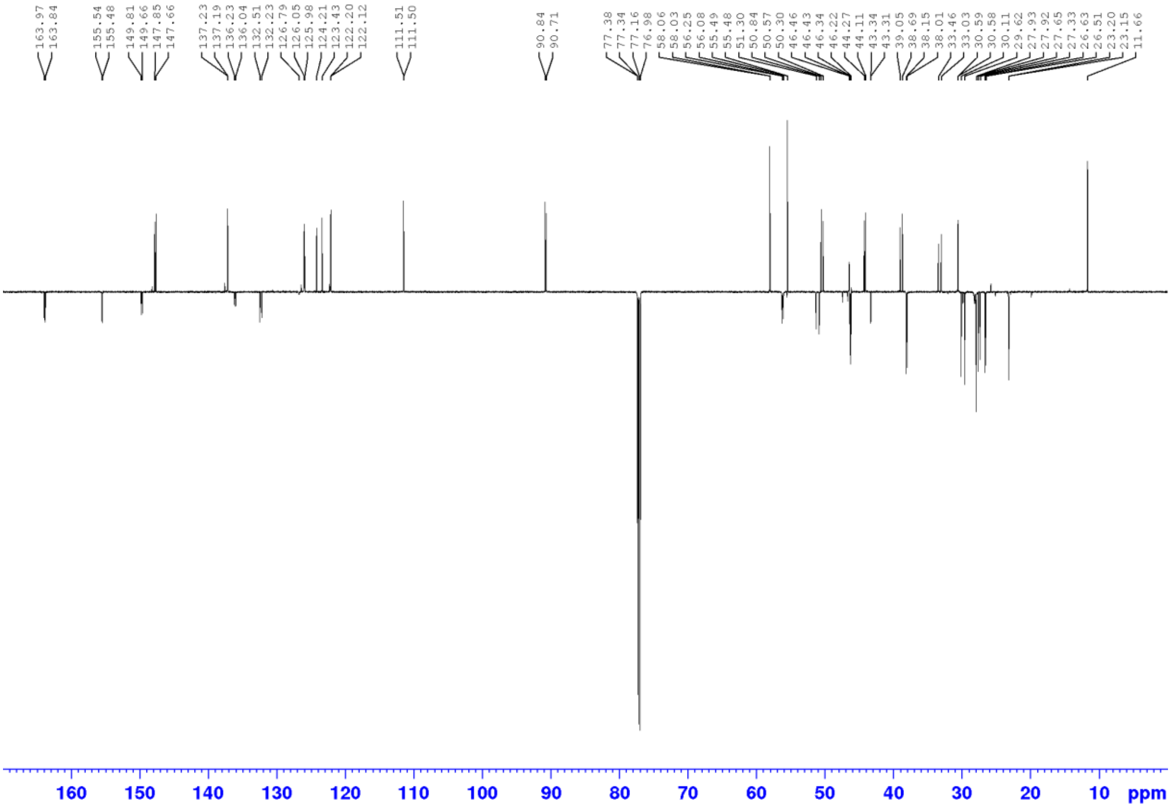
160 150 140 130 120 110 100 90 80 70 60 50 40 30 20 10 ppm

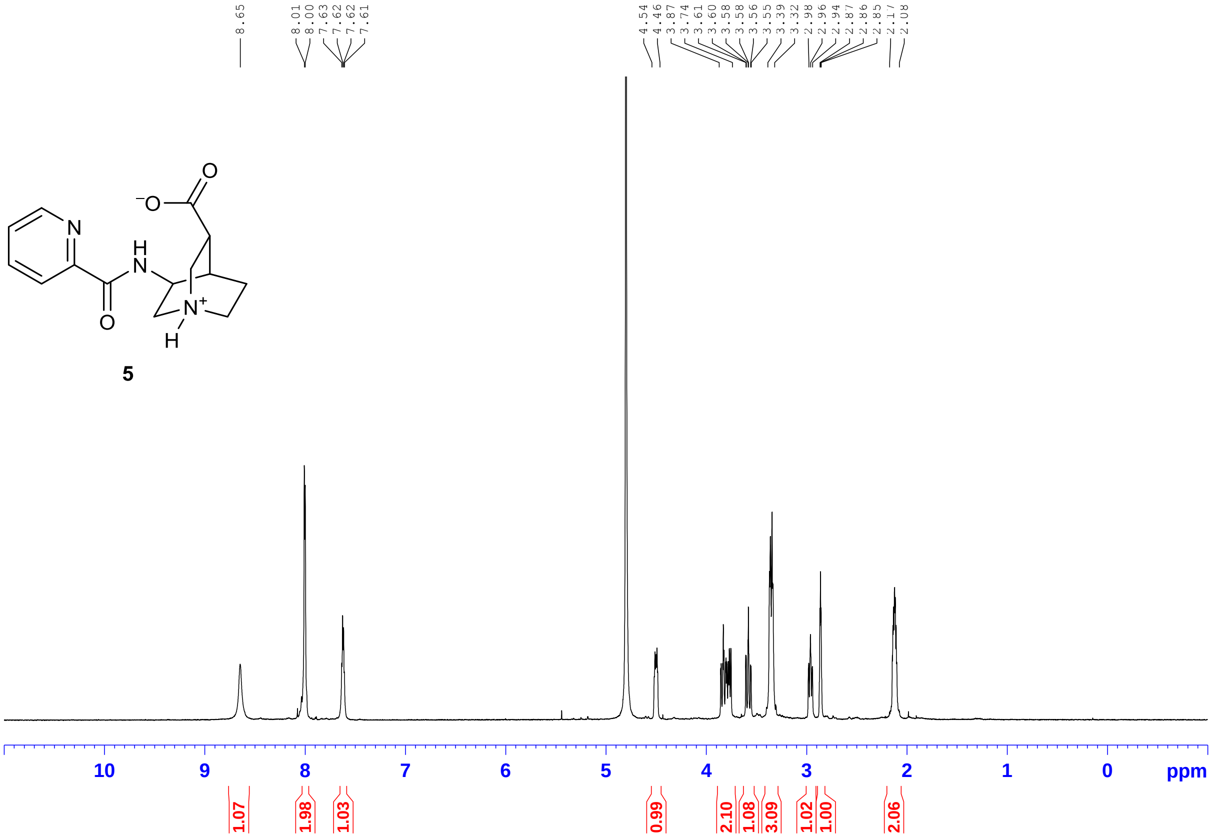

O
−O
N
H
N
O
N+
H
8.65
8.01
8.00
7.63
7.62
7.62
7.61
4.54
4.46
3.87
3.74
3.61
3.60
3.58
3.58
3.56
3.55
3.39
3.32
2.98
2.96
2.94
2.87
2.86
2.85
2.17
2.08
10
9
8
7
6
5
4
3
2
1
0
ppm
1.07
1.98
1.03
0.99
2.10
1.08
3.09
1.02
1.00
2.06

5

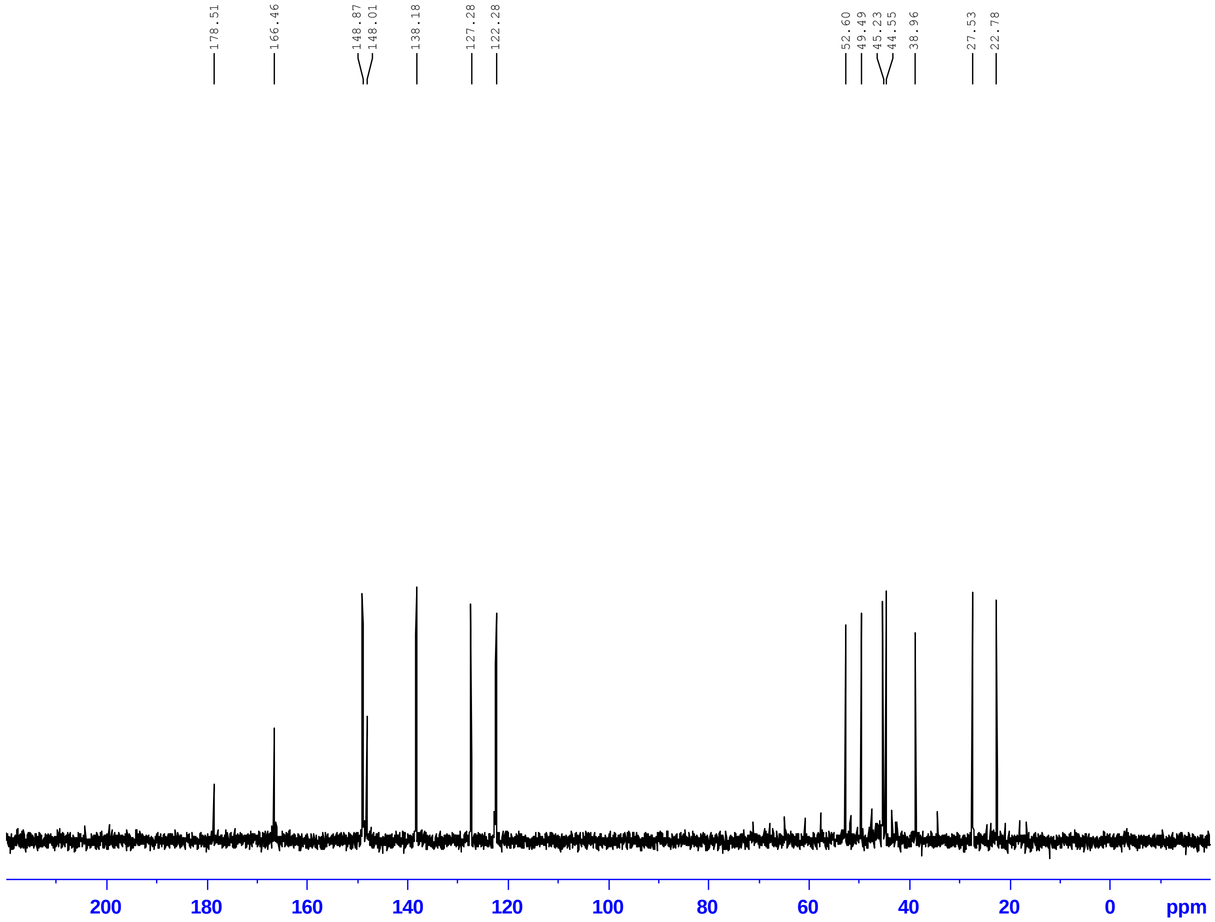

178.51
166.46
148.87
148.01
138.18
127.28
122.28
52.60
49.49
45.23
44.55
38.96
27.53
22.78
200
180
160
140
120
100
80
60
40
20
0
ppm

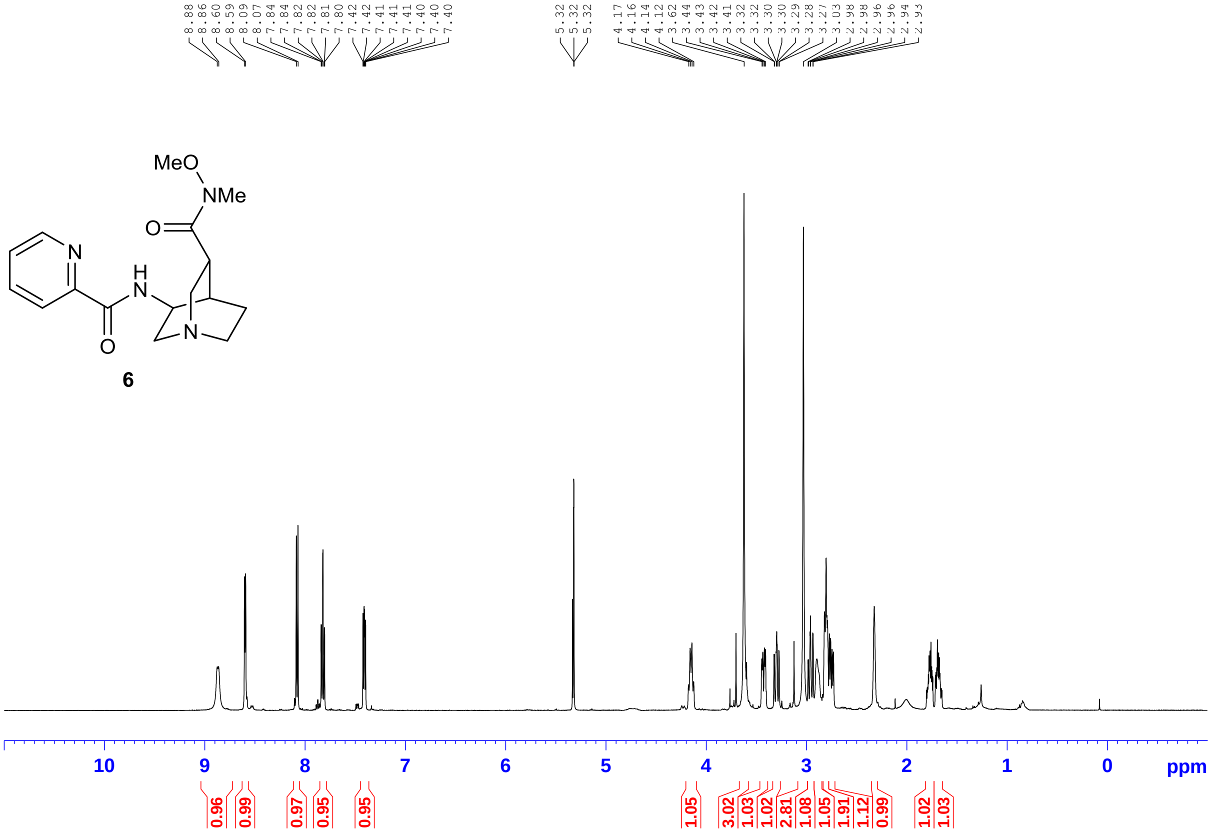
8.88
8.86
8.60
8.59
8.09
8.07
7.84
7.84
7.82
7.82
7.81
7.80
7.42
7.42
7.41
7.41
7.41
7.40
7.40
7.40
5.32
5.32
5.32
4.17
4.16
4.14
4.12
3.62
3.44
3.43
3.42
3.41
3.32
3.32
3.30
3.30
3.29
3.28
3.27
3.03
2.98
2.98
2.96
2.94
2.93
MeO
NMe
O
N
H
N
N
O
6
10 9 8 7 6 5 4 3 2 1 0 ppm
0.96
0.99
0.97
0.95
0.95
1.05
3.02
1.03
1.02
2.81
1.08
1.05
1.91
1.12
0.99
1.02
1.03

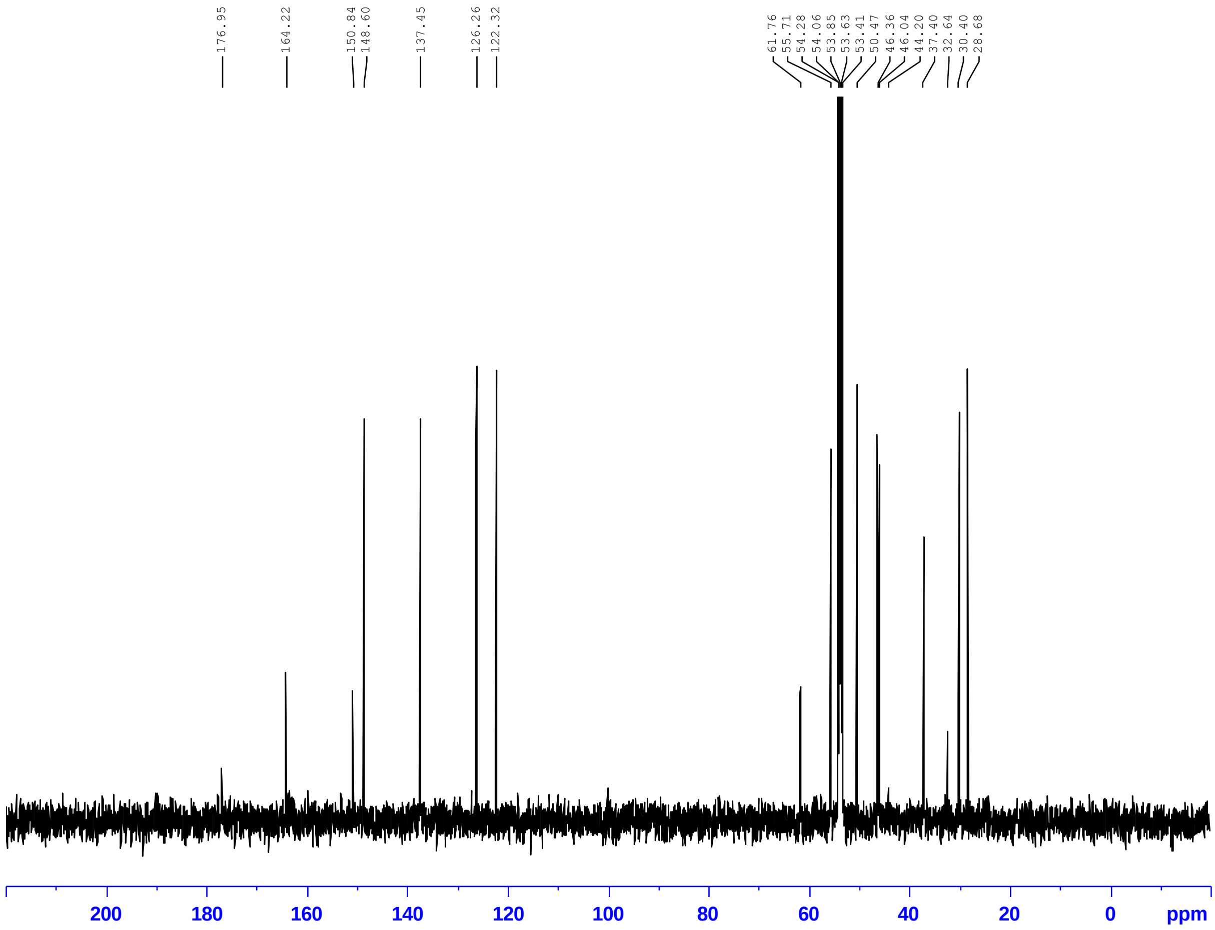
176.95
164.22
150.84
148.60
137.45
126.26
122.32
61.76
55.71
54.28
54.06
53.85
53.63
53.41
50.47
46.36
46.04
44.20
37.40
32.64
30.40
28.68
200 180 160 140 120 100 80 60 40 20 0 ppm

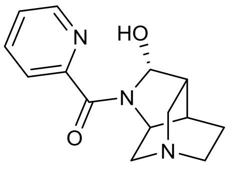
HO
N
O
N
N

7

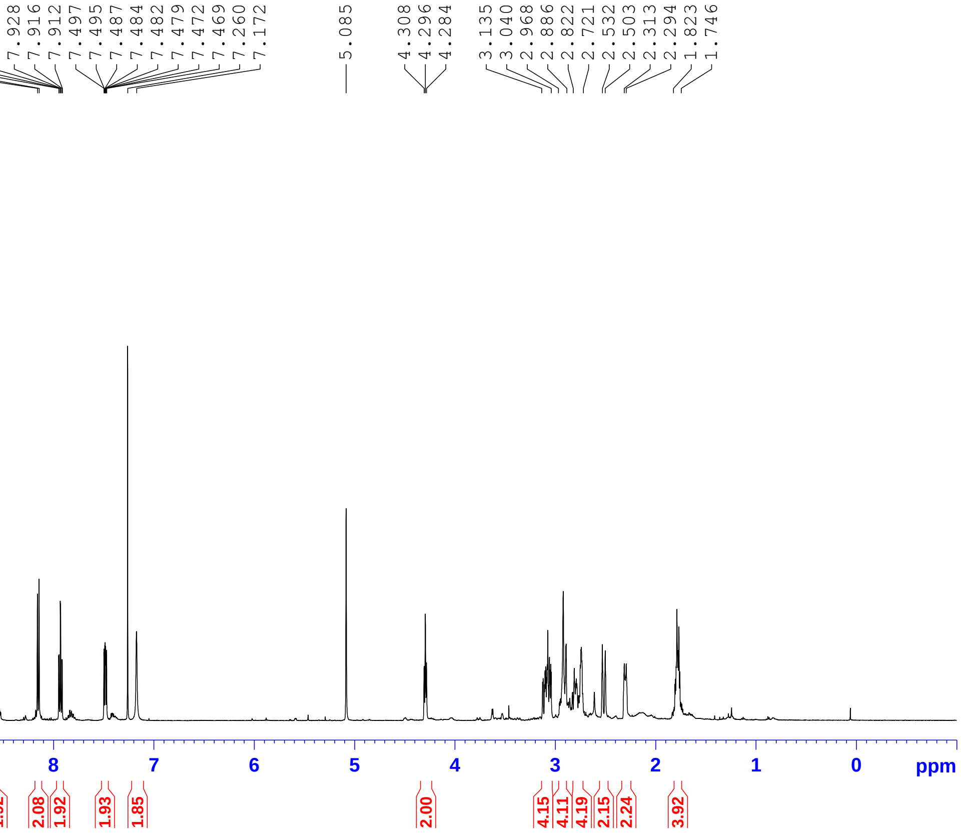
8.159 8.143 7.947 7.943 7.931 7.928 7.916 7.912 7.497 7.495 7.487 7.484 7.482 7.479 7.472 7.469 7.260 7.172
5.085
4.308 4.296 4.284
3.135 3.040 2.968 2.886 2.822 2.721 2.532 2.503 2.313 2.294 1.823 1.746
10 9 8 7 6 5 4 3 2 1 0 ppm
1.92 2.08 1.92 1.93 1.85 2.00 4.15 4.11 4.19 2.15 2.24 3.92

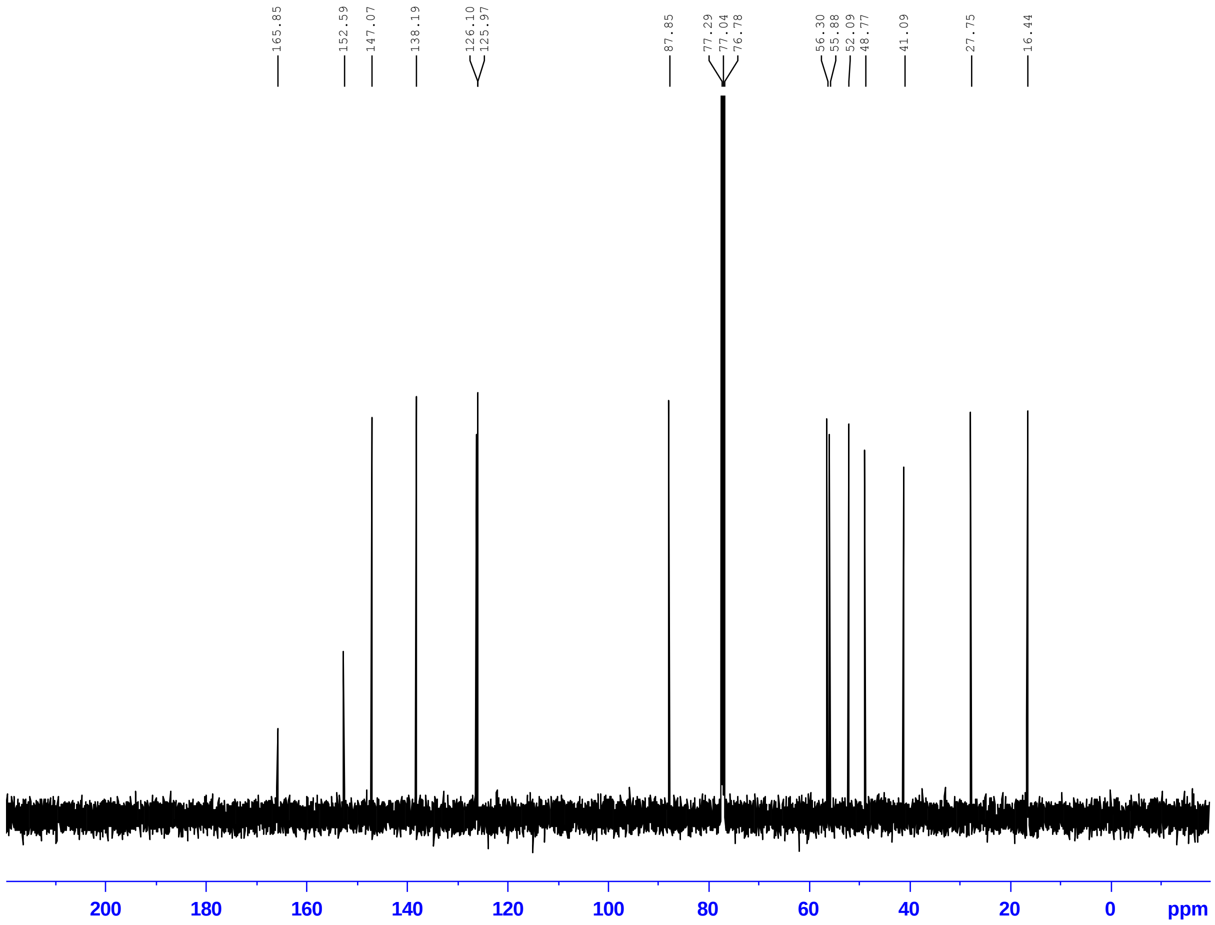
165.85
152.59
147.07
138.19
126.10 125.97
87.85
77.29 77.04 76.78
56.30 55.88 52.09 48.77
41.09
27.75
16.44
200 180 160 140 120 100 80 60 40 20 0 ppm

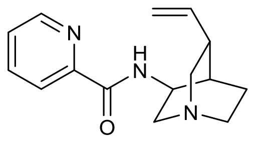
N
H
N
O
N

8

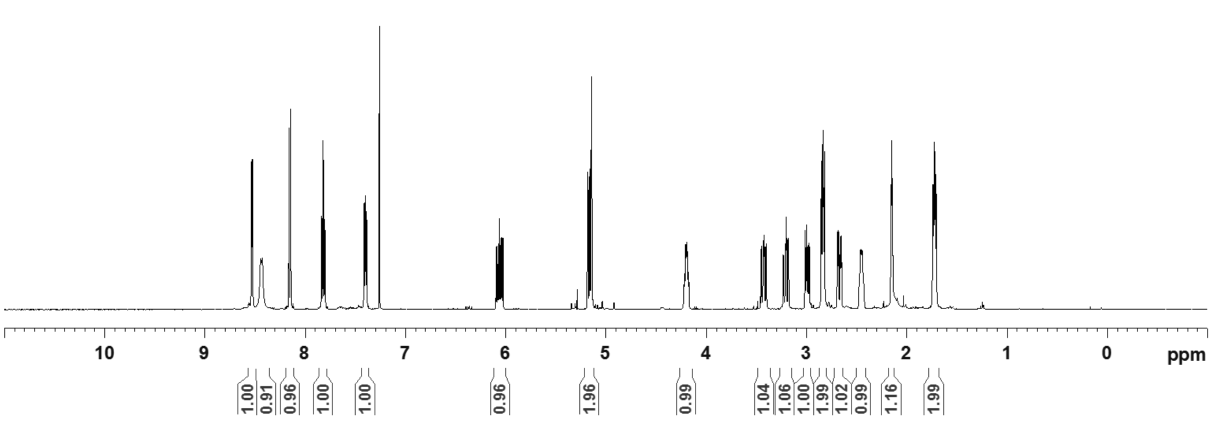
8.54 8.54 8.53 8.53 8.44 8.43 8.16 8.15 7.84 7.84 7.82 7.82 7.81 7.80 7.41 7.41 7.40 7.40 7.40 7.40 7.39 7.26 6.10 6.09 6.08 6.06 6.06 6.05 6.04 6.03 5.19 5.17 5.15 4.24 4.17 3.45 3.45 3.43 3.43 3.42 3.41 3.40 3.23 3.21 3.20 3.18 3.01 3.00 2.99 2.97 2.85 2.84 2.82 2.69 2.68 2.66 2.65 2.49 2.42 2.17 2.13 1.75 1.70
10
9
8
7
6
5
4
3
2
1
0
ppm
1.00 0.91 0.96 1.00 1.00 0.96 1.96 0.99 1.04 1.06 1.00 1.99 1.02 0.99 1.16 1.99

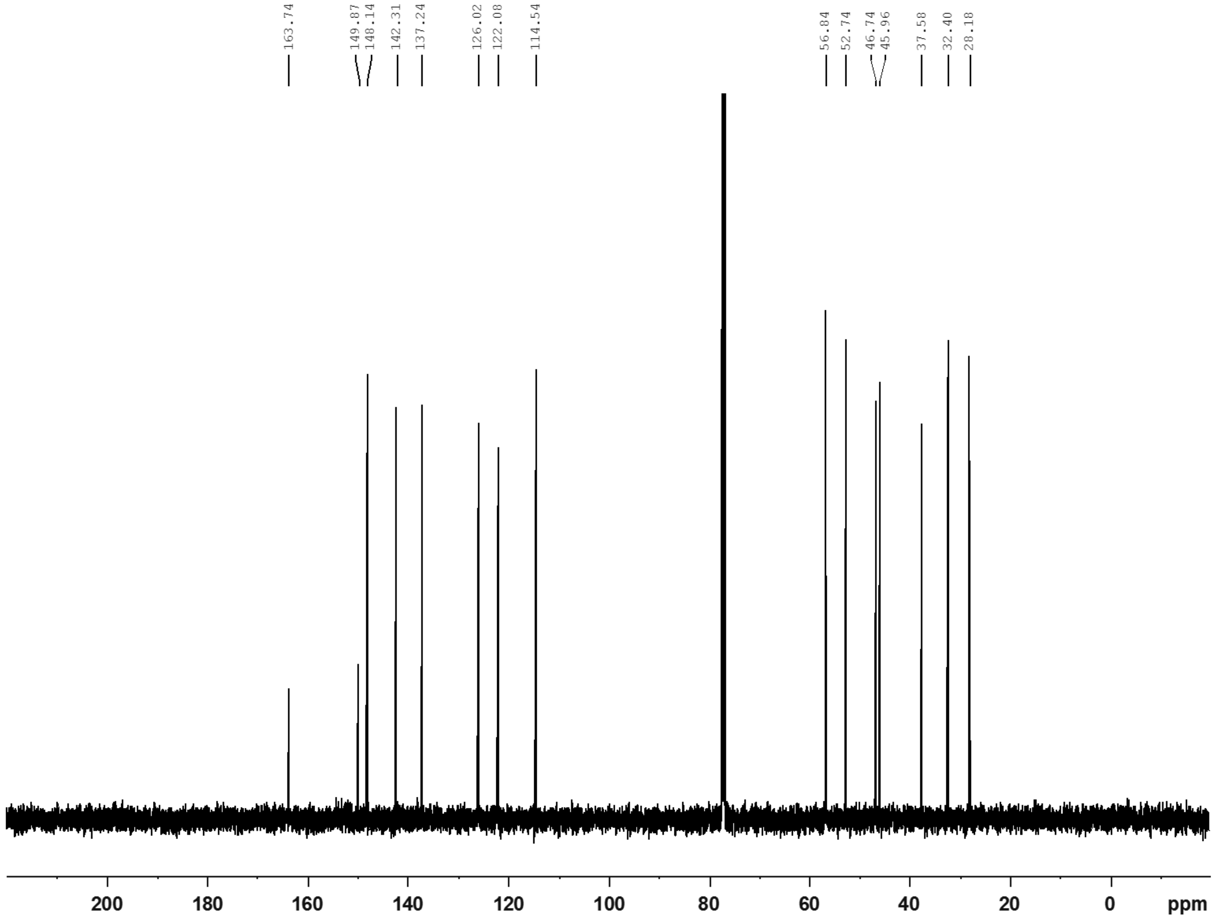
163.74
149.87 148.14
142.31
137.24
126.02
122.08
114.54
56.84
52.74
46.74 45.96
37.58
32.40
28.18
200
180
160
140
120
100
80
60
40
20
0
ppm

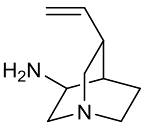
H2N
N

9

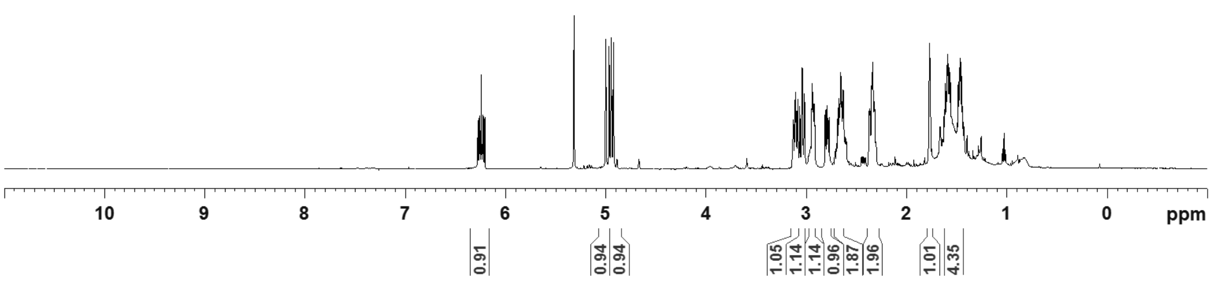
10 9 8 7 6 5 4 3 2 1 0 ppm
0.91
0.94
0.94
1.05
1.14
1.14
0.96
1.87
1.96
1.01
4.35

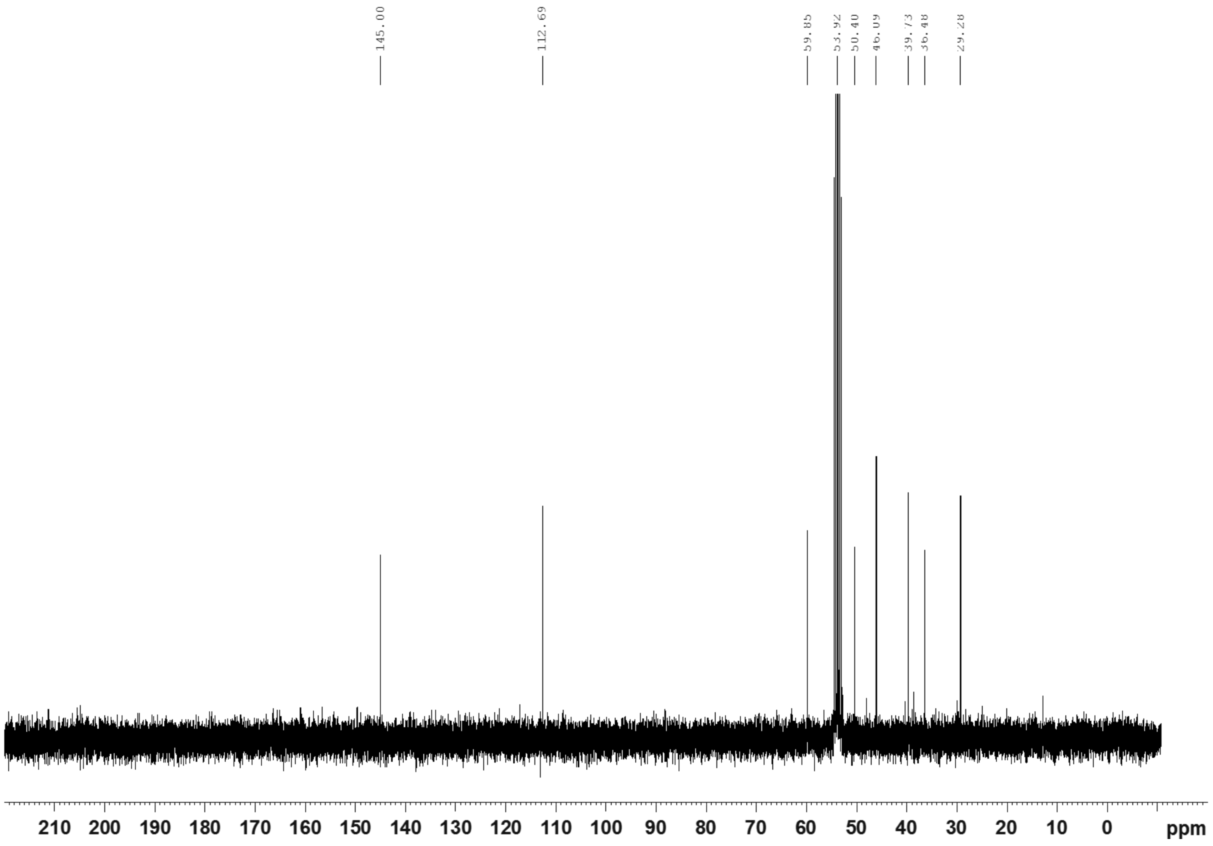
145.00
112.69
59.85
53.92
50.40
46.09
39.73
36.48
29.28
210 200 190 180 170 160 150 140 130 120 110 100 90 80 70 60 50 40 30 20 10 0 ppm

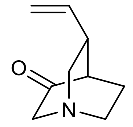

10

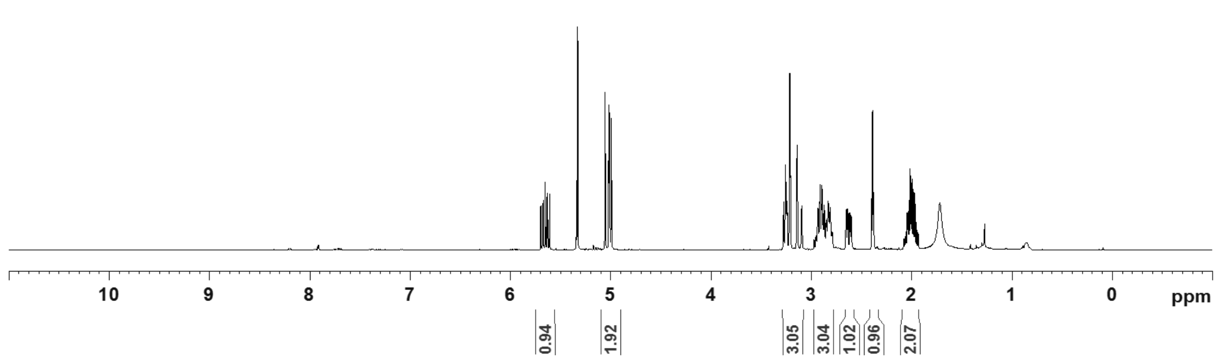

5.70
5.68
5.67
5.65
5.64
5.63
5.61
5.06
5.05
5.05
5.02
5.02
5.02
5.01
5.01
5.00
4.99
4.99
3.30
3.08
2.99
2.78
2.66
2.65
2.64
2.63
2.62
2.62
2.61
2.60
2.40
2.39
2.38
2.09
1.93
10
9
8
7
6
5
4
3
2
1
0
ppm
0.94
1.92
3.05
3.04
1.02
0.96
2.07

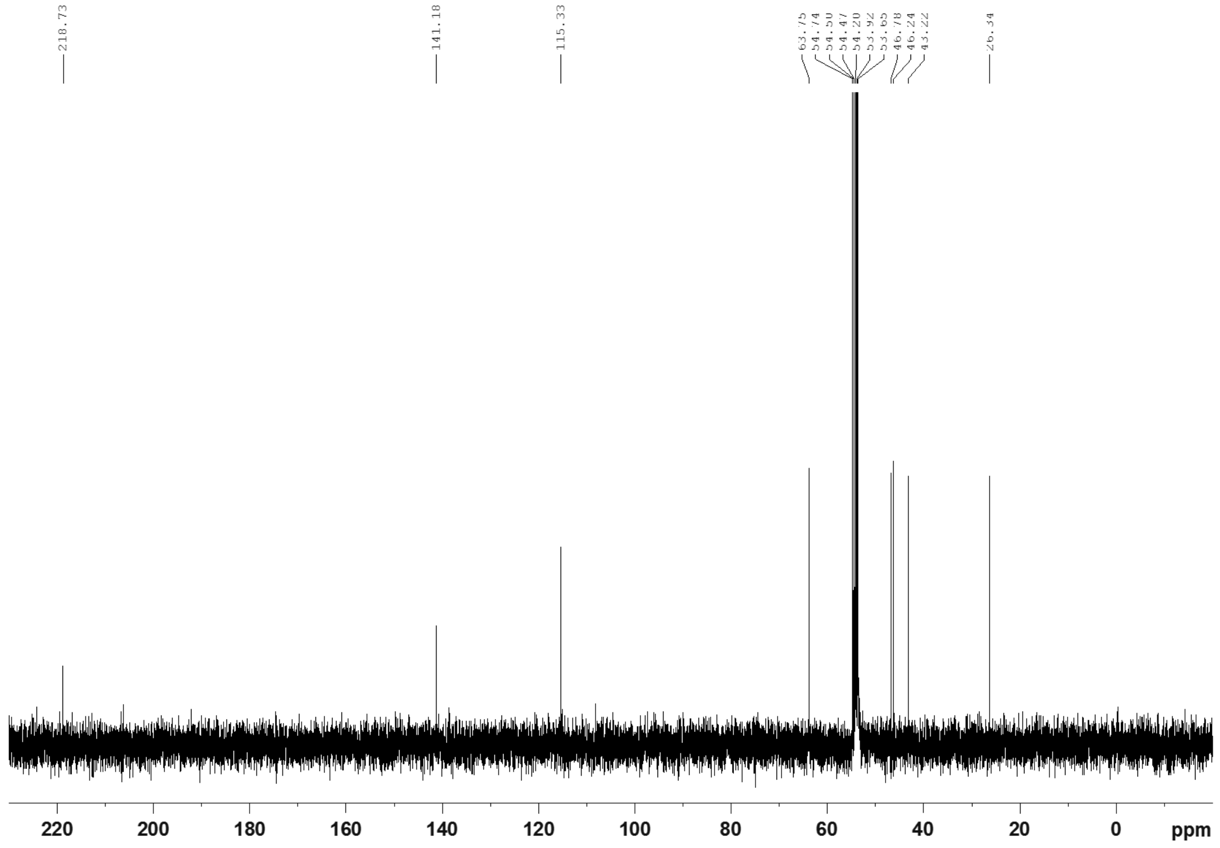

218.73
141.18
115.33
63.75
54.74
54.50
54.47
54.20
53.92
53.65
46.78
46.24
43.22
26.34
220
200
180
160
140
120
100
80
60
40
20
0
ppm

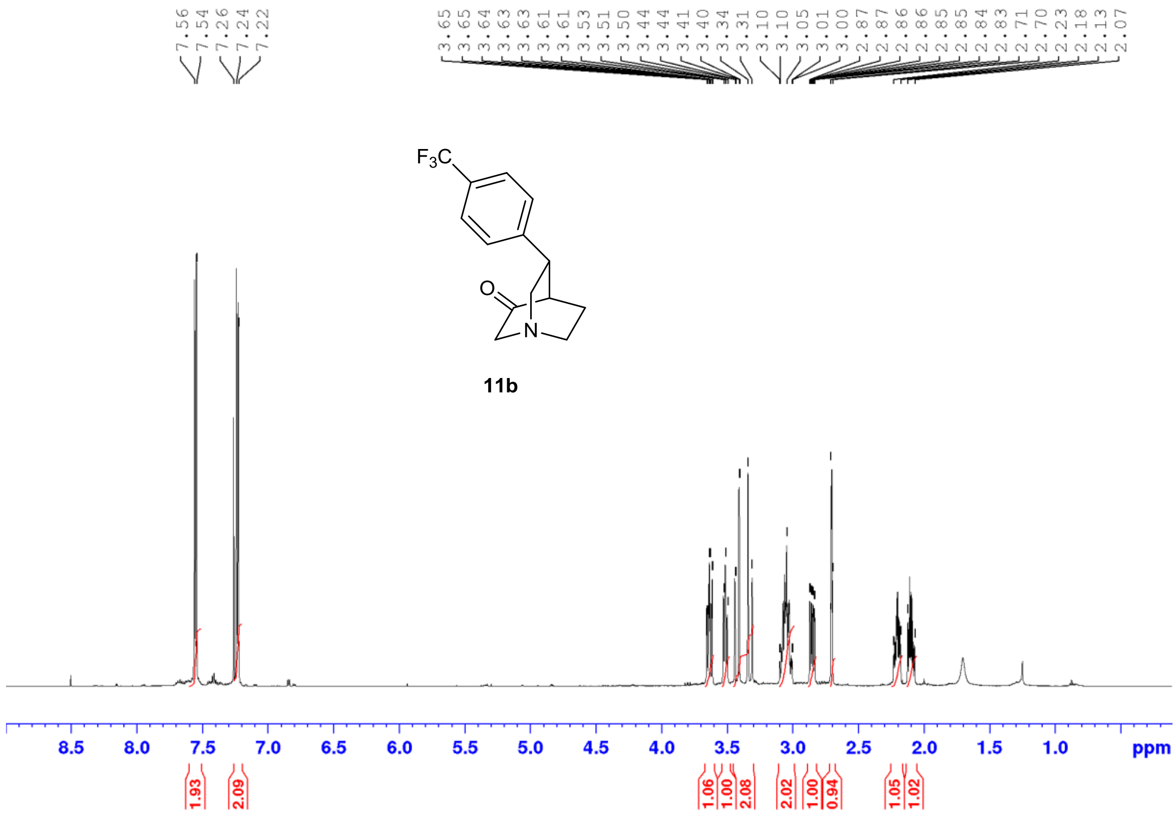

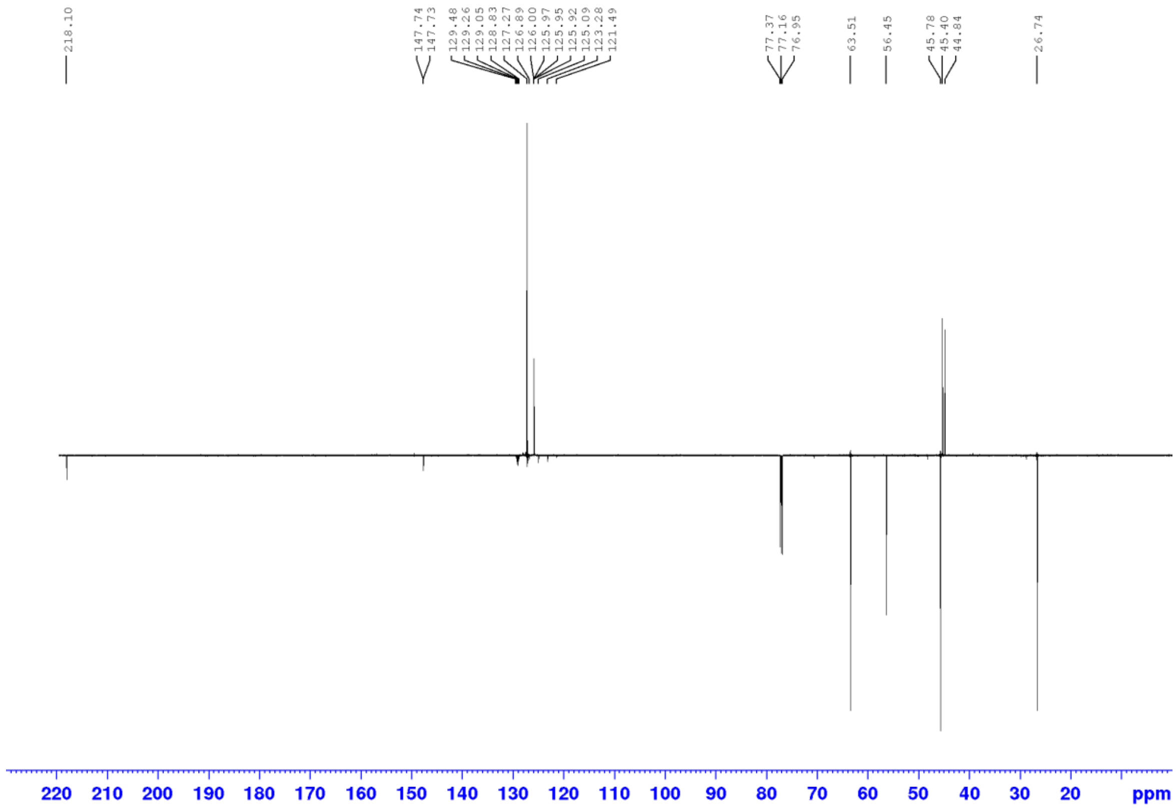

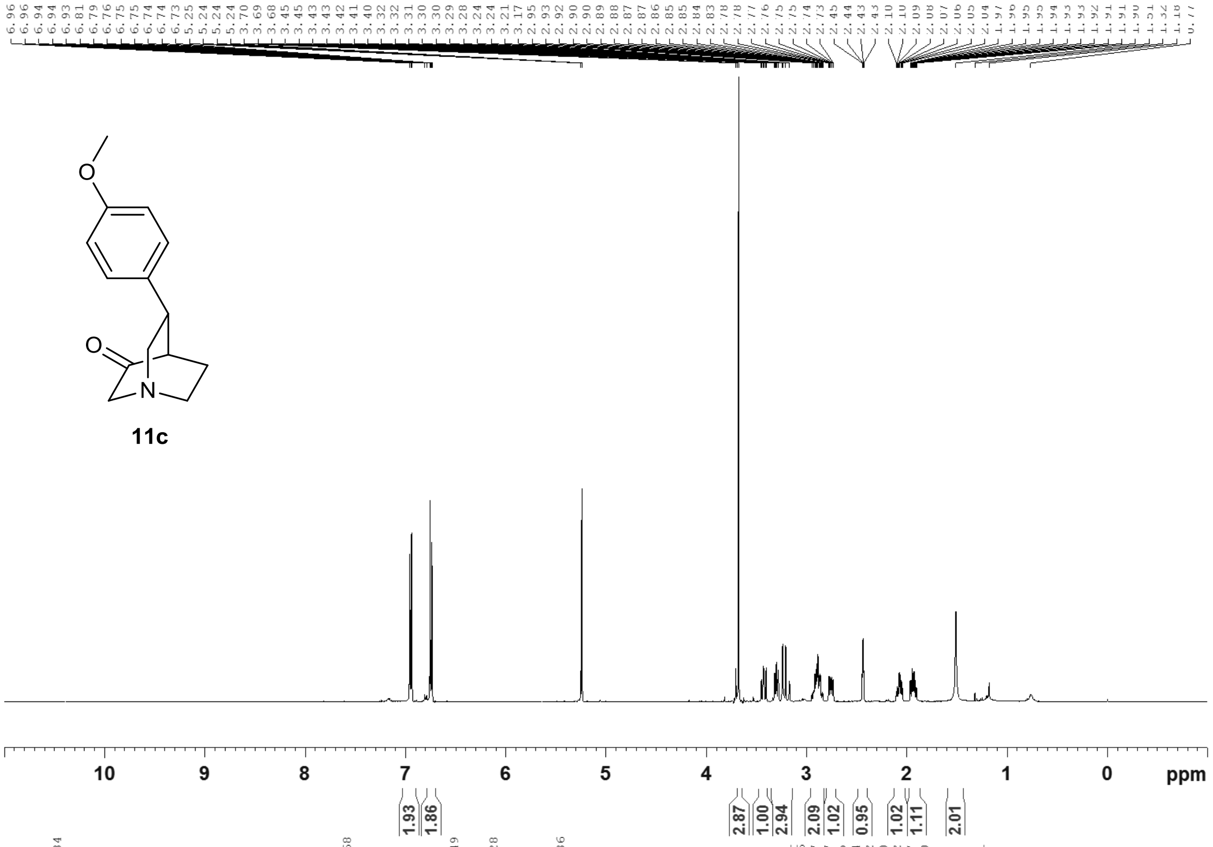
11c
10 9 8 7 6 5 4 3 2 1 0 ppm
1.93 1.86 2.87 1.00 2.94 2.09 1.02 0.95 1.02 1.11 2.01

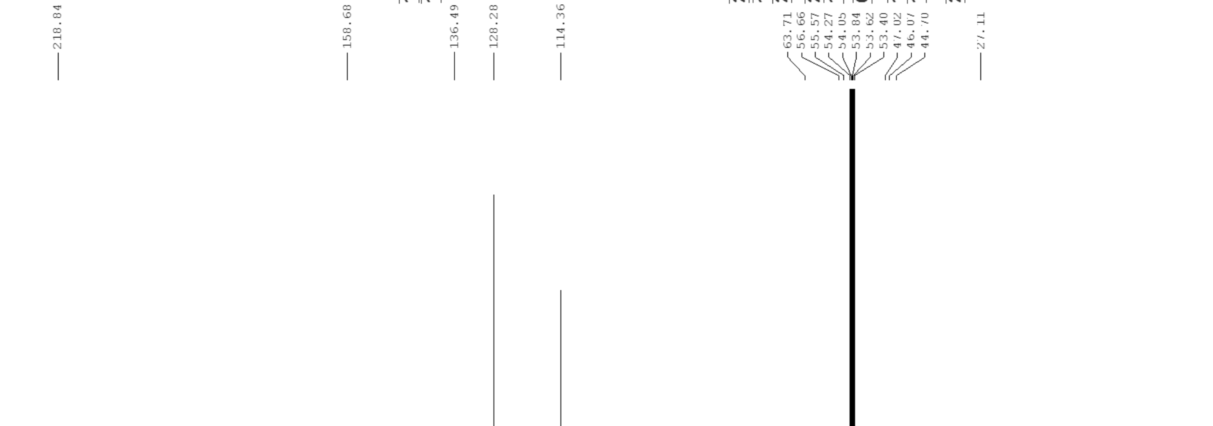
218.84
158.68
136.49
128.28
114.36
63.71
56.66
55.57
54.27
54.05
53.84
53.62
53.40
47.02
46.07
44.70
27.11
220 200 180 160 140 120 100 80 60 40 20 0 ppm

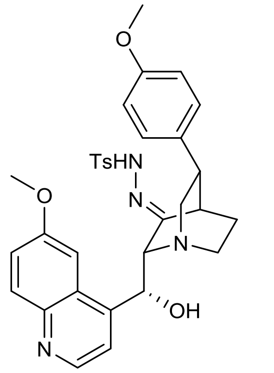
TsHN
N
N
O
O
OH
N

13

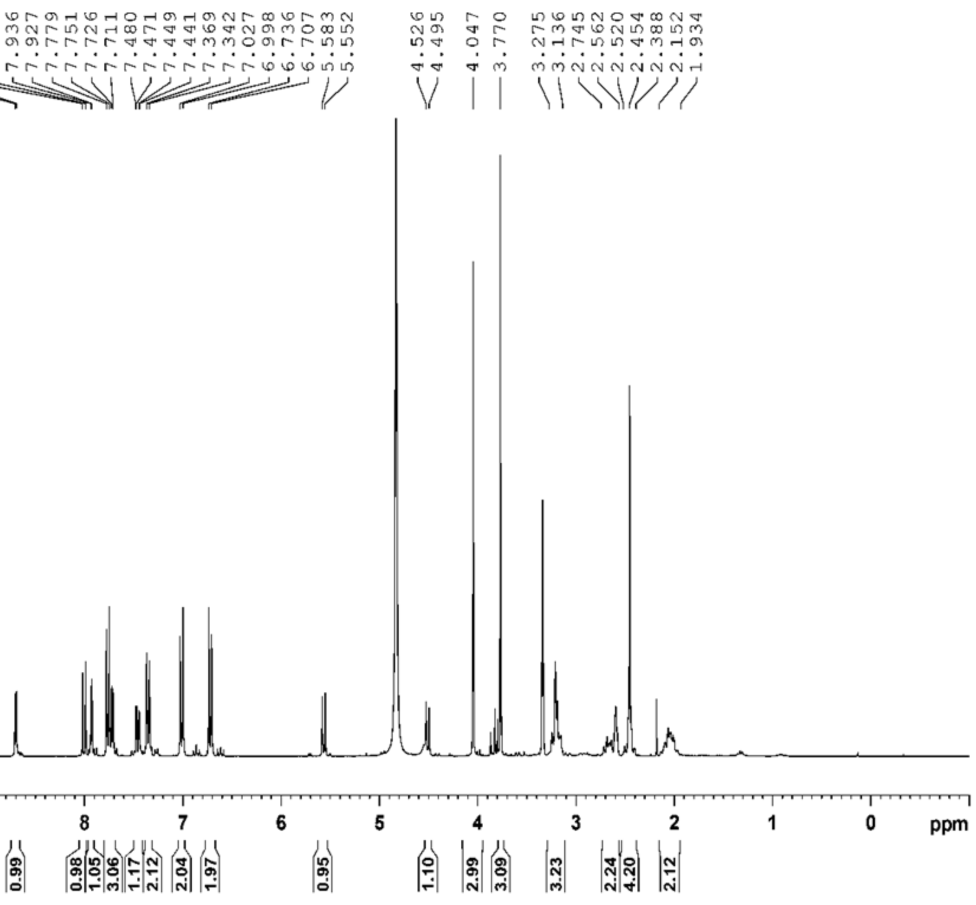
8.709
8.694
8.022
7.991
7.936
7.927
7.779
7.751
7.726
7.711
7.480
7.471
7.449
7.441
7.369
7.342
7.027
6.998
6.736
6.707
5.583
5.552
4.526
4.495
4.047
3.770
3.275
3.136
2.745
2.562
2.520
2.454
2.388
2.152
1.934
10
9
8
7
6
5
4
3
2
1
0
ppm
0.99
0.98
1.05
3.06
1.17
2.12
2.04
1.97
0.95
1.10
2.99
3.09
3.23
2.24
4.20
2.12

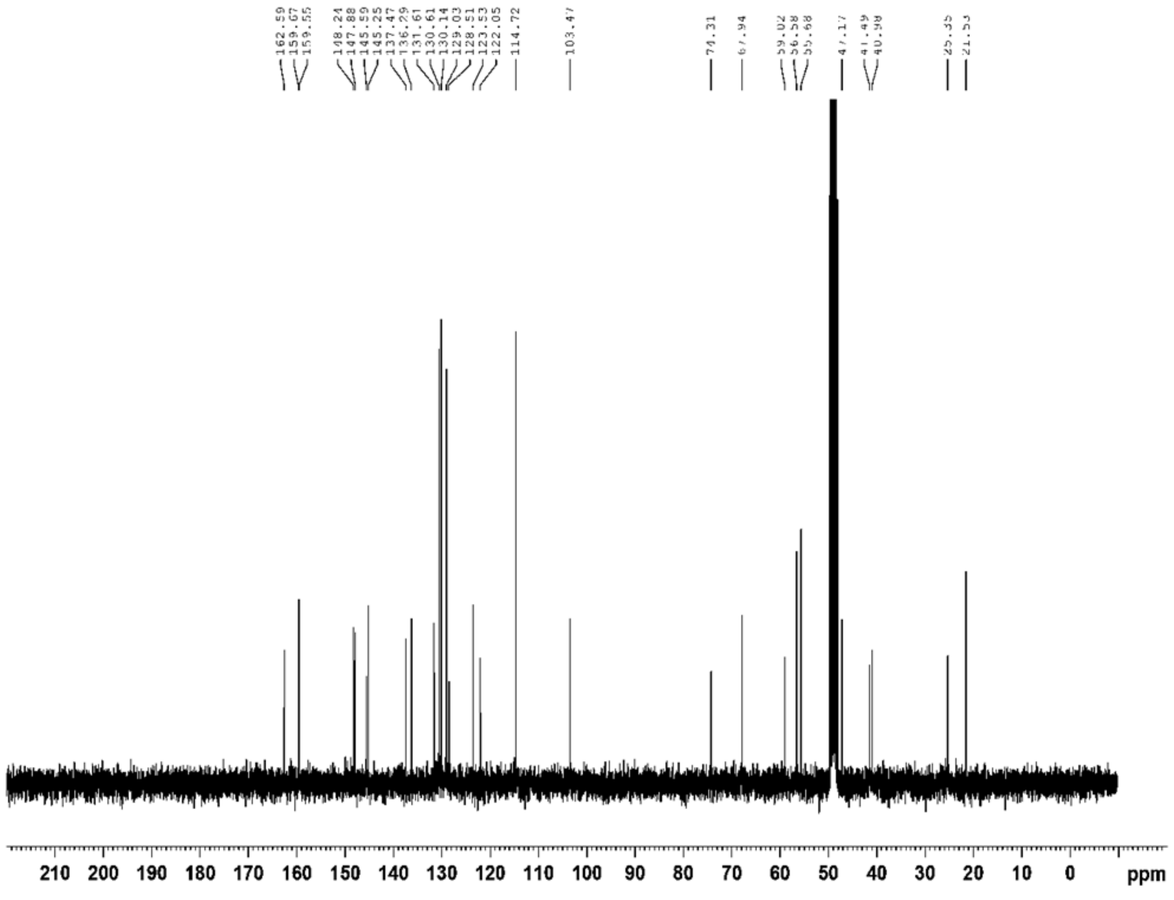
162.59
159.67
159.55
148.24
147.88
145.59
145.25
137.47
136.29
131.61
130.61
130.14
129.03
128.51
123.53
122.05
114.72
103.47
74.31
67.94
59.02
56.58
55.68
47.17
41.49
40.98
25.35
21.53
210
200
190
180
170
160
150
140
130
120
110
100
90
80
70
60
50
40
30
20
10
0
ppm

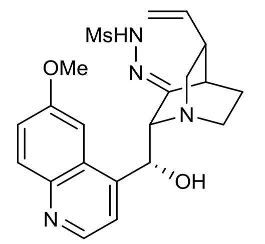

MsHN
N
OMe
N
OH
N

14

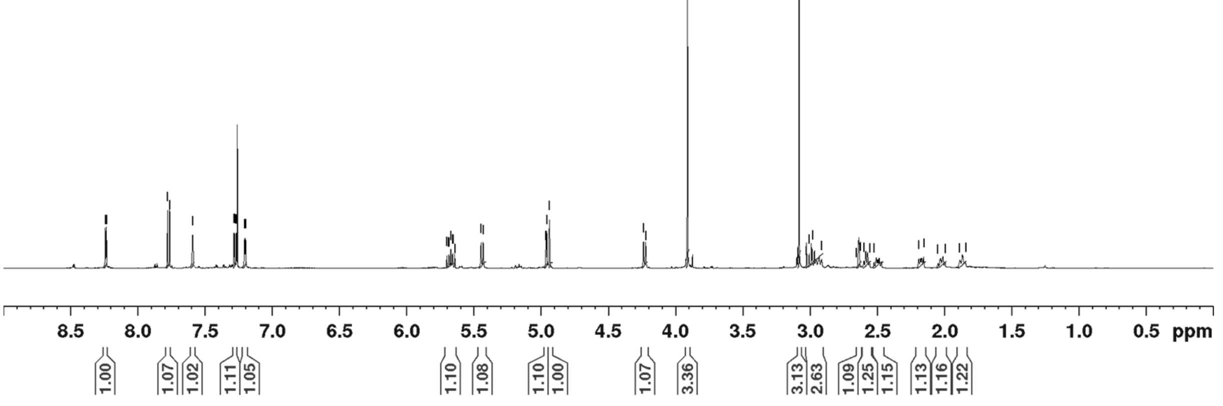

8.24
8.23
7.78
7.76
7.59
7.59
7.29
7.28
7.27
7.27
7.26
7.21
7.20
5.70
5.69
5.69
5.67
5.66
5.66
5.64
5.45
5.43
4.97
4.96
4.96
4.94
4.24
4.22
3.91
3.08
3.03
3.01
2.98
2.91
2.66
2.63
2.60
2.56
2.52
2.19
2.15
2.05
1.99
1.89
1.84
8.5 8.0 7.5 7.0 6.5 6.0 5.5 5.0 4.5 4.0 3.5 3.0 2.5 2.0 1.5 1.0 0.5 ppm
1.00
1.07
1.02
1.11
1.05
1.10
1.08
1.10
1.00
1.07
3.36
3.13
2.63
1.09
1.25
1.15
1.13
1.16
1.22

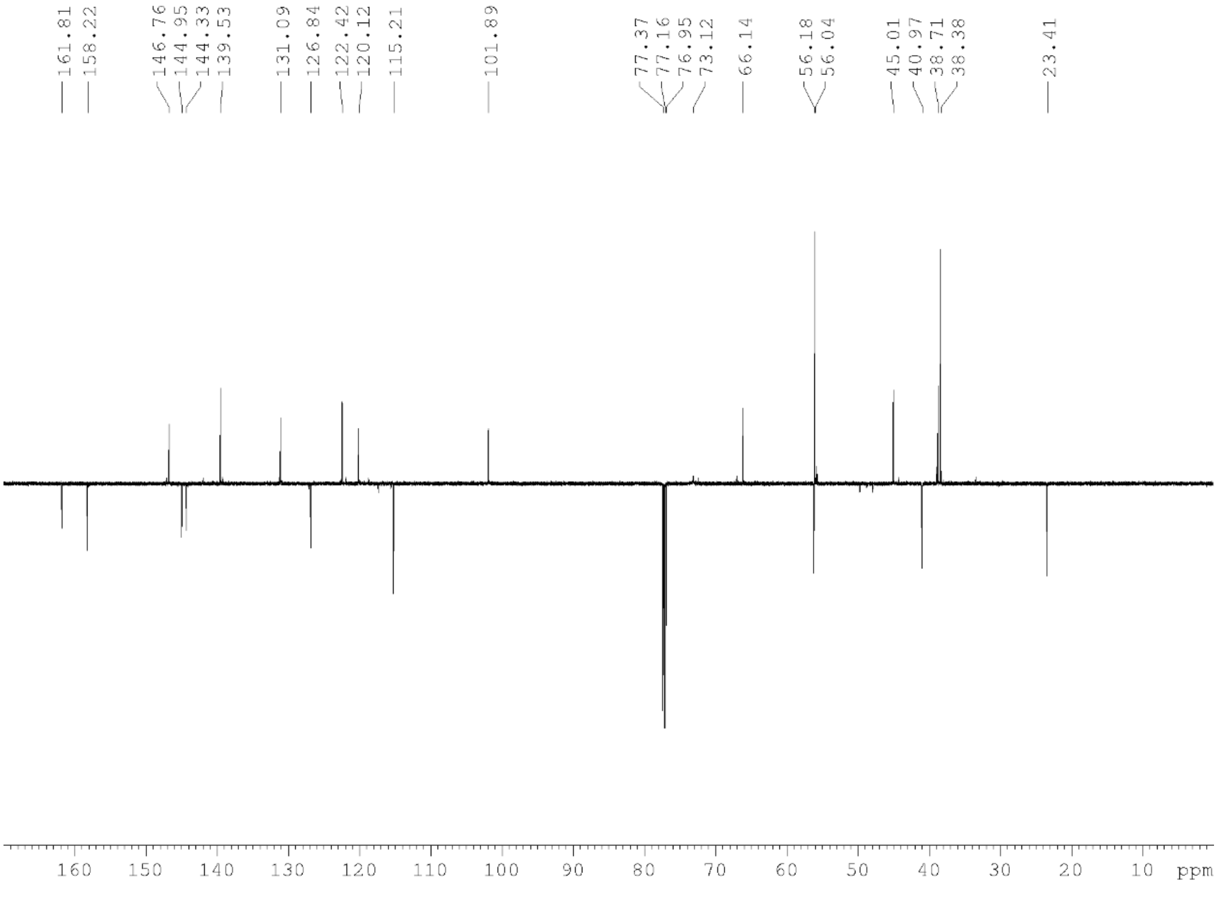

161.81
158.22
146.76
144.95
144.33
139.53
131.09
126.84
122.42
120.12
115.21
101.89
77.37
77.16
76.95
73.12
66.14
56.18
56.04
45.01
40.97
38.71
38.38
23.41
160 150 140 130 120 110 100 90 80 70 60 50 40 30 20 10 ppm

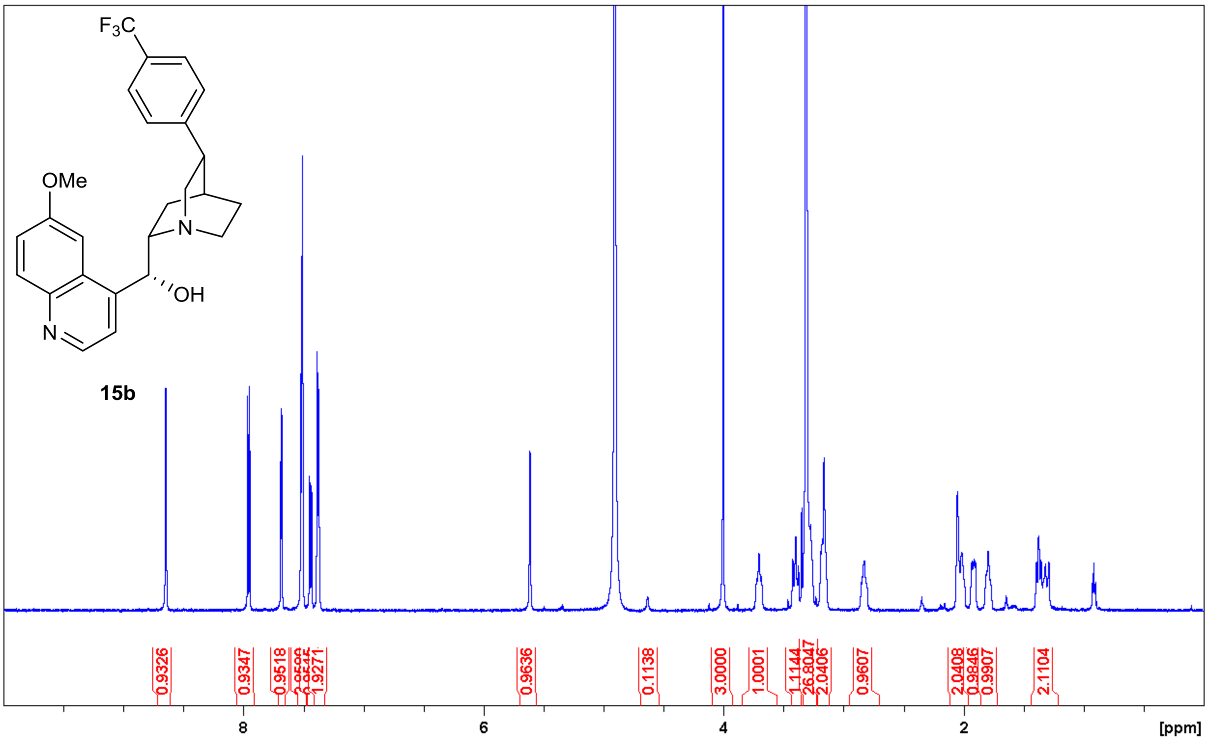

F3C
OMe
N
OH
N
15b
0.9326
0.9347
0.9518
1.9271
0.9636
0.1138
3.0000
1.0001
1.1144
26.8047
2.0406
0.9607
2.0408
0.9846
0.9907
2.1104
8
6
4
2
[ppm]

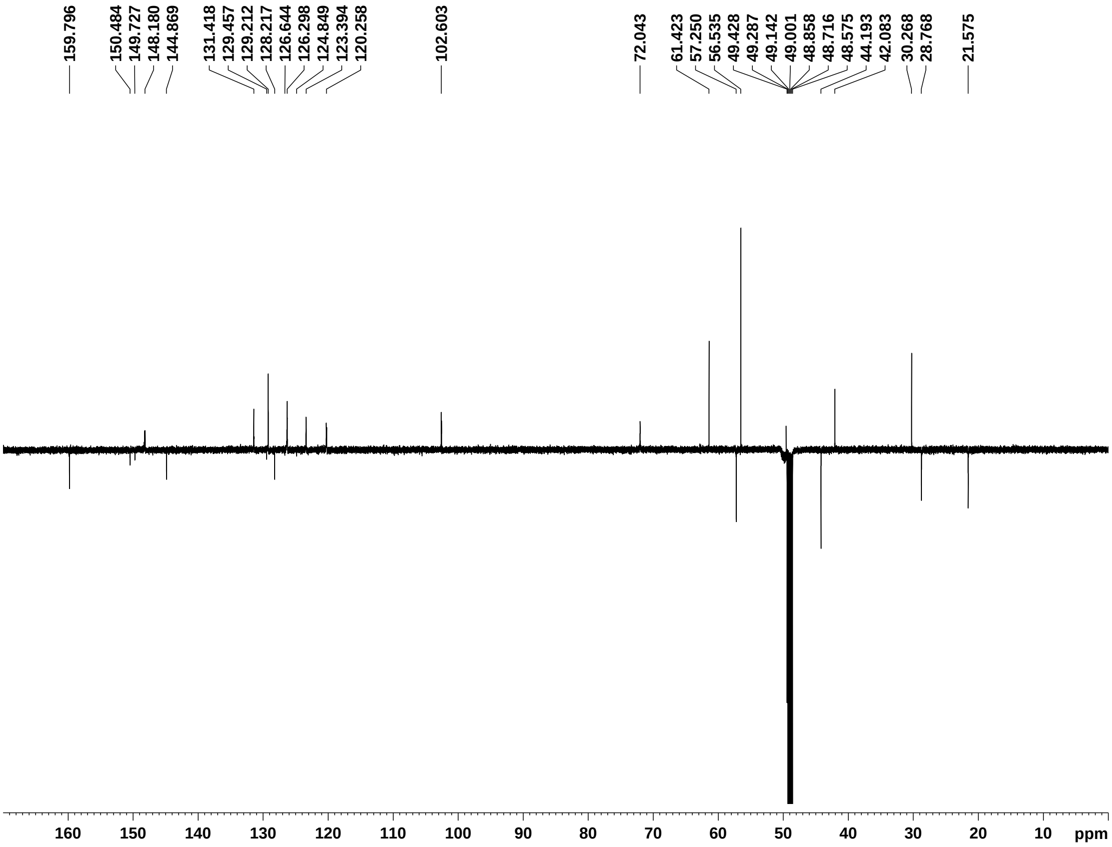

159.796
150.484
149.727
148.180
144.869
131.418
129.457
129.212
128.217
126.644
126.298
124.849
123.394
120.258
102.603
72.043
61.423
57.250
56.535
49.428
49.287
49.142
49.001
48.858
48.716
48.575
44.193
42.083
30.268
28.768
21.575
160
150
140
130
120
110
100
90
80
70
60
50
40
30
20
10
ppm

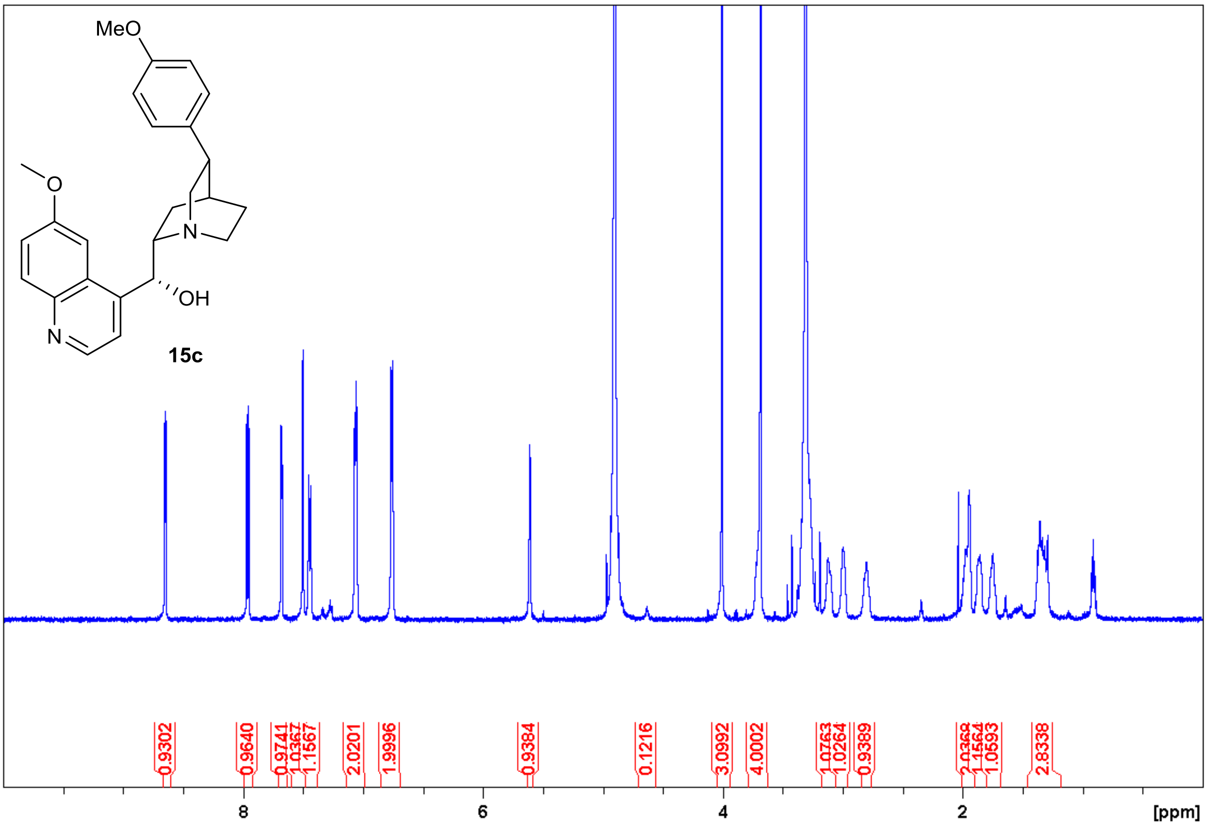
MeO
O
N
N
OH
15c
0.9302
0.9640
0.9741
1.0367
1.1567
2.0201
1.9996
0.9384
0.1216
3.0992
4.0002
1.0763
1.0264
0.9389
2.0369
1.1564
1.0593
2.8338
8
6
4
2
[ppm]

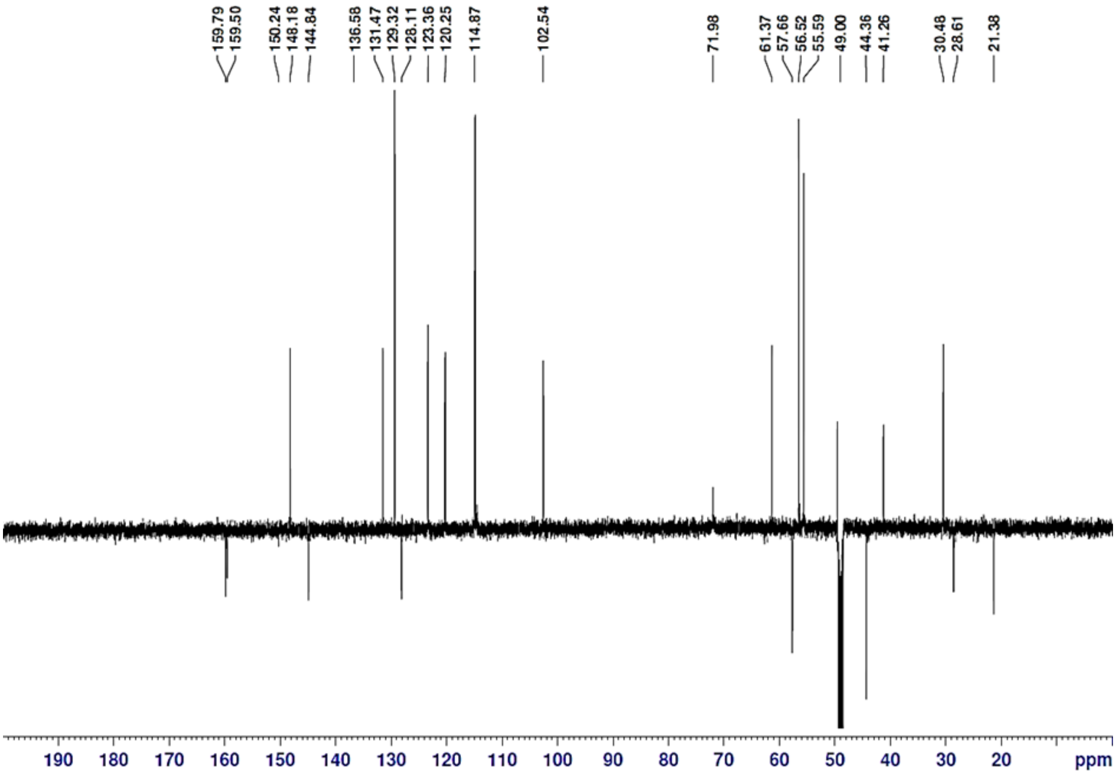
159.79
159.50
150.24
148.18
144.84
136.58
131.47
129.32
128.11
123.36
120.25
114.87
102.54
71.98
61.37
57.66
56.52
55.59
49.00
44.36
41.26
30.48
28.61
21.38
190
180
170
160
150
140
130
120
110
100
90
80
70
60
50
40
30
20
ppm

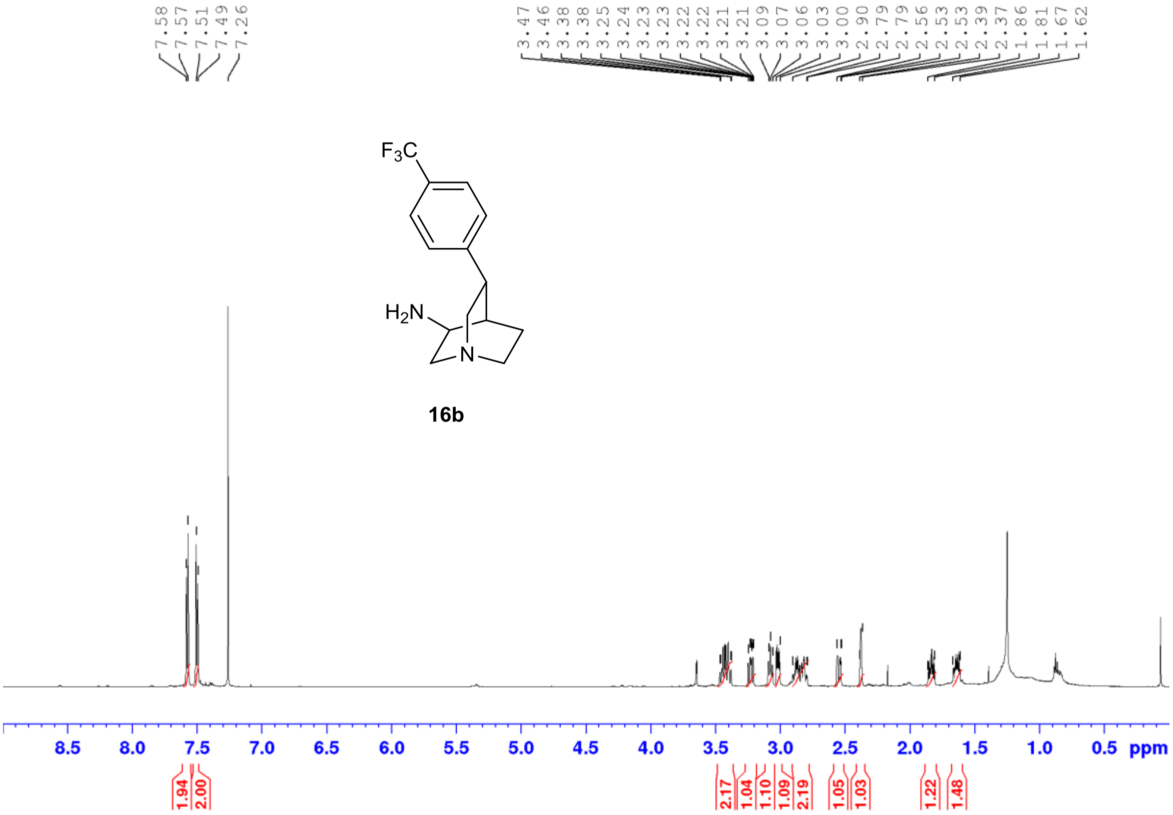

7.58
7.57
7.51
7.49
7.26
3.47
3.46
3.38
3.38
3.25
3.24
3.23
3.23
3.22
3.22
3.21
3.09
3.07
3.06
3.03
3.00
2.90
2.79
2.79
2.56
2.53
2.53
2.39
2.37
1.86
1.81
1.67
1.62
F3C
H2N
N
8.5
8.0
7.5
7.0
6.5
6.0
5.5
5.0
4.5
4.0
3.5
3.0
2.5
2.0
1.5
1.0
0.5
ppm
1.94
2.00
2.17
1.04
1.10
1.09
2.19
1.05
1.03
1.22
1.48

16b

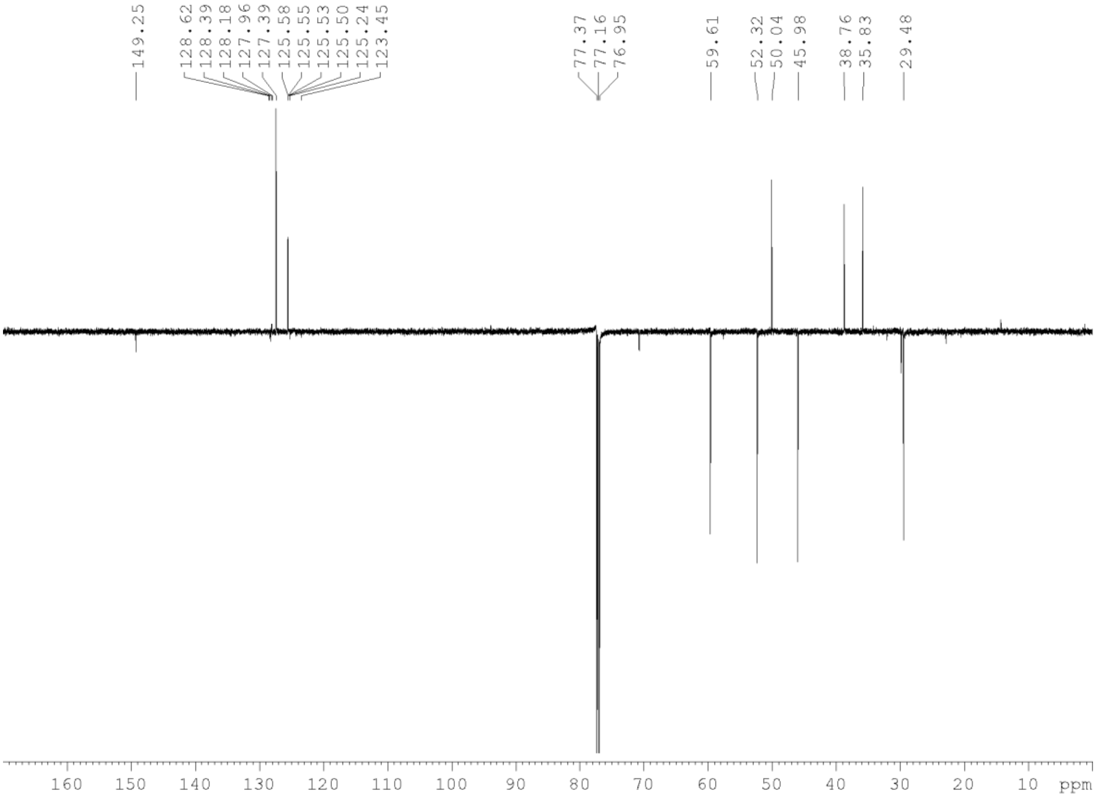

149.25
128.62
128.39
128.18
127.96
127.39
125.58
125.55
125.53
125.50
125.24
123.45
77.37
77.16
76.95
59.61
52.32
50.04
45.98
38.76
35.83
29.48
160
150
140
130
120
110
100
90
80
70
60
50
40
30
20
10
ppm

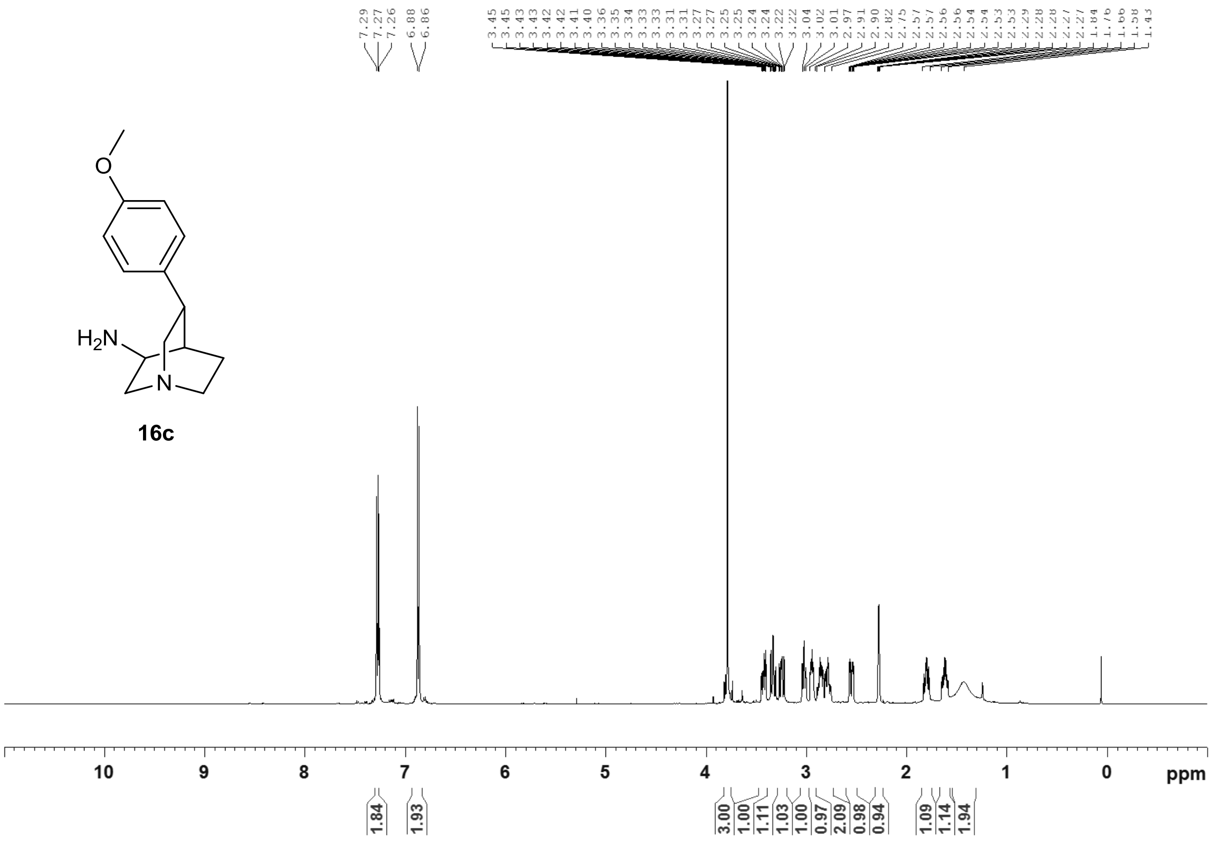
7.29
7.27
7.26
6.88
6.86
3.45 3.45 3.43 3.43 3.42 3.41 3.40 3.36 3.35 3.34 3.33 3.33 3.31 3.31 3.27 3.27 3.25 3.25 3.24 3.24 3.22 3.22 3.04 3.02 3.01 2.97 2.91 2.90 2.82 2.75 2.57 2.57 2.56 2.54 2.54 2.53 2.53 2.29 2.28 2.27 2.27 1.84 1.76 1.66 1.58 1.43
O
H2N
N
16c
10 9 8 7 6 5 4 3 2 1 0 ppm
1.84 1.93 3.00 1.00 1.11 1.03 1.00 0.97 2.09 0.98 0.94 1.09 1.14 1.94

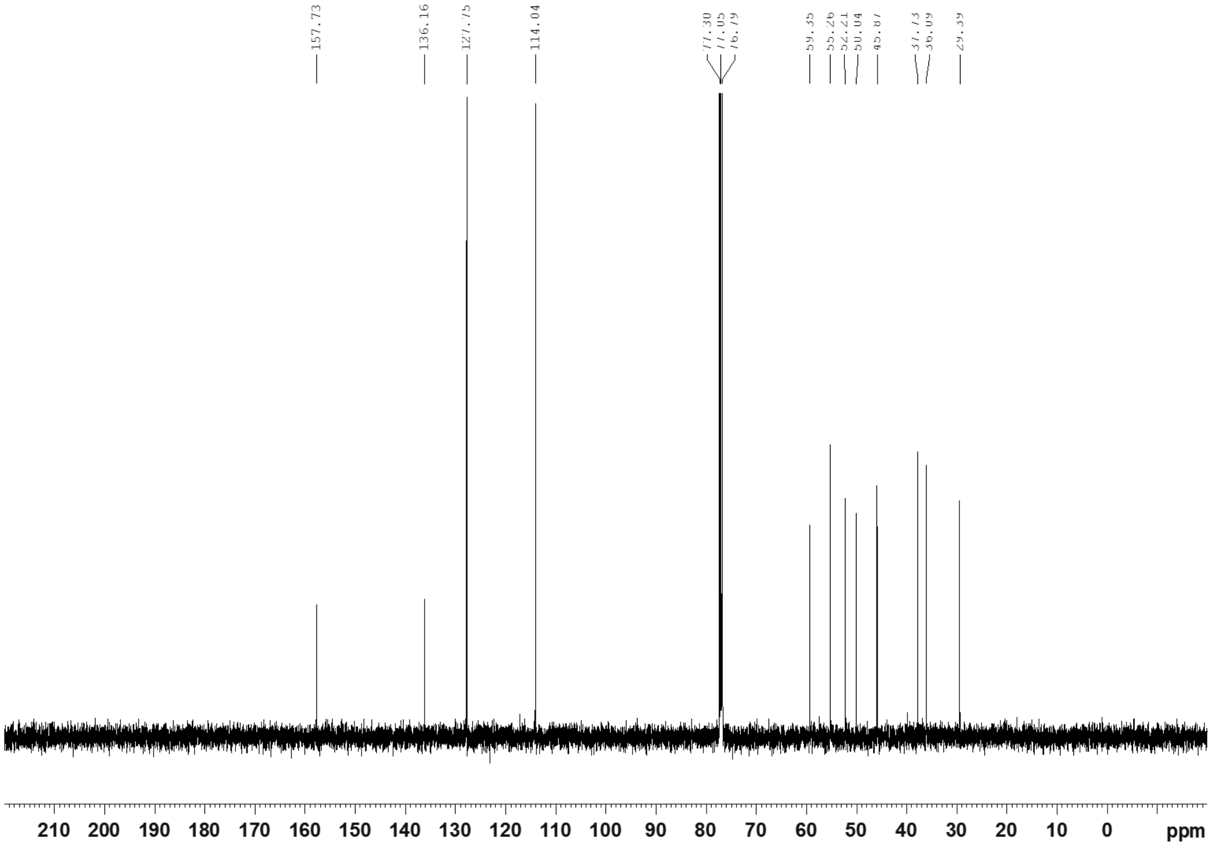
157.73
136.16
127.75
114.04
77.30 77.05 76.79
59.35 55.26 52.21 50.04 45.87
37.73 36.09
29.39
210 200 190 180 170 160 150 140 130 120 110 100 90 80 70 60 50 40 30 20 10 0 ppm

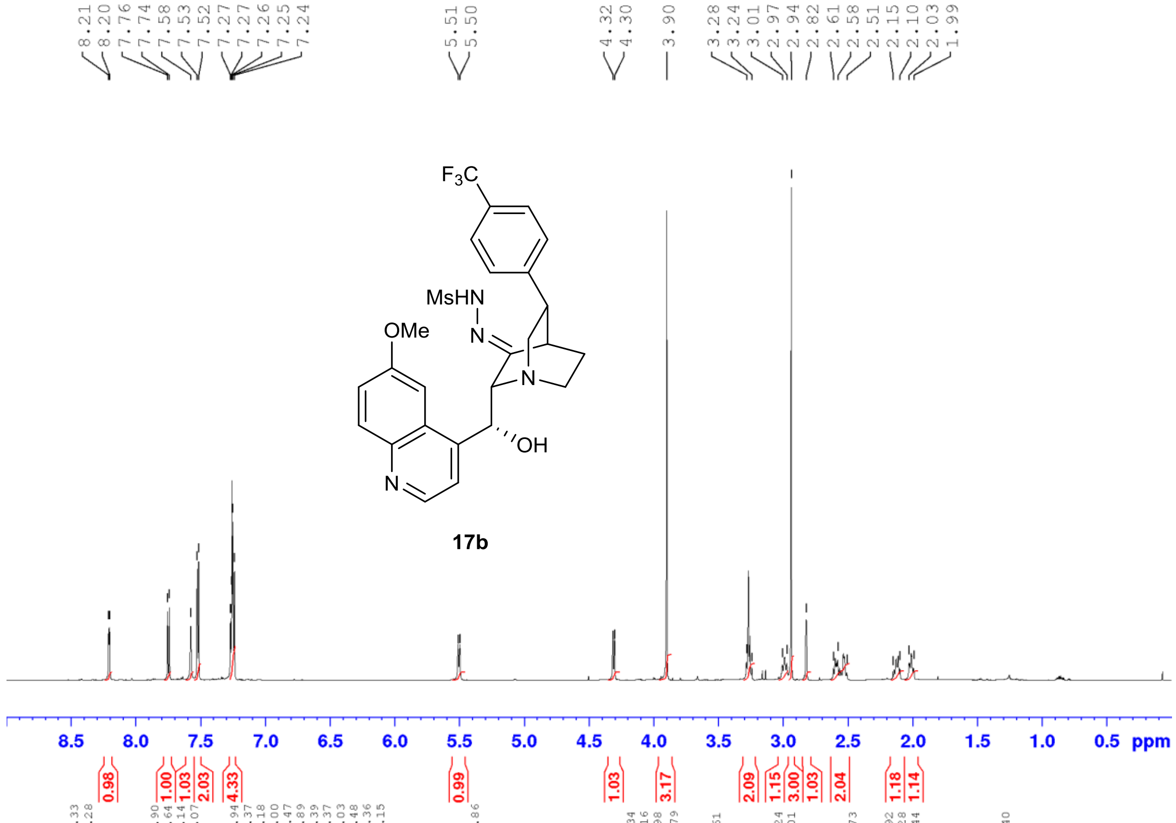
8.21
8.20
7.76
7.74
7.58
7.53
7.52
7.27
7.27
7.26
7.25
7.24
5.51
5.50
4.32
4.30
3.90
3.28
3.24
3.01
2.97
2.94
2.82
2.61
2.58
2.51
2.15
2.10
2.03
1.99
F3C
MsHN
N
OMe
N
OH
N
17b
0.98
1.00
1.03
2.03
4.33
0.99
1.03
3.17
2.09
1.15
3.00
1.03
2.04
1.18
1.14
ppm

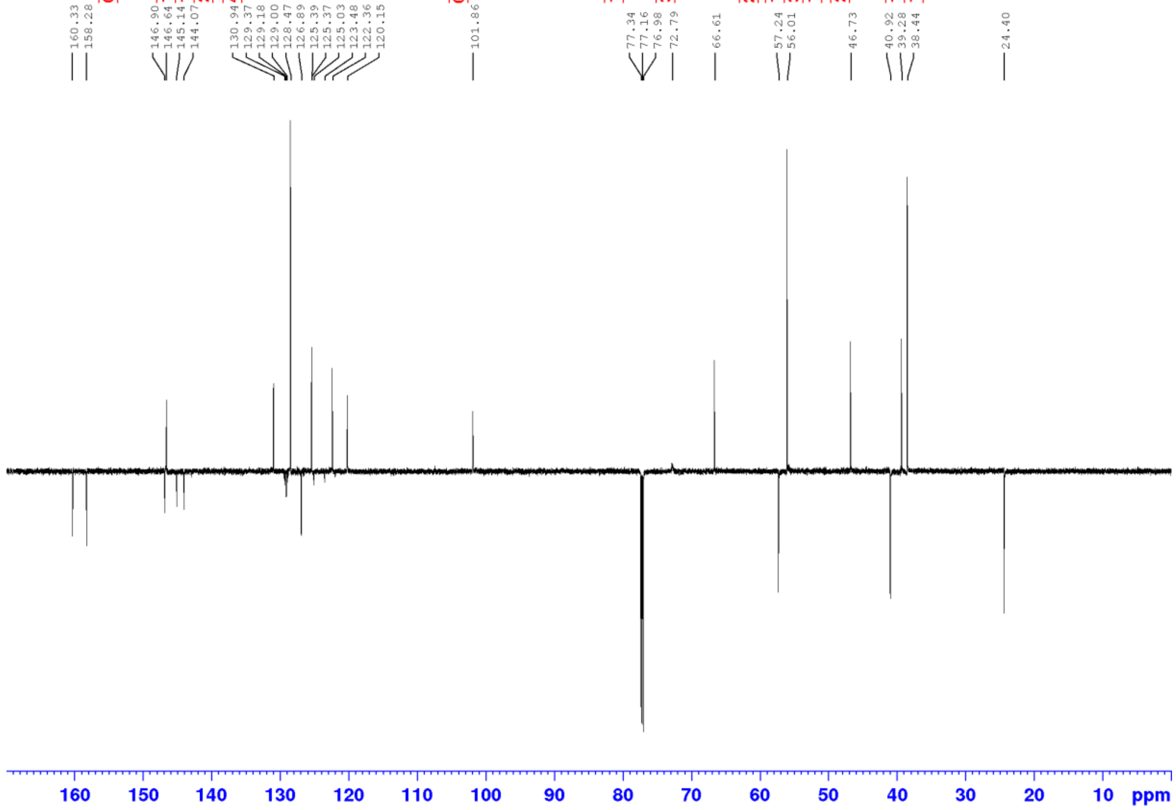
160.33
158.28
146.90
146.64
145.14
144.07
130.94
129.37
129.18
129.00
128.47
126.89
125.39
125.37
125.03
123.48
122.36
120.15
101.86
77.34
77.16
76.98
72.79
66.61
57.24
56.01
46.73
40.92
39.28
38.44
24.40
ppm

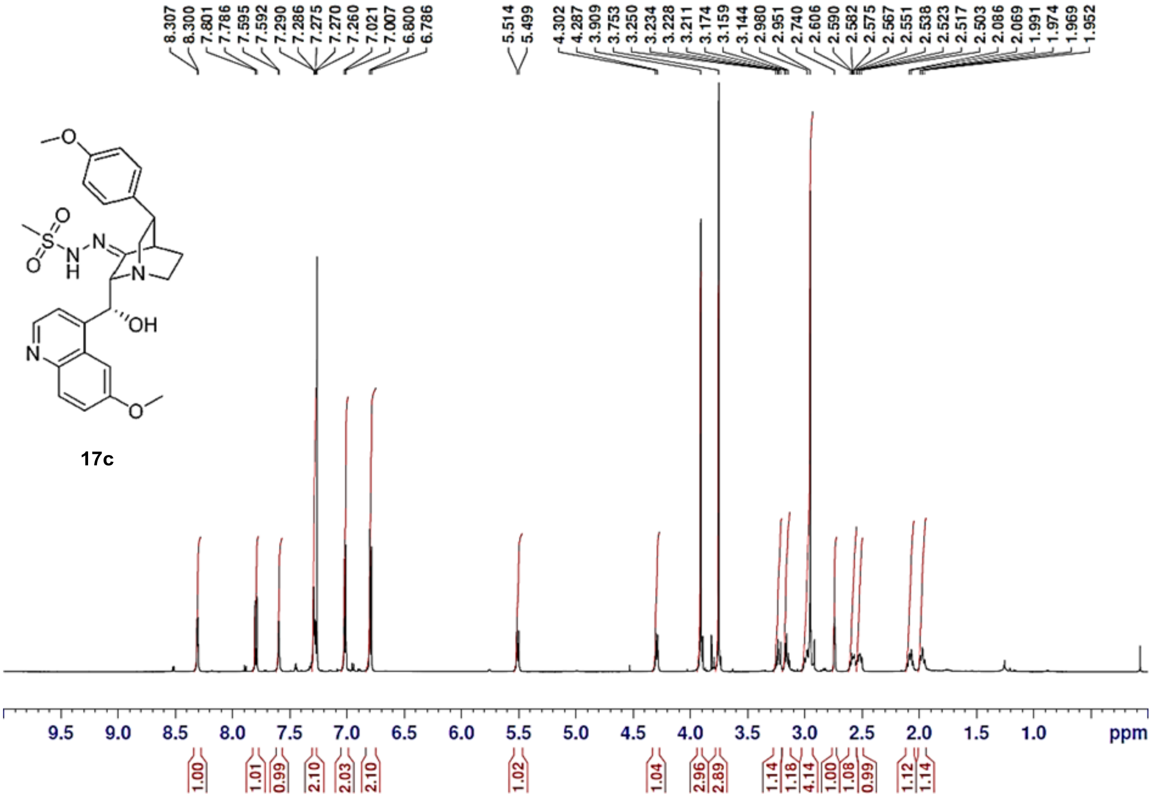
17c
8.307
8.300
7.801
7.786
7.595
7.592
7.290
7.286
7.275
7.270
7.260
7.021
7.007
6.800
6.786
5.514
5.499
4.302
4.287
3.909
3.753
3.250
3.234
3.228
3.211
3.174
3.159
3.144
2.980
2.951
2.740
2.606
2.590
2.582
2.575
2.567
2.551
2.538
2.523
2.517
2.503
2.086
2.069
1.991
1.974
1.969
1.952
9.5 9.0 8.5 8.0 7.5 7.0 6.5 6.0 5.5 5.0 4.5 4.0 3.5 3.0 2.5 2.0 1.5 1.0 ppm
1.00
1.01
0.99
2.10
2.03
2.10
1.02
1.04
2.96
2.89
1.14
1.16
4.14
1.00
1.08
0.99
1.12
1.14

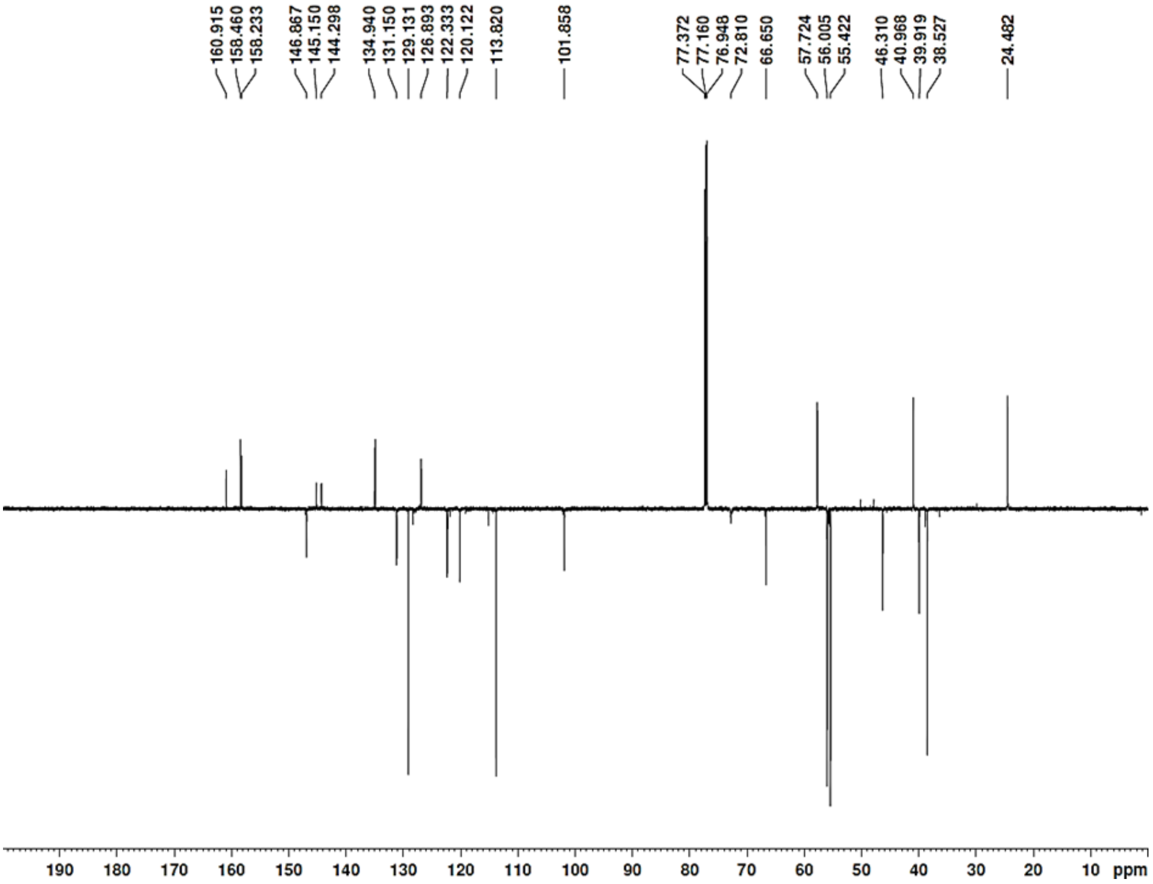
160.915
158.460
158.233
146.867
145.150
144.298
134.940
131.150
129.131
126.893
122.333
120.122
113.820
101.858
77.372
77.160
76.948
72.810
66.650
57.724
56.005
55.422
46.310
40.968
39.919
38.527
24.482
190 180 170 160 150 140 130 120 110 100 90 80 70 60 50 40 30 20 10 ppm

Comparison of commercial Quinine (1), in red, and the herein synthesized one, in blue:

^{1}H NMR (400 MHz, DMSO-d$_6$)

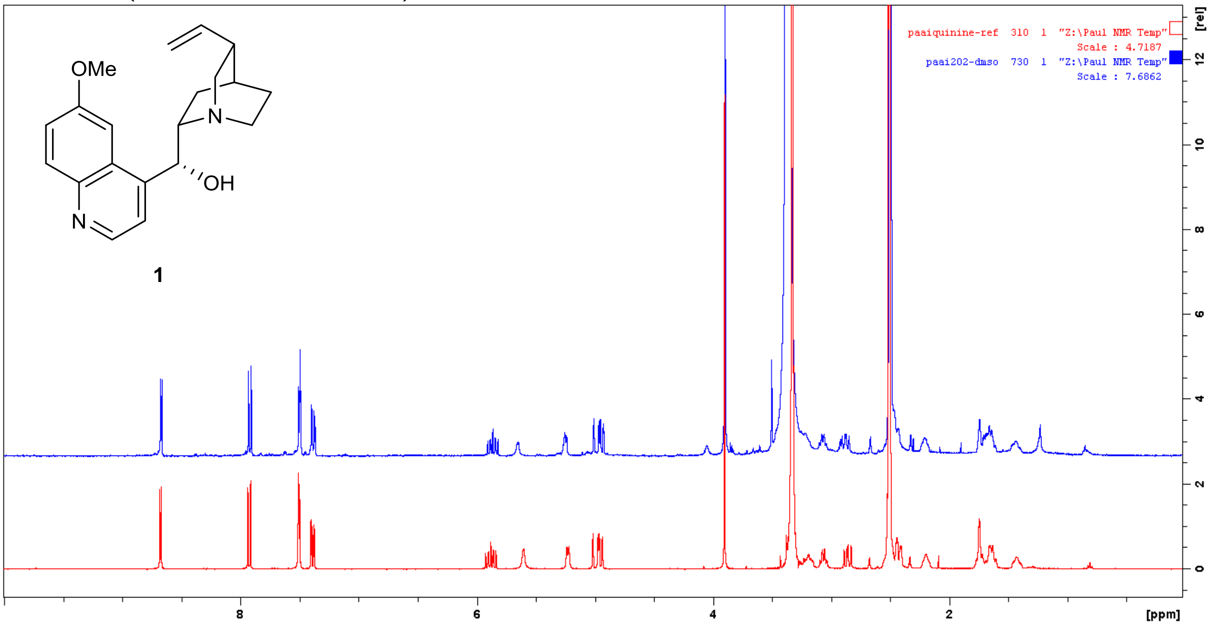

^{13}C NMR (100 MHz, DMSO-d$_6$)

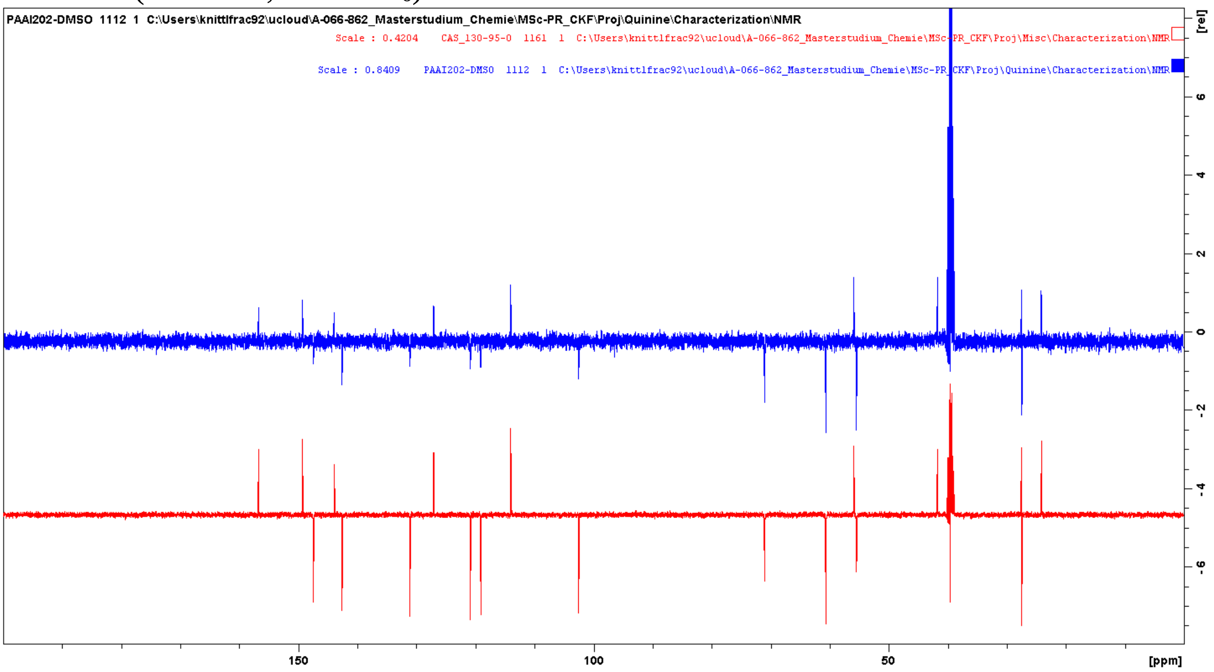

Peak table of commercial Quinine (1) and the herein synthesized one:

	δ/ppm	
Peak	Commercial	Synthesized
1	156.7	156.7
2	149.3	149.3
3	147.5	147.4
4	143.9	143.9
5	142.6	142.6
6	131.1	131.1
7	127.1	127.1
8	120.9	120.9
9	119.1	119.1
10	114.0	114.0
11	102.5	102.5
12	71.0	71.0
13	60.6	60.7
14	55.9	55.9
15	55.5	55.4
16	41.8	41.7
20	39.6	39.6
25	27.5	27.5
26	27.4	27.4
27	24.1	24.2

Chromatographic Separation of Racemic Quinine

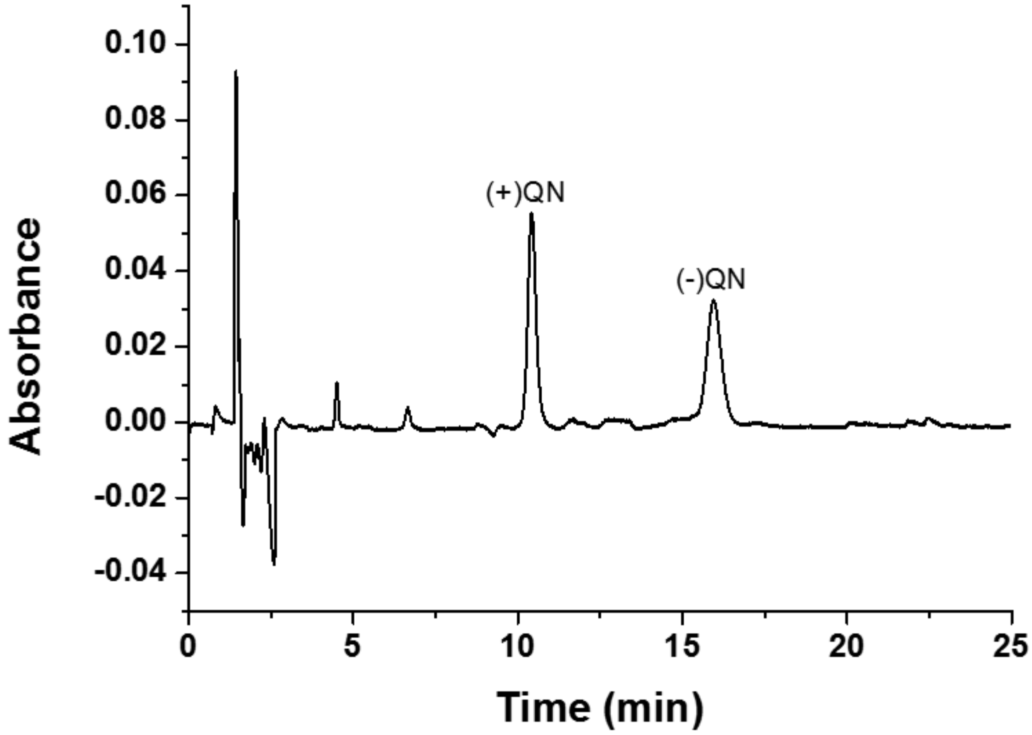

Chromatographic conditions: Chiralpak IA; eluent, CO_2/MeOH (90/10 *v*/*v*) containing 20 mM DEA; flow, 2 ml min^{-1}; detection, 215–230 nm; temperature, $T = 40$ °C; back pressure, 150 bar; SFC mode.

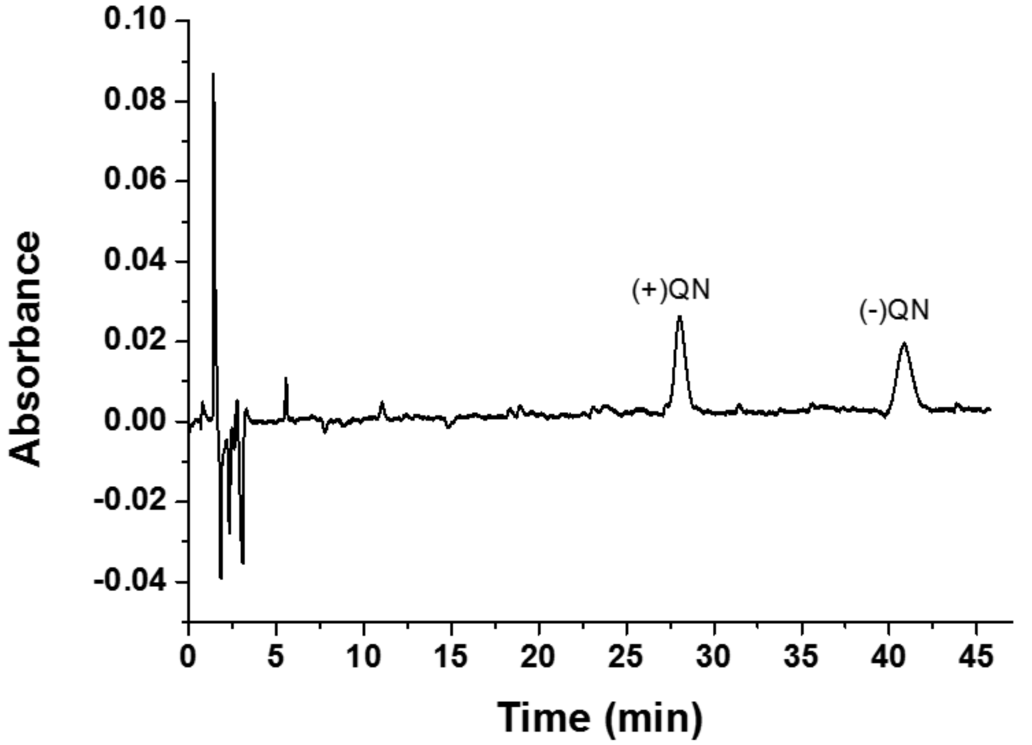

Chromatographic conditions: Chiralpak IG; eluent, CO_2/MeOH (90/10 *v*/*v*) containing 20 mM DEA; flow, 2 ml min^{-1}; detection, 215–230 nm; temperature, $T = 40$ °C; back pressure, 150 bar; SFC mode.

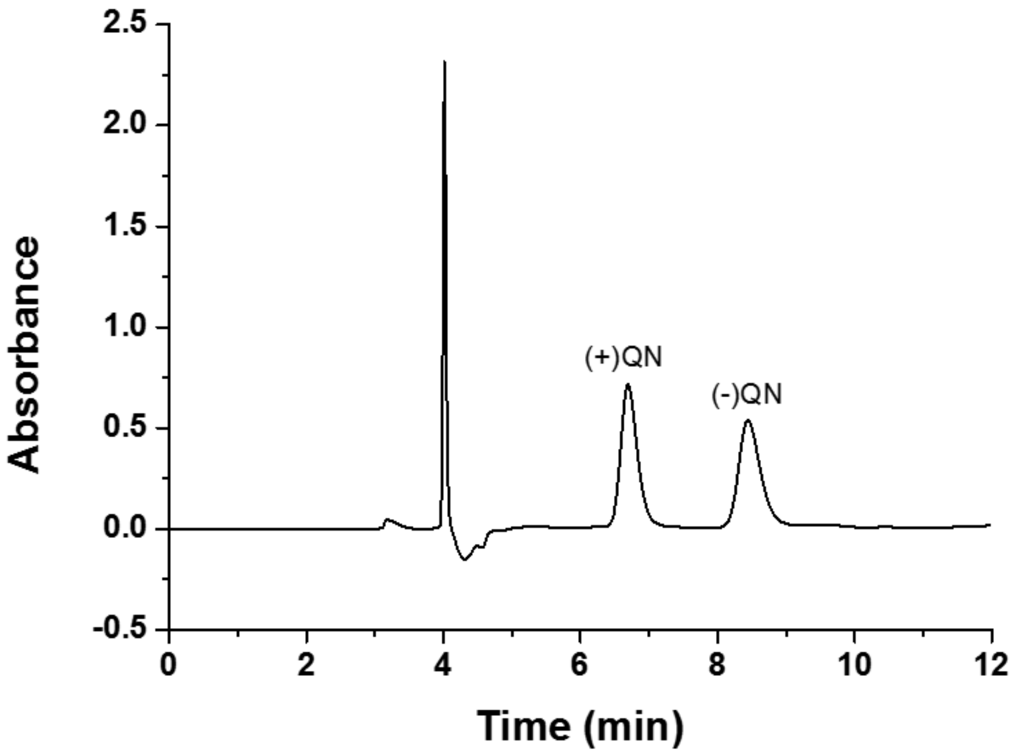

Chromatographic conditions: Chiralpak IC; eluent, *n*-hexane/2-PrOH/DEA (80/20/0.1 *v*/*v*/*v*); flow, 1 ml min^{-1}; detection, 230–250 nm; temperature, T = 30 °C; HPLC, normal phase mode.

Biological Testing

Supplementary Table 1: *In vitro* anti-protozoal activity of (−)-Quinine, (+)-Quinine (**1**) and novel analogues (±)-**15b,c**.

Substance	IC_{50} against *P. falciparum* (strain NF54) (nM)[a]	IC_{50} for cytotoxicity (strain L6) (µM)[a]
Chloroquine	6 ± 3[b]	—
Podophyllotoxine	—	0.010 ± 0.002[b]
(−)-Quinine (**1**)	22 ± 3[b]	111 ± 21[c]
(+)-Quinine (**1**)	122 ± 3[b]	142 ± 21[c]
(±)-**15b**	5 ± 5[b]	7 ± 3[b]
(±)-**15c**	12 ± 15[b]	16 ± 2[b]

[a] The values are given as mean ± standard deviation. [b] 3 replicates. [c] 2 replicates.

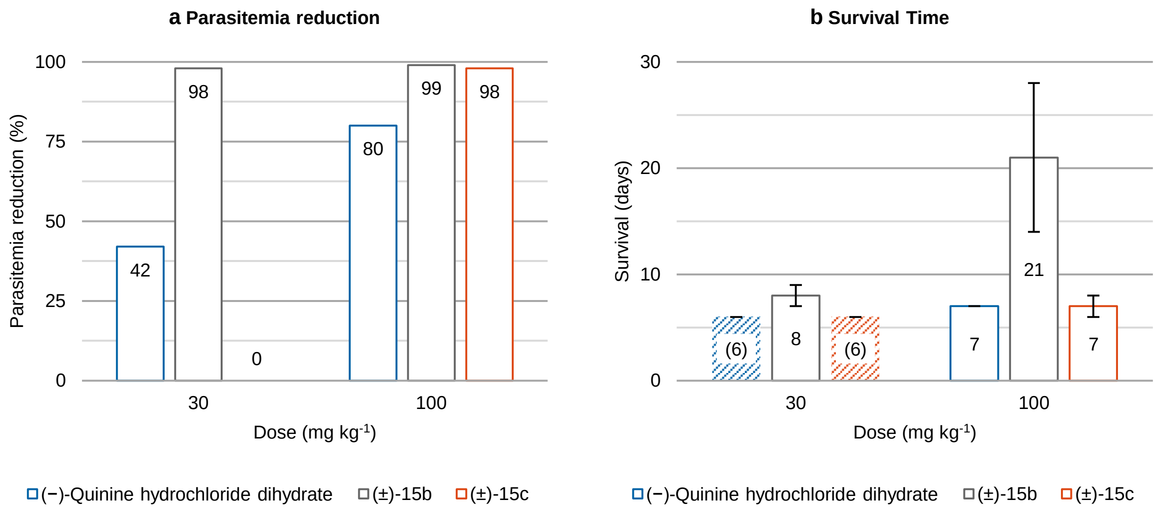

Supplementary Figure 1: *In vivo* screening against *P. berghei*. Tested substances were commercial quinine hydrochloride (Sigma Q1125) versus aryl analogues (±)-**15b,c** (area purity > 99%, *vide infra*) on groups of 3 mice infected with *P. berghei*. a) Parasitemia reduction given as mean determined 3 days after single dose administration of 30 and 100 mg kg^{-1} respectively. b) Survival time in days given as mean ± standard deviation after single dose administration of 30 and 100 mg kg^{-1} respectively. Striped bars represent mice with parasitemia reduction <50% which were euthanized on day 3 postinfection in order to prevent death otherwise occurring on day 6.

Methods

In vitro assays

The *in vitro* antiprotozoal activities against *P. falciparum* and cytotoxicity assessment against L6 cells were determined as reported elsewhere.[1] The following strains, parasite forms and positive controls were used: *P. falciparum*, NF54 erythrocytic stages, chloroquine, IC_{50} of 6 nM and L6 cells, rat skeletal myoblasts, podophyllotoxin, IC_{50} of 0.010 µM.

In *vivo* assays

The *in vivo* antimalarial activity was assessed basically as previously described.[2] Groups of three female NMRI mice (20–22 g) intravenously infected with 2×10^7 parasitized erythrocytes on day 0 with GFP-transfected *P. berghei* strain ANKA.[3] Compounds were formulated in Tween 80/Ethanol (70%/30%), diluted 10-fold in distilled water and administered orally in a volume of 10 ml kg^{-1} as a single dose (24 h post infection). Parasitaemia was determined on day 3 post infection by FACS analysis. Activity was calculated as the difference between the mean per cent parasitaemia for the control (n = 5 mice) and treated groups expressed as a per cent relative to the control group. The survival time in days was also recorded up to 30 days after infection. A compound was considered curative if the animal survived to day 30 after infection with no detectable parasites. In vivo efficacy studies in mice were conducted at the Swiss Tropical and Public Health Institute (Basel) according to the rules and regulations for the protection of animal rights ("Tierschutzverordnung") of the Swiss "Bundesamt für

[1] Orhan, I., Sener, B., Kaiser, M., Brun, R. & Tasdemir, D. *Mar. Drugs* **2010**, *8*, 47-58.
[2] Peters, W. *Chemotherapy and drug resistance in malaria*. 2 edn, Vol. 1 (Academic Press, 1987).
[3] Franke-Fayard, B. *et al. Mol. Biochem. Parasitol.* **2004,** *137*, 23-33.

Veterinärwesen". They were approved by the veterinary office of Canton Basel-Stadt, Switzerland.

X-Ray Analysis

The X-ray intensity data were measured on Bruker X8 APEXII diffractometers equipped with multilayer monochromators, Mo K/a INCOATEC micro focus sealed tube and Kryoflex cooling devices. The structures were solved by direct methods and refined by full-matrix least-squares techniques. Non-hydrogen atoms were refined with anisotropic displacement parameters. Hydrogen atoms were inserted at calculated positions and refined with a riding model or as rotating groups. The following software was used: Frame integration, Bruker SAINT software package[i] using a narrow-frame algorithm, Absorption correction, SADABS[ii], structure solution, SHELXL–2013[iii], refinement, SHELXL–2013[iii], OLEX2[iv], ShelXle[v], molecular diagrams, OLEX2[iv]. Experimental data and CCDC-Code can be found in Supplementary Table 2.

Supplementary Table 2: Experimental parameters and CCDC-Code.

Sample	Machine	Source	Temp.	Detector Distance	Time/ Frame	#Frames	Frame width	CCDC
	Bruker		[K]	[mm]	[s]		[°]	
**TBS-13**	X8	Mo	130	35	10	3469	0.5	1500944

Hydrazone TBS-13

Metrical parameters for structure **TBS-13** are available free of charge from the Cambridge Crystallographic Data Centre (CCDC) under reference number 1500944. Crystal data, data collection parameters, and structure refinement details are given in Supplementary Table 3 and Supplementary Table 4. Molecular Structure in “Ortep View” is displayed in Supplementary Figure 2.

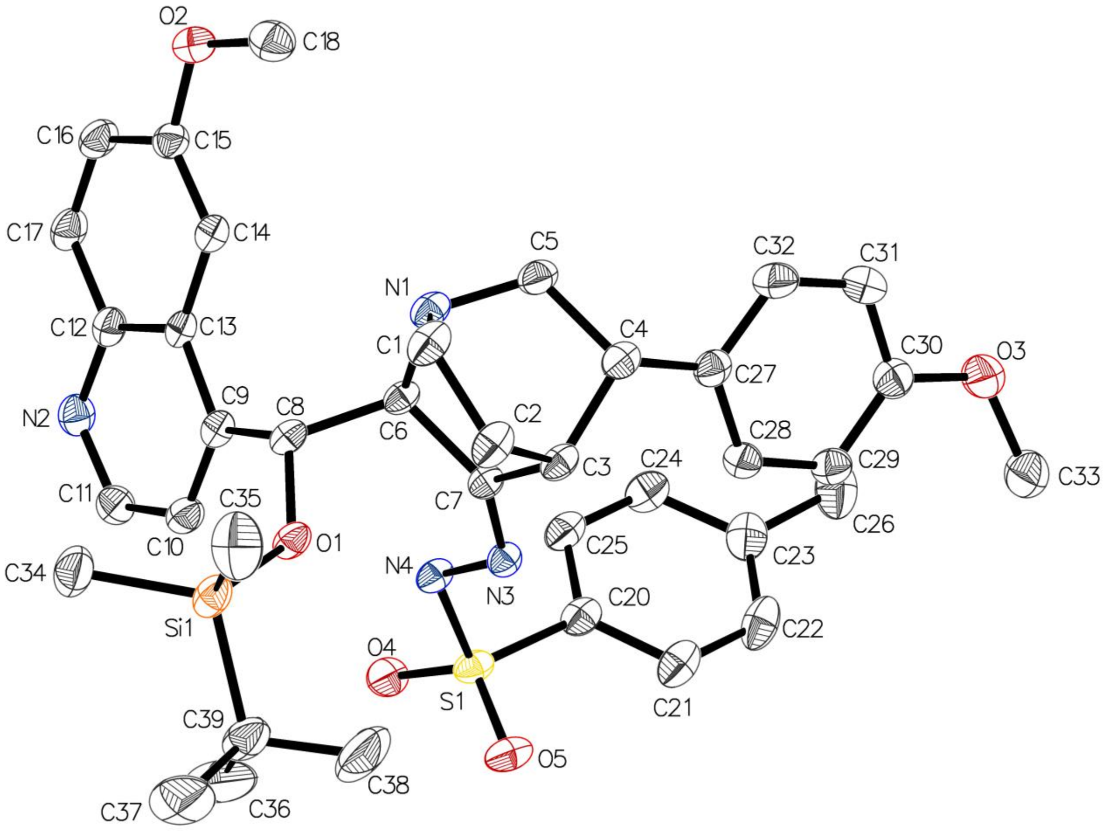

Supplementary Figure 2: Asymmetric unit of hydrazone **TBS-13**, drawn with 50% displacement ellipsoids. Hydrogen atoms omitted for clarity.

Supplementary Table 3: Sample and crystal data of hydrazone **TBS-13**.

**Chemical formula**	C39H52N4O6SSi	**Crystal system**	Triclinic	
**Formula weight [g/mol]**	732.99	**Space group**	*P–1*	
**Temperature [K]**	130	**Z**	2	
**Measurement method**	\Φ and \ω scans	**Volume [Å^3]**	1932.32(14)	
**Radiation (Wavelength [Å])**	MoKα (λ = 0.71073)	**Unit cell dimensions [Å] and [°]**	10.5942(4)	73.965(2)
**Crystal size / [mm^3]**	0.22 × 0.17 × 0.05		12.0738(5)	77.888(2)
**Crystal habit**	clear colourless block		16.0765(7)	87.087(2)
**Density (calculated) / [g/cm^3]**	1.26	**Absorption coefficient / [mm^{-1}]**	0.7460	
**Abs. correction T_{min}**	0.5579	**Abs. correction T_{max}**	0.746	
**Abs. correction type**	multiscan	**F(000) [e$^-$]**	784	

Supplementary Table 4: Data collection and structure refinement of **TBS-13**.

**Index ranges**	$-14 \leq h \leq 14$, $-17 \leq k \leq 17$, $-22 \leq l \leq 22$	**Theta range for data collection [°]**	2.692 to 60.212	
**Reflections number**	76086	**Data / restraints / parameters**	11320/0/474	
**Refinement method**	Least squares	**Final R indices**	all data	R1 = 0.0641, wR2 = 0.1243
**Function minimized**	$\Sigma\, w(F_o^2 - F_c^2)^2$		$I > 2\sigma(I)$	R1 = 0.0452, wR2 = 0.1130
**Goodness-of-fit on F^2**	1.049	**Weighting scheme**	$w=1/[\sigma^2(F_o^2)+(0.0485P)^2+ 0.8424P]$	
**Largest diff. peak and hole [e Å^{-3}]**	0.41/−0.44		where $P=(F_o^2+2F_c^2)/3$	

[i] Bruker, *SAINT*. Bruker AXS Inc., Madison, Wisconsin, USA (2012).

[ii] G. M. Sheldrick, *SADABS*. University of Göttingen, Germany (1996).

[iii] G. M. Sheldrick, A short history of SHELX. *Acta Crystallogr A* **64**, 112–122 (2008).

[iv] O. V. Dolomanov, L. J. Bourhis, R. J. Gildea, J. A. K. Howard, H. Puschmann, OLEX2: a complete structure solution, refinement and analysis program. *J.Appl. Crystallogr.* **42**, 339–341 (2009).

[v] C. B. Hubschle, G. M. Sheldrick, B. Dittrich, ShelXle: a Qt graphical user interface for SHELXL. *J. Appl. Crystallogr.* **44**, 1281–1284 (2011).
